# Comparative Transcriptome Profiles of Near-Isogenic Hexaploid Wheat Lines Differing for Effective Alleles at the 2DL FHB Resistance QTL

**DOI:** 10.3389/fpls.2018.00037

**Published:** 2018-01-30

**Authors:** Chiara Biselli, Paolo Bagnaresi, Primetta Faccioli, Xinkun Hu, Margaret Balcerzak, Maria G. Mattera, Zehong Yan, Therese Ouellet, Luigi Cattivelli, Giampiero Valè

**Affiliations:** ^1^CREA–Research Centre for Genomics and Bioinformatics, Fiorenzuola d'Arda, Italy; ^2^Ottawa Research and Development Centre, Agriculture and Agri-Food Canada, Ottawa, ON, Canada; ^3^Triticeae Research Institute, Sichuan Agricultural University, Chengdu, China; ^4^Plant Breeding Department, Institute for Sustainable Agriculture, Cordoba, Spain; ^5^Department of Genetics–ETSIAM, University of Cordoba, Cordoba, Spain; ^6^CREA–Research Centre for Cereal and Industrial Crops, Vercelli, Italy

**Keywords:** wheat, fusarium head blight, disease resistance, near isogenic lines, RNA-Seq, miRNA-Seq

## Abstract

Fusarium head blight (FHB), caused by the fungus *Fusarium graminearum*, represents one of the major wheat diseases worldwide, determining severe yield losses and reduction of grain quality due to the accumulation of mycotoxins. The molecular response associated with the wheat 2DL FHB resistance QTL was mined through a comprehensive transcriptomic analysis of the early response to *F. graminearum* infection, at 3 days post-inoculation, in spikelets and rachis. The analyses were conducted on two near isogenic lines (NILs) differing for the presence of the 2DL QTL (2-2618, resistant 2DL+ and 2-2890, susceptible null). The general response to fungal infection in terms of mRNAs accumulation trend was similar in both NILs, even though involving an higher number of DEGs in the susceptible NIL, and included down-regulation of the primary and energy metabolism, up-regulation of enzymes implicated in lignin and phenylpropanoid biosynthesis, activation of hormons biosynthesis and signal transduction pathways and genes involved in redox homeostasis and transcriptional regulation. The search for candidate genes with expression profiles associated with the 2DL QTL for FHB resistance led to the discovery of processes differentially modulated in the R and S NILs related to cell wall metabolism, sugar and JA signaling, signal reception and transduction, regulation of the redox status and transcription factors. Wheat FHB response-related miRNAs differentially regulated were also identified as putatively implicated in the superoxide dismutase activities and affecting genes regulating responses to biotic/abiotic stresses and auxin signaling. Altered gene expression was also observed for fungal non-codingRNAs. The putative targets of two of these were represented by the wheat gene *WIR1A*, involved in resistance response, and a gene encoding a jacalin-related lectin protein, which participate in biotic and abiotic stress response, supporting the presence of a cross-talk between the plant and the fungus.

## Introduction

Fusarium head blight (FHB), caused by the filamentous ascomycete *Fusarium graminearum* Schwabe [telomorph *Gibberella zeae* (Schwein.) Petch], represents one of the major wheat diseases worldwide, resulting in yield losses because of kernel shriveling, reduction of grain quality due to a selective loss of albumin and gluten proteins in the seed endosperm, and accumulation of toxic fungal secondary metabolites known as mycotoxins including deoxynivalenol (DON) and its dereivatives 3-acetyl-deoxynivalenol and 15-acetyl-deoxynivalenol (Brown et al., 2010).

The genetic bases of both FHB resistance type I (resistance to initial spikelet infection) and type II (resistance to the Transmission of FHB to other spikelets along the rachis), have been largely studied and a number of major and minor FHB resistance QTLs have been mapped in the wheat (*Triticum aestivum*) genome, supplying useful tools for breeding programs (Buerstmayr et al., 2009). Besides the identification of resistance sources, the understanding of the molecular mechanisms involved in the expression of FHB resistance plays also a relevant role in the development of new strategies to reduce the spread of the disease. To date, the gene responsible for only one FHB resistance QTL, *Fhb1* located on chromosome 3BS and conferring a durable broad-spectrum resistance, has been cloned and characterized. The type I *Fhb1* resistance, derived from the wheat cultivar (cv) Sumai 3, is due to the action of a pore-forming toxin-like (*PTF*) gene which encodes a chimeric lectin with two agglutinin domains and one ETX/MTX2 toxin domain (Rawat et al., 2016). Plant lectins are well-known to reversibly bind carbohydrates and to play a role in plant defense to insects, nematodes, bacteria, fungi, and viruses (Lannoo and Van Damme, 2014). ETX/MTX2 proteins are bacterial toxins that form channels in the host cell membrane leading to its death (Petit et al., 2001). Thus, it has been proposed that the PFT protein may participate in the recognition of fungal carbohydrates and cause toxicity to the fungus, arresting its growth by interacting with the fungal wall (Rawat et al., 2016). Candidate genes were also proposed for the *Fhb2* QTL, located on chromosome 6BS and also derived from Sumai 3, using an integrated large scale metabolo-transcriptomic study performed on recombinant inbred lines (RILs) carrying resistant (R) and susceptible (S) *Fhb2* alleles. These genes were represented by *loci* encoding a CoA ligase, callose synthase, basic Helix Loop Helix (bHLH) transcription factor (TF), glutathione S-transferase, ABC transporter-4, and cinnamyl alcohol dehydrogenase (Dhokane et al., 2016). This study also revealed a pathogen-related activation of phenylpropanoid and flavonoid pathways, receptor kinases, bHLH, WRKY, and MYB TFs and genes implicated in detoxification and signaling (Dhokane et al., 2016).

Other large scale analyses have been conducted to compare the response to FHB infection in different wheat genotypes. Erayman et al. (2015) performed a microarray analysis of the response to FHB (12 days post-inoculation—dpi), in the moderately susceptible (S) winter wheat cv Mizrak 98 and the S cv Gun 91 highlighting the implication of FHB-responsive pathogenesis related (PR) genes, TFs like WRKYs and bZIPs, and genes associated with the membrane activity. An alteration in the expression of genes implicated in carbohydrates and energy metabolism was also detected. The molecular analysis of the early (3 dpi) response to FHB of the highly R cv Nobeokabouzu-komugi characterized by low levels of mycotoxins in infected tissues, has highlighted the involvement of detoxification genes, like multidrug-resistance proteins, UDP-glycosyltransferase and ABC transporters, together with systemic defense-related genes (Kosaka et al., 2015b).

Several FHB R lines from a Chinese germplasm collection were shown to possess a resistance QTL on chromosome 2DL derived from the wheat line Wuhan-1 (Somers et al., 2003; He et al., 2014). A study conducted at early stages of infection of two NILs differing for presence/absence of the 2DL QTL (R 2DL+ 2-2618 and S null 2-2890) revealed that resistance occurs through the suppression of fungal growth at the point of initial infection in the R spikelets, thus reducing the progression of *F. graminearum* into the rachis, a mechanism compatible with a type II resistance (Long et al., 2015). Moreover, by comparing the transcriptional response of infected and control samples for both NILs, a general down-regulation of different classes of genes was detected with the most severe reduction observed for the FHB infected S null 2-2890. This down-regulation was mostly observed for genes involved in energy metabolism, transport, perception of environmental stimuli and adaptation, including auxin related genes, a brassinosteroid LRR receptor kinase-like gene, two genes associated with gibberellin response and one related to abscissic acid (ABA) response, suggesting that *F. graminearum* disrupts multiple phytohormone signaling pathways in spikes increasing 2-2890 susceptibility. Furthermore, eight resistance candidate genes, one of them predicted to have a close homolog on chromosome 2DL and encoding a methyltrasferase, were discovered (Long et al., 2015).

RNA-Seq is a Next Generation Sequencing (NGS)-based transcriptome analysis that has been utilized in many studies to describe the plant responses to pathogens (Bagnaresi et al., 2012; Kugler et al., 2013; Xiao et al., 2013; Zhu et al., 2013; Huang et al., 2016; Matic et al., 2016). Besides coding mRNAs, NGS-based analysis can also be employed to describe small non-coding RNAs and specifically microRNAs (miRNAs). miRNAs have been demonstrated to participate in many developmental-related processes like organ polarity, leaf growth, sex determination and sterility as well as in the response to abiotic and biotic stresses (Mallory and Vaucheret, 2006; Sunkar et al., 2012). miRNAs implicated in pathogen responses have been extensively reported in different plant species and were involved in the regulation of hormone signaling (Navarro et al., 2006; Wu et al., 2014; Baldrich et al., 2015), cell wall reinforcement (Gupta et al., 2012; Li et al., 2014), and disease resistance (Li et al., 2012, 2016; Boccara et al., 2014; Liu et al., 2014; Ouyang et al., 2014; de Vries et al., 2015; Yin et al., 2015). Despite the importance of the disease, to date no description of miRNAs implicated in wheat FHB disease response has been reported in literature.

In the present study, RNA-Seq and miRNA-Seq analyses were conducted at early stage of FHB infection (3 dpi) for spikelets and rachis of two bread wheat NILs, R 2DL+ 2-2618 and S null 2-2809, carrying respectively the R and S alleles for the FHB resistance QTL on chromosome arm 2DL. Metabolic pathways and miRNAs implicated in basal defense response were identified and, even if the precise location of the 2DL QTL is still unknown, several candidate genes whose functions could be associated with 2DL QTL-specific resistance were suggested. The analysis led to the discovery of several defense response strategies clearly differentiated in the two NILs. Two *loci* related to S null 2-2890 susceptibility, one corresponding to a miRNA, were identified. Additionally, our data suggested the presence of a cross-talk between the plant and the fungus.

## Materials and methods

### Plant materials and inoculation

The FHB R *T. aestivum* line 2-2618, carrying the 2DL FHB resistance QTL, and its S 2DL FHB QTL null NIL 2-2890 were selected for the NGS analyses. The NILs were generated from a cross between the R genotype HC374 and the S CDC Alsask (formerly called BW301), as described by Long et al. (2015). For quantitative real time PCRs (RT-qPCRs), one additional pair of NILs, segregating for the same QTL (2-2712−2DL+/2-2674—null) and derived from the same parental lines, was also used. Seeds of the four NILs were generously donated by Dr. Daryl Somers (Cereal Reserch Centre, Agriculture and Agri-Food Canada). Plants were grown in a controlled-environment cabinet with 16 h light at 20°C and 8 h dark at 16°C until mid-anthesis and then transferred to growth chambers. Infection was performed with the virulent *F. graminearum* isolate DAOM 180378 (Canadian Collection of Fungal Cultures, Agriculture and Agri-Food Canada, Ottawa, Canada). For the infection experiment, every developed spikelet of each head was inoculated (about 90–95% of each head) following the procedure reported by Long et al. (2015). All inoculated spikelets were harvested at 3 dpi. At the same time point, the rachis segments carrying inoculated spikelets were also collected. Mock controls for both tissues and lines were carried out. Three biological replicates, each consisting of spikelets and rachis from six wheat heads per sample, were performed (a total of 24 samples for each pair of NILs).

### RNA extraction, RNA-Seq, and smallRNA-Seq

Total RNAs were extracted using the TRIzol reagent (Life Technologies) followed by a treatment with DNase I (Ambion) according to provided instructions. For the RNA-Seq experiment, libraries were prepared using 1 μg of total RNA, purified by the RNeasy Mini Kit (QIAGEN), and the TruSeq RNA sample preparation kit (Illumina), according to manufacturers' instructions. Libraries were quantified through RT-qPCR, as recommended by the protocol, and single-end sequenced for 70 bases on an Illumina Genome Analyzer (GAIIx), multiplexing two samples per lane. miRNA libraries were prepared using 1 μg of unpurified total RNA and the TruSeq^(R)^ Small RNA kit (Illumina), following provided instructions. Libraries were 41 nt single-end sequenced on a GAIIx, multiplexing six samples per lane. The quality of RNAs and of both RNA-Seq and miRNA-Seq libraries was assayed on a 2100 Bioanalyzer (Agilent).

### Bioinformatic analysis of the RNA-Seq experiment

The fastQC application was utilized for filtering for low-quality reads and contaminants the raw fastQ files. Low quality reads (quality < or = 10 phred score) and contaminants were trimmed out by the Cutadapt software (Martin, 2011). Sequencing data have been deposited on the European Nucleotide Archive (ENA; https://www.ebi.ac.uk/ena; accession number: E-MTAB-6383). Filtered reads were mapped to wheat genome (Wheat Genome IWGSC release 2.25) with Tophat version 2.0.12 and Bowtie version 2.2.3.0 (Trapnell et al., 2012). HTSeq version 0.6.1 (Anders et al., 2014) was utilized for read count collection from BAM alignment files.

DEGs were called by the use of the DESeq2 package version 1.8 using parametric fit and betaPrior parameter set to True. DESeq2 implements differential expression analysis based on the Negative Binomial distribution (Love et al., 2014). The FDR [false discovery rate; (Benjamini and Hochberg, 1995) threshold for DEG calling was set to 0.001, while the fold change (FC) threshold was set to 2. Data visualization with Mapman (Thimm et al., 2004)] was performed by importing DESEq2-mormalized data into mapman application.

Wheat genes were assigned to mapman bins based on the available wheat mappings file: IWGSP_MIPSv2.2.txt (http://mapman.gabipd.org/web/guest/mapmanstore) upon parsing and adapting ID suffixes in mapman mapping file.

For gene onthology (GO) enrichments, the GOSeq bioconductor package (Robinson and Oshlack, 2010) was used as described in Baldoni et al. (2016). GOseq corrects for RNA length bias typical of RNA-Seq approaches (Oshlack and Wakefield, 2009). Gene IDs to GO mapping files were obtained from ensembl plants repository (Wheat IWGSC2.25 release). An FDR threshold of 0.01 was set for GO enrichment call.

Principal component analysis (PCA) on RNA-Seq data was performed on the covariance matrix for the DEGs in all the pair-wise comparisons using the “bpca” R package (Faria and Demétrio, 2013).

To identify modulation type (MT) groups, the common DEGs in different pair-wise comparisons were identified by a Venn diagram built up using all the eight comparisons and the Venn diagram tool on the UGent website (http://bioinformatics.psb.ugent.be/webtools/Venn/). The DEGs belonging to a specific group identified in the Venn diagram were then subgrouped on the basis of their differential expression in the corresponding comparisons. To verify if all the DEGs belonging to the same MT display the same expression pattern in the sample analyzed, MTs containing more than 10 *loci* was subjected to PCA analyses conducted on the expression of the DEGs in the three biological replicates for each sample, using the bpca R package.

### Bioinformatic analysis of the miRNA-Seq experiment

CLC Genomics workbench v8.0.2 (CLC bio, Denmark) was used for smallRNA analysis. The CLC Genomics Workbench approach to smallRNA analysis consists in counting the different types of smallRNAs in the data and in comparing them to microRNAs present in available databases or other smallRNAs. Adapters were trimmed under default parameters setting and only reads with lengths included between 18 and 30 nt were retained. Sequencing data have been deposited on the European Nucleotide Archive (ENA; https://www.ebi.ac.uk/ena; accession number: PRJEB24307). Selected 18–30 nt long reads were mapped on the *F. graminearum* genome, downloaded from https://genome.jgi.doe.gov/portal/pages/accessDenied.jsf?state=%27anonDownload%27, allowing 0 mismatches and setting length fraction and similarity fraction to value of 1. All the reads not mapped on the *F. graminearum* genome were then used to create a sample of wheat-related non-redundant reads with the corresponding count for subsequent analyses. The reads were annotated (default parameters allowing 0 mismatches) by the miRBase (release 21, taking as expression count the total number of reads mapping on the precursor, grouping by precursor option in the CLC software) dataset related to monocotyledons species (*T. aestivum, Triticum turgidum, Aegilops tauschii, Brachypodium distachyon, Hordeum vulgare, Elaeis guineensis, Festuca arundinacea, Oryza sativa, Sorghum bicolor, Saccharum officinarum, Zea mays*). Read counts of the annotated miRNAs were compared among several groups of samples and differential expression was detected by statistical analysis [Empirical analysis of differential gene expression (DGE), default parameters, FDR < 0.05, FC absolute value > or = 2]. Reads mapped on the *F. graminearum* genome were then analyzed following the same procedure and taking as reference the databases of milRNAs previously published by Chen et al. (2015) (*F. graminearum*), Chen et al. (2014) (*F. oxysporum*), Kang et al. (2013) (*T. ressei*), and Zhou et al. (2012) (*S. sclerotiorum*). Read counts of the annotated milRNAs were compared among R 2DL+ 2-2618 vs. S null 2-2890 and differential expression was detected by statistical analysis as reported above. PCA analysis on miRNA annotated read counts was conducted using the statistical PCA tool in the CLC Genomic Workbench software. miRNA MTs were identified in the same way described for the RNA-Seq experiment.

### Statistical analyses

Variance analyses were performed using the Tukey's range test (default parameters) in the SYSTAT 12 software.

### Quantitative real time PCRs (RT-qPCR)

Total RNAs extracted from two pairs of NILs, 2-2618/2-2890 (the same used in the NGS experiments) and 2-2712/2-2674 were used for RT-qPCRs, conducting two technical replicates for each biological replicate for mRNAs and three technical replicates for each biological replicate for miRNAs. For RNA-Seq validation, primer pairs were designed using the integrated DNA technologies (IDT; https://eu.idtdna.com/scitools/Applications/RealTimePCR/) for the genes reported in Supplementary Table 1. cDNA synthesis of DNase-treated and cleaned total RNAs was carried out using the RETROscript® reverse transcription kit (Ambion), using 3 μg of each RNA into a 20-μl reaction volume with oligo(dT)18 primer, using manufacturer's protocol. cDNAs were diluted 30 times and 5 μl were employed for each reaction. The Brilliant SYBR® Green QPCR core reagent kit and the MJ Research PTC200 thermal Cycler with Chromo 4 detector were utilized to performed RT-qPCRs. The 2^−ΔΔCt^ method (Livak and Schmittgen, 2001) was used to calculate FC values, and the relative expression levels were normalized against three wheat reference genes [*AOx* (UniGene54952), *w-GAPDH* (Unigene479973), and *hn-RNP-Q* (Unigene126248); (Long et al., 2015)] as calculated by Vandesompele et al. (2002). For miRNAs the stem-loop RT-PCR method (Varkonyi-Gasic et al., 2007) was applied. Stem loop RT primers and forward primers for RT-qPCR amplifications were designed as described by Chen et al. (2005; Supplementary Table 1). For miR164, miR9774, Fox-milRNA-1, and fox-milRNA-7 the reverse universal primer was utilized, for miR9653b the reverse primer was represented by rev3628 (Varkonyi-Gasic et al., 2007), while for miR168 and fox-milRNA-2c-d-e specific reverse primers (Tae_miR168_REV and fox-milRNA2_REV, respectively) were designed (Supplementary Table 1). The *T. aestivum snoR10* (Ta-snoR10) was the endogenous reference. Retrotranscription was conducted by using 200 ng of each RNA sample and the SuperScript III enzyme (Invitrogen), following manufacturer's instruction. Sybr GreenER RT-qPCR SuperMix for ABI PRISM (Invitrogen) was utilized for RT-qPCRs, according to the provided protocol.

To test the expression level of miR9653a precursor, its putative target WIR1-like (CA673319) and the other WIR1 family members WIR1A, WIR1B, and WIR1C, two-step RT-qPCRs were carried out. cDNAs were synthesized through the Super Script II enzyme (Invitrogen), following manufacturer's instructions, and quantified by the use of the Qubit Fluorometer (Invitrogen). Primers were designed by the Primer3 software (http://simgene.com/Primer3; Supplementary Table 1). The housekeeping gene was represented by the *T. aestivum* Polyubiquitin (*WubiG*). Reactions were performed by using 1 ng of cDNA for each sample and the KAPA SYBR FAST ABI Prism RT-qPCR Kit, according to manufacturer's indications. Reactions were run on a PCR thermal cycler 7300 Real Time PCR System (Applied Biosystem) and the SDS7300 absolute quantification software (Applied Biosystem) was applied for transcriptional comparative analyses. Relative gene expression was calculated by the 2^−ΔΔCt^ method (Livak and Schmittgen, 2001) on the averages of the three biological replicates.

## Results

### Phenotypic evaluations

**Figure 1:**
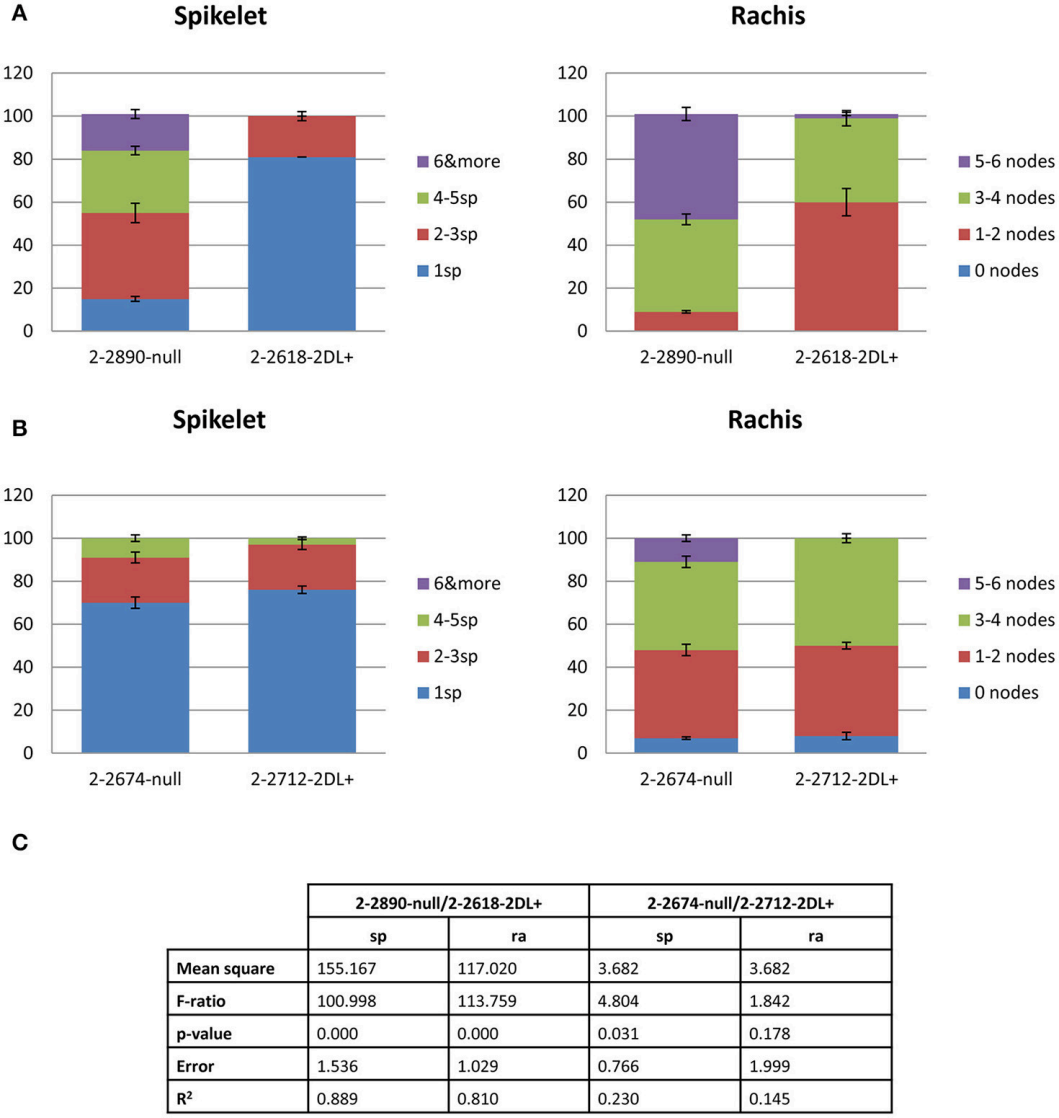
Bar plots representing the phenotypic data obtained by point inoculation of the two pairs of NILs: 2-2618 −2DL+/2-2890— null **(A)** and 2-2712 −2DL+/2-2674—null **(B)**. The percentages of plants showing infected spikelets and rachis internodes at 8 days post-inoculation (dpi) are reported. Each genotype is indicated and color legend refers to the number of infected spikelets and rachis. Error bars represent standard deviation. **(C)** Report of variance analyses conducted on phenotypic data. Sp, spikelets; ra, rachis.

Two pairs of NILs 2-2618 (2DL+)/2-2890 (2DL null) and 2-2712 (2DL+)/2-2674 (2DL null) were evaluated for visible FHB symptoms at 8 dpi. 80% of infected 2-2618 (R 2DL+) plants showed only one infected spikelet and 60% displayed only 1–2 infected nodes, while the corresponding S null 2-2890 showed a much higher spread of infection with about 20% of plants showing more than 6 infected spikelets and 50% of plants carrying 5–6 infected nodes (Figure 1A). A similar phenotype was scored for the other two NILs, 2-2712 (2DL+) and 2-2674 (null), with however a slightly lower infection level observed for the null line (Figure 1B). Variance analyses revealed significant differences in amount of infected tissues among the two genotypes 2-2618 (2DL+)/2-2890 (2DL null) (Figure 1C), while no significant differences were detected for the pair of NILs 2-2712 (2DL+)/2-2674 (2DL null; Figure 1C). These data suggest some interaction between the resistance QTL and other genes present in the 2-2674 genetic background and reflect previous observations made by Long et al. (2015) as in the S null 2-2890 the spreading of the infection occurred at greater extent compared to the R 2DL+ 2-2618, a finding in agreement with a type II resistance conferred by the 2DL FHB QTL.

### Overview of the RNA sequencing analyses

The sequencing of mRNAome and smallRNAome were assessed at an early stage of infection (3 dpi) on mock and infected spikelets and rachis of both NILs, R 2DL+ 2-2618 and S null 2-2890. Three biological replicates were performed for each condition, for a total of 24 samples.

#### RNA-Seq

For each sample the RNA-Seq analysis yielded from 6,043,327 to 27,170,339 filtered reads, and all of them were mapped to the Wheat Genome database IWGSC release 2.25 (https://www.wheatgenome.org/) and to the *F. graminearum* (Fg) genome (https://genome.jgi.doe.gov/portal/pages/accessDenied.jsf?state=%27anonDownload%27) (Supplementary Table 2). As expected, for all mock samples reads mapping to the fungus genome were almost absent (mean value = 0.09%), while Fg specific reads were present in infected samples (mean value = 13.71%; Supplementary Table 2). However, Fg reads were more abundant in the S null 2-2890 related samples (ranging from 18.39 to 36.06% for infected spikelets and from 11.39 to 29.45% for infected rachis), than in the R 2DL+ 2-2618 (from 6.13 to 7.25% for infected spikelets and from 3.37 to 4.17% for infected rachis; Supplementary Table 2). This finding confirms that the FHB colonization is reduced in the R genotype with respect to the S one, particularly for rachis. The analysis of variance, performed on the percentages of reads mapped on the *F. graminearum* genome in each sample, confirmed a significant difference among samples (*r*^2^ = 0.926), mainly due to treatment and genotype (Supplementary Table 3A). Correlation analyses between biological replicates from the same samples carried always Pearson coefficients values above 0.9, indicating a good level of reproducibility (Supplementary Table 4).

**Figure 2:**
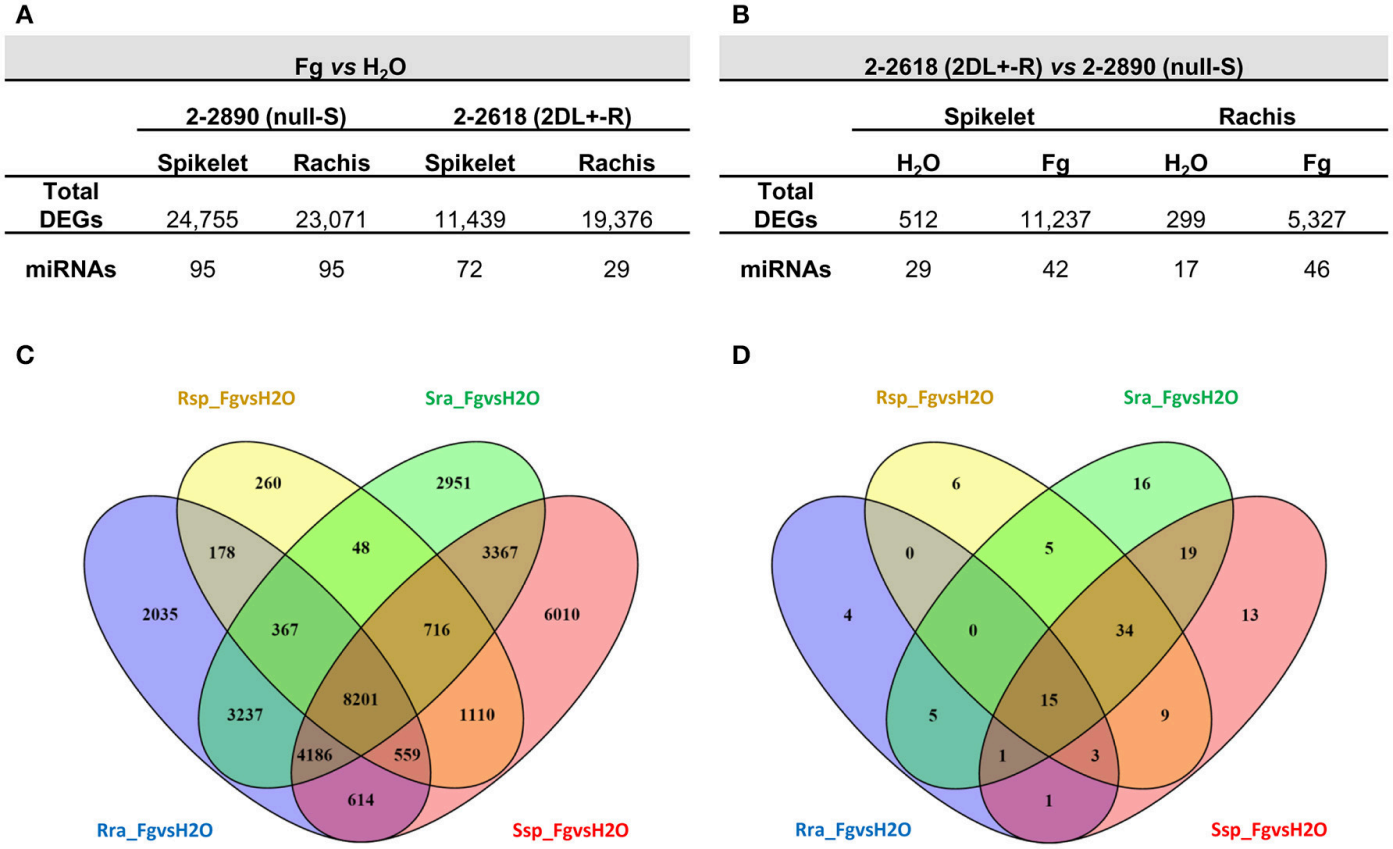
Numbers of differentially expressed genes (DEGs) identified in the infected vs. mock (Fg vs. H_2_O) **(A)** and resistant 2DL+ 2-2618 vs. susceptible null 2-2890 **(B)** comparisons for RNA-Seq and miRNA-Seq experiments. Venn diagrams of RNA-Seq DEGs **(C)** and miRNA-Seq DEGs **(D)** from all the Fg vs. H_2_O comparisons. S, susceptible null genotype 2-2890; R, resistant 2DL+ genotype 2-2816; sp, spikelet; ra, rachis; Fg, *F. graminearum* infected sample; H_2_O, mock control sample.

The following eight pair-wise comparisons were carried out to find genes implicated in FHB basal response and candidate genes for the 2DL FHB resistance QTL: infected by *F. graminearum* (Fg) vs. mock (H_2_O) samples, considering the same genotype and tissue (Fg vs. H_2_O_R spikelet; Fg vs. H_2_O_R rachis; Fg vs. H_2_O_S spikelet; Fg vs. H_2_O_S rachis), and of R vs. S samples, considering the same treatment and tissue (R vs. S_infected spikelet; R vs. S_infected rachis; R vs. S_mock spikelet; R vs. S_mock rachis). The highest number of DEGs in the comparisons between infected tissues and controls was observed for spikelets and rachis of the S null 2-2890 (24,755 and 23,071, respectively), while a substantial lower number of DEGs was detected for the R 2DL+ 2-2618 spikelets (11,439—Figure 2A), whereas the rachis from R 2DL+ 2-2618 had 19,376 DEGs. The successful establishment of the infection in the S null 2-2890 genotype in terms of number of cells targeted by FHB infection could result in a higher amount of DEGs. When the two genotypes were compared (R vs. S comparisons), the highest number of DEGs was highlighted for infected spikelets (11,237) followed by infected rachis (5,327—Figure 2B). A steady-state difference in gene expression between the two NILs in both mock tissues was also observed with the number of DEGs higher in the spikelets (512) than in the rachis (299—Figure 2B). A substantial number of DEGs were commonly modulated by infection in both genotypes: a total of 8,201 DEGs were in common among the four Fg vs. H_2_O comparisons, 7,826 were modulated in both S and R rachis, while 2,385 were in common between R and S spikelets (Figure 2C). However, a genotype-specific differential expression, after infection, was also present in the two tissues: 260, 2,035, and 178 DEGs were present in Fs vs. H_2_O comparisons for R spikelet, R rachis, and both R tissues, respectively; while 6,010, 2,951 and 3,367 were present in Fs vs. H_2_O comparisons for S spikelet, S rachis and both S tissues, respectively (Figure 2C).

**Figure 3:**
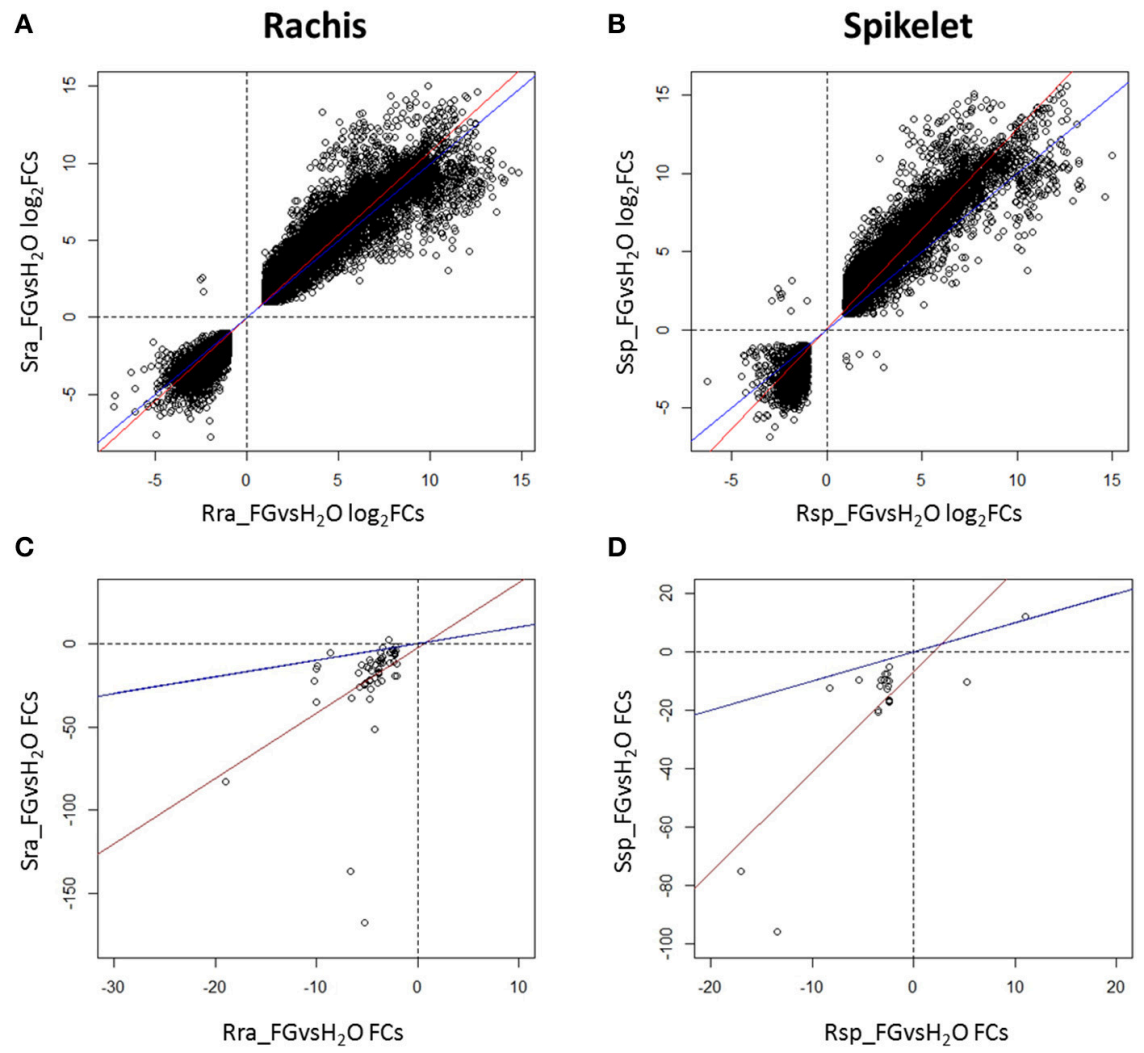
Dispersion graphs representing the differences in log_2_Fold Changes (log_2_FCs) of RNA-Seq DEGs identified in the inoculated vs. mock samples comparisons in the susceptible null 2-2890 (*y* axis) toward the resistant 2DL+ 2-2618 (*x* axis) in rachis **(A)** and spikelets **(B)**. Dispersion graphs representing the differences in FCs of miRNA-Seq DEGs identified in the inoculated vs. mock samples comparisons in the susceptible null 2-2890 (*y* axis) toward the resistant 2DL+ 2-2618 (*x* axis) in rachis **(C)** and spikelets **(D)**. Only common DEGs were considered for the four comparisons. Blue lines indicate the diagonal of the graph, while the red lines represent the actual correlation. S, susceptible null genotype 2-2890; R, resistant 2DL+ genotype 2-2816; sp, spikelet; ra, rachis; Fg, *F. graminearum* infected sample; H_2_O, mock control sample.

When the log_2_FCs of the DEGs detected in Fg vs. H_2_O in the S null 2-2890 were plotted against log_2_FCs in the R 2DL+ 2-2618 (only common DEGs were used), almost all DEGs showed the same trend of modulation in both NILs, although, the slope of the correlation curve indicates a slightly higher gene induction/repression in the S null genotype in both tissues, and particularly in spikelet (Figures 3A,B). The similarity between the responses of the two NILs was also confirmed by a principal component analysis (PCA), revealing that 97% of variance is explained by treatment and tissue, regardless to the genotype (Supplementary Figure 1). The same modulation of a common set of DEGs in R and S genotypes after infection likely indicate a common basal defense response displayed by the two NILs.

#### miRNA-Seq

The small RNA (sRNA)-Seq analysis generated 120,477,327 raw reads with an average of 5,019,888 reads per sample. The details are reported in Supplementary Table 2 where the numbers and the percentages of reads obtained after adapter trimming and size selection (18–30 nt) are also indicated. The percentage of reads included in the 18–30 nt range was generally lower in infected samples compared to the mock ones; additionally, a more relevant reduction of reads number was observed in the S null infected 2-2890 genotype with respect to the R 2DL+ infected 2-2618, and in spikelets, representing the point of infection, with respect to rachis (Supplementary Table 2). Variance analyses, conducted on the percentages of 18–30 nt reads in each sample, revealed significant differences (*R*^2^ = 0.993) of read length distribution in the 24 samples, primarily due to treatment, followed by genotype, treatment x genotype interaction, and tissue (Supplementary Table 3B). It is evident how *F. graminearum* infection modified the distribution of read length, increasing particularly the portion of short reads (lower than 18 nt) in both NILs (Supplementary Figure 2). The effect is more consistent in spikelets compared to rachis, and in the S null 2-2890 compared to the R 2DL+ 2-2618 (Supplementary Figure 2); for S null 2-2890, as mentioned above, the lack of the resistance 2DL QTL may involve an amplified defense response, given the more consistent spreading of the pathogen (Long et al., 2015). Nonetheless, further analyses are needed to better understand if these <18 nt RNAs represent only degraded fragments or sRNAs with a role in the wheat defense response to FHB infection.

All mock samples showed very low percentages (from 1.12 to 4.41%) of reads mapped on the *F. graminearum* genome (Supplementary Table 2), while in infected samples the percentages of reads assigned to the fungus were higher in the samples from S null 2-2890 (from 24.7 to 27.56% for infected spikelets and from 15.17 to 25.39% for infected rachis) than in the corresponding samples of R 2DL+ 2-2618 (from 9.11 to 15.37% for infected spikelets and from 7.18 to 8.43% for infected rachis; Supplementary Table 2). These numbers further confirm a lower fungal growth in R tissues with respect to the S ones, particularly for rachis, as already noticed with the RNA-Seq data. Variance analysis, performed on the percentages of reads mapped on the *F. graminearum* genome in each sample, gave similar results as for the RNA-Seq experiment with a significant difference among samples (*R*^2^ = 0.954), mainly due to treatment and genotype (Supplementary Table 3C).

Pearson correlation coefficient calculated with all the annotated miRNAs read counts between the two NILs was 0.98 between mock samples for both spikelets and rachis, while when infected samples were compared the coefficients between R and S NILs were 0.96 for spikelets and 0.91 for rachis. Comparison of Fg vs. mock samples yielded coefficients of 0.80 and 0.66 in the S line, and 0.83 and 0.95 in the R line for spikelets and rachis, respectively. This result indicates that infected rachis is more similar to the mock in the R 2DL+ 2-2618 than in the S null 2-2890. When the annotated read counts were subjected to PCA, the R 2DL+ 2-2618 infected rachis samples were separated from the other infected samples (Supplementary Figure 3). The whole picture obtained from this first level of analysis supports a general reduction of *F. graminearum* growth in the R 2DL+ 2-2816, when compared to the S null 2-2890, a finding in agreement with both RNA-Seq data and phenotypic evaluations (Figure 1) and coherent with the type II resistance described for the 2DL FHB resistance QTL from Wuhan-1 (Suzuki et al., 2012; Long et al., 2015). Type II resistance limits the spread of the disease along the spike and therefore reduces the extent of the miRNAome (and mRNAome) modulation in the rachis.

In the Fg vs. H_2_O comparisons, the most consistent regulation was observed in the S null 2-2890 where the highest number of differentially expressed annotated miRNAs was observed (95 in both tissues; Figure 2A). The lowest amount was instead detected for R 2-2618 rachis (29 differentially expressed miRNAs; Figure 2A), consistent with the fact that this tissue is the least responsive to the FHB attack, a further confirmation of a type II resistance associated with the 2DL FHB resistance QTL (Long et al., 2015). Moreover, in R vs. S comparisons the highest number of differentially expressed miRNAs was recorded for infected tissues (42 and 46 differentially expressed miRNAs in infected spikelets and rachis, respectively; Figure 2B). A steady-state expression difference between the two NILs was also observed for miRNAs and, like RNA-Seq, it was higher in mock spikelet (29 differentially expressed miRNAs) vs. mock rachis (17 differentially expressed miRNAs; Figure 2B).

Among the 106 miRNAs modulated during infection in spikelets, 61 (57.5%) were modulated in both NILs, while in rachis only 21 miRNAs, out of 103 miRNAs modulated by infection, were in common between the two genotypes (20.4%; Figure 2D). Like the RNA-Seq experiment, a genotype-specific modulation was discovered also for Fg vs. H_2_O differentially expressed miRNAs: 6 and 4 miRNAs were detected for R spikelet and R rachis, respectively; while 13, 16, and 19 miRNAs were found for S spikelet, S rachis, and both S tissues, respectively (Figure 2D).

With the exception of one differentially expressed miRNA in spikelet, all the differentially expressed miRNAs commonly modulated by infection in the two NILs showed the same regulation in both genotypes, at a higher level for the S null 2-2890 with respect to its R NIL, with the majority being down-regulated (Figures 3C,D).

In summary, both RNA-Seq and miRNA-Seq analyses highlighted a common FHB basal defense response for the two NILs that is however amplified in the S null 2-2890 for which the lack of the 2DL QTL and the consequent higher growth of the pathogen in the host tissues would imply an amplified transcriptional response.

### Analysis of differentially expressed genes (DEGs) and differentially expressed miRNAs related to FHB infection

**Figure 4:**
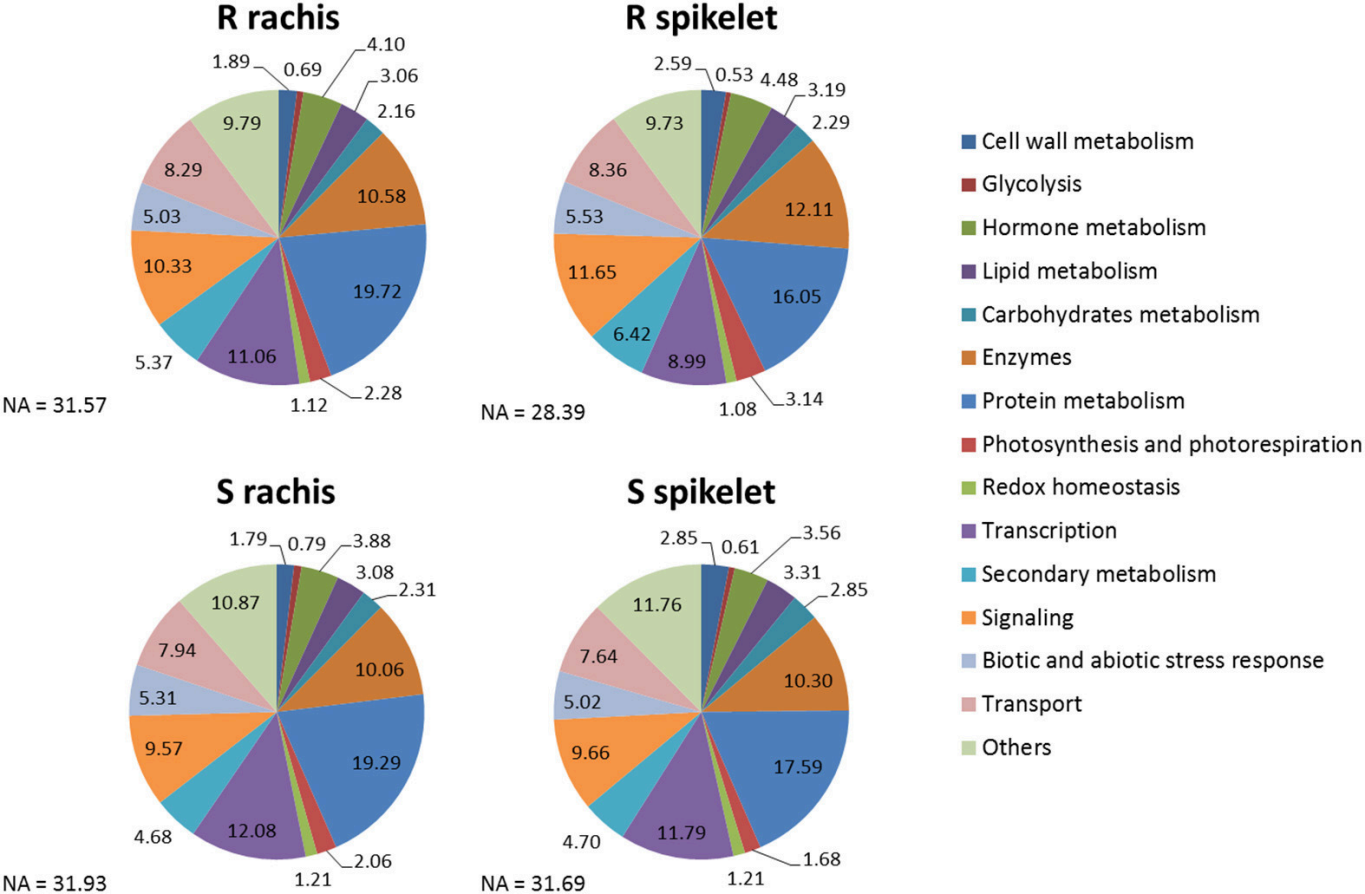
Pie charts representing the parcentages of DEGs, for each Fg vs. H_2_O comparison, belonging to the main MapMan functional cathegories. Each functional class is reported in the color legend. The genotype and tissue for each Fg vs. H_2_O comparison is indicated. S, susceptible null 2-2890; R, resistant 2DL+ 2-2618; NA, not assigned.

**Figure 5:**
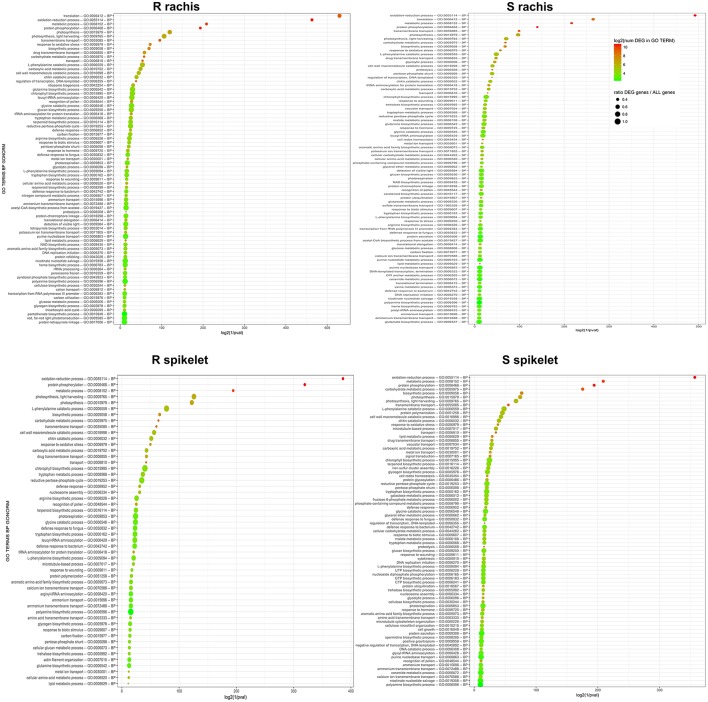
Plots representing the first 100 enriched GO terms, belonging to the biological processes class, detected by goseq in each Fg vs. H_2_O comparison. The genotype and tissue of each comparison are reported. log_2_(1\GO term enrichment *p*-values–*p*val) are indicated on X axis. The color of each circle represents the number of DEGs identified for each GO term, while the dimension corresponds to the ratio between the number of DEGs detected and the total number of genes included in each GO term. S, susceptible null genotype 2-2890; R, resistant 2DL+ genotype 2-2816; BP, biological process.

The pie charts in Figure 4 indicate the percentages of DEGs belonging to the most interesting MapMan functional cathegories identified for each Fg vs. H_2_O comparison, while the plots in Figure 5 show the first 100 enriched GO terms, belonging to the biological processes class, detected in each Fg vs. H_2_O comparison. The overall picture rising from both analyses further confirms that the two NILs display similar responsive strategies toward FHB, modulating the same biological processes, with the S null 2-2890 activating a higher response in terms of modulation intensity and of number of genes activated or repressed during infection.

Several DEGs and differentially expressed miRNAs identified were implicated in processes related to plant responses to pathogens like “defense response,” “redox homeostasis,” “hormone metabolism and signaling,” “cell wall metabolism,” “signal transduction,” and “regulation of transcription.” Other biological processes containing a consistent amount of DEGs were implicated in “carbohydrate metabolism,” “protein metabolism,” “transport,” and “detoxification.” Furthermore, each DEG was classified into a specific modulation type (MT) on the base of its differential expression across the comparisons, similarly to what was proposed by Long et al. (2015) and briefly described in Materials and methods. Tables 1, 2 report a summary of the MTs detected and the corresponding modulation in the different comparisons for RNA-Seq and miRNA-Seq experiments, respectively. A total of 385 different MTs were detected for the RNA-Seq experiment (Table 1 and Supplementary Data Sheet 1), while 65 MTs were identified in the miRNA-Seq analysis (Table 2 and Supplementary Data Sheet 2). This analysis allowed the identification of genes modulated by infection in both genotypes (e.g., MTs 1, 4, 7, 10, 23, and 36; Table 1 and Supplementary Data Sheet 1-Sheet 1), genes modulated by infection only in S null 2-2890 (e.g., MTs 2, 3, 14, 15, 21, and 24; Table 1 and Supplementary Data Sheet 1-Sheet 1) and genes which expression was alterated by infection specifically in the R 2DL+ 2-2618 (e.g., MTs 100, 116, 132, 152, 173, 208, 280, 284, and 288; Table 1 and Supplementary Data Sheet 1-Sheet 1). Differential expression among the two genotypes, regardless of treatment, was also highlighted (e.g., MTs 63, 94, 126, 140, 151, 172, 194, 202, 210, 219, 309, 323, and 350; Table 1 and Supplementary Data Sheet 1-Sheet 1) and *loci* down-regulated by infection in the R 2DL+ 2-2618 NIL while induced in the S null 2-2890 were identified and belonged to MTs 198 and 264 (Table 1 and Supplementary Data Sheet 1-Sheet 1).

**Table 1:** Modulation types (MTs) of all RNA-Seq DEGs identified in the eight pair-wise comparisons.

**MT**	**Fg vs. H**_**2**_**O**				**R vs. S**				**N. of DEGs**
	**R**		**S**		**sp**		**ra**		
	**sp**	**ra**	**sp**	**ra**	**Fg**	**H2O**	**Fg**	**H2O**	
1									2779
2									2177
3									2091
4									1930
5									1901
6									1741
7									1601
8									1503
9									1279
10									1102
11									997
12									970
13									945
14									916
15									892
16									817
17									780
18									775
19									576
20									566
21									484
22									450
23									446
24									407
25									376
26									336
27									234
28									197
29									193
30									180
31									179
32									154
33									154
34									147
35									140
36									139
37									131
38									127
39									125
40									115
41									113
42									108
43									107
44									107
45									106
46									98
47									95
48									95
49									79
50									78
51									69
52									65
53									63
54									61
55									60
56									60
57									51
58									50
59									49
60									45
61									45
62									41
63									39
64									38
65									37
66									35
67									34
68									34
69									33
70									33
71									33
72									31
73									27
74									27
75									27
76									26
77									24
78									23
79									22
80									22
81									22
82									21
83									20
84									20
85									16
86									16
87									16
88									16
89									16
90									14
91									14
92									14
93									14
94									14
95									13
96									13
97									13
98									13
99									13
100									13
101									13
102									13
103									12
104									11
105									11
106									10
107									10
108									10
109									10
110									10
111									10
112									10
113									9
114									8
115									8
116									8
117									8
118									8
119									8
120									8
121									8
122									8
123									8
124									7
125									7
126									7
127									7
128									7
129									7
130									6
131									6
132									6
133									6
134									6
135									6
136									6
137									6
138									6
139									6
140									5
141									5
142									5
143									5
144									5
145									5
146									5
147									4
148									4
149									4
150									4
151									4
152									4
153									4
154									4
155									4
156									4
157									4
158									4
159									4
160									4
161									4
162									4
163									4
164									4
165									4
166									3
167									3
168									3
169									3
170									3
171									3
172									3
173									3
174									3
175									3
176									3
177									3
178									3
179									3
180									3
181									3
182									3
183									3
184									3
185									3
186									3
187									3
188									3
189									3
190									3
191									3
192									3
193									3
194									3
195									3
196									3
197									2
198									2
199									2
200									2
201									2
202									2
203									2
204									2
205									2
206									2
207									2
208									2
209									2
210									2
211									2
212									2
213									2
214									2
215									2
216									2
217									2
218									2
219									2
220									2
221									2
222									2
223									2
224									2
225									2
226									2
227									2
228									2
229									2
230									2
231									2
232									2
233									2
234									2
235									2
236									2
237									2
238									2
239									2
240									2
241									2
242									2
243									2
244									2
245									2
246									2
247									2
248									2
249									2
250									2
251									2
252									2
253									2
254									2
255									2
256									2
257									2
258									1
259									1
260									1
261									1
262									1
263									1
264									1
265									1
266									1
267									1
268									1
269									1
270									1
271									1
272									1
273									1
274									1
275									1
276									1
277									1
278									1
279									1
280									1
281									1
282									1
283									1
284									1
285									1
286									1
287									1
288									1
289									1
290									1
291									1
292									1
293									1
294									1
295									1
296									1
297									1
298									1
299									1
300									1
301									1
302									1
303									1
304									1
305									1
306									1
307									1
308									1
309									1
310									1
311									1
312									1
313									1
314									1
315									1
316									1
317									1
318									1
319									1
320									1
321									1
322									1
323									1
324									1
325									1
326									1
327									1
328									1
329									1
330									1
331									1
332									1
333									1
334									1
335									1
336									1
337									1
338									1
339									1
340									1
341									1
342									1
343									1
344									1
345									1
346									1
347									1
348									1
349									1
350									1
351									1
352									1
353									1
354									1
355									1
356									1
357									1
358									1
359									1
360									1
361									1
362									1
363									1
364									1
365									1
366									1
367									1
368									1
369									1
370									1
371									1
372									1
373									1
374									1
375									1
376									1
377									1
378									1
379									1
380									1
380									1
381									1
382									1
383									1
384									1
385									1

*For each MT, the number of total DEGs is indicated. Green, up-regulation; red, down-regulation; S, susceptible null genotype 2-2890; R, resistant 2DL+ genotype 2-2816; sp, spikelet; ra, rachis; Fg, F. graminearum infected sample; H2O, mock control sample*.

**Table 2:** Modulation types (MTs) of the miRNA-Seq DEGs identified in the eight pair-wise comparisons.

**MT**	**Fg vs. H**_**2**_**O**				**R vs. S**				**n. of differentially expressed miRNAs**
	**R**		**S**		**sp**		**rac**		
	**sp**	**ra**	**sp**	**ra**	**Fg**	**H_2_O**	**Fg**	**H_2_O**	
1									11
2									10
3									9
4									8
5									7
6									5
7									4
8									4
9									4
10									3
11									3
12									2
13									2
14									2
15									2
16									2
17									2
18									2
19									2
20									2
21									2
22									2
23									2
24									2
25									2
26									2
27									2
28									1
29									1
30									1
31									1
32									1
33									1
34									1
35									1
36									1
37									1
38									1
39									1
40									1
41									1
42									1
43									1
44									1
45									1
46									1
47									1
48									1
49									1
50									1
51									1
52									1
53									1
54									1
55									1
56									1
57									1
58									1
59									1
60									1
61									1
62									1
63									1
64									1
65									1

*For each MT, the number of total differentially expressed miRNAs is indicated. Green, up-regulation; red, down-regulation. S, susceptible null genotype 2-2890; R, resistant 2DL+ genotype 2-2816; sp, spikelet; ra, rachis; Fg, F. graminearum infected sample; H2O, mock control sample*.

#### Carbohydrate metabolism

According to Long et al. (2015), the basal disease response associated with our genotypes goes through a down-regulation of the primary and energy metabolism as many genes implicated in these processes were modulated by infection in both NILs or in the S null 2-2890 only (Tables 1, 3 and Supplementary Data Sheet 1-Sheets 2, 3). In details, the presence of the fungus results in an inhibition of photosynthesis, photorespiration and starch biosynthesis, in favor of the activation of fermentation, tricarboxylic acid cycle, and secondary metabolism (Figure 4, Table 1 and Supplementary Data Sheet 1-Sheets 2, 3). For example, 111 and 55 down-regulated DEGs (MT10 and MT23, respectively) were implicated in photosynthesis and photorespiration, while 24 and 26 down-regulated DEGs (MT3 and MT20, respectively) were classified as implicated in major carbohydrate metabolism (Table 1 and Supplementary Data Sheet 1-Sheets 1–3). Conversely, the highest number of DEGs implicated in fermentation (8) was observed for MT4, while 191, 86, and 78 genes assigned to secondary metabolism belonged to MTs 1, 7, and 4, respectively (Table 1 and Supplementary Data Sheet 1-Sheets 1, 3).

**Table 3:** Expression patterns for the miRNAs and predicted target genes tested by RT-qPCR in the two pairs of NILs.

	**2-2890 (null)**				**2-2618 (2DL**+**)**			
	**Spikelet**		**Rachis**		**Spikelet**		**Rachis**	
	**Fg**	**H_2_O**	**Fg**	**H_2_O**	**Fg**	**H_2_O**	**Fg**	**H_2_O**
miR9774	+	+	+	+	+	+	+	+
pre-miR9653a	+	+	+	+	−	−	−	−
miR9666a	+	+	+	+	+	+	+	+
miR164	+	+	+	+	+	+	+	+
miR167c	+	+	+	+	+	+	+	+
miR168	+	+	+	+	+	+	+	+
miR9653b	+	+	+	+	+	+	+	+
miR398	+	+	+	+	+	+	+	+
fox-milRNA-2c-d-e	+	−	+	−	+	−	+	−
fox-milRNA7	+	−	+	−	+	−	+	−
Fg-milRNA-1	+	+	+	+	+	−	+	+
WIR-1-like	+	+	+	+	+	+	+	+
WIR1A	+	+	+	+	+	+	+	+
WIR1B	+	+	+	+	+	+	+	+
WIR1C	+	+	+	+	+	+	+	+
	**2-2674 (null)**				**2-2712 (2DL**+**)**			
	**Spikelet**		**Rachis**		**Spikelet**		**Rachis**	
	**Fg**	**H**_2_**O**	**Fg**	**H**_2_**O**	**Fg**	**H**_2_**O**	**Fg**	**H**_2_**O**
pre-miR9653a	−	−	−	−	−	−	−	−
miR398	+	+	+	+	+	+	+	+
fox-milRNA-2c-d-e	+	−	+	−	+	−	+	−
fox-milRNA7	+	−	+	−	+	−	+	−
Fg-milRNA-1	+	−	+	−	+	−	+	−
WIR-1-like	+	+	+	+	−	−	−	−
WIR1A	+	+	+	+	+	+	+	+
WIR1B	+	+	+	+	+	+	+	+
WIR1C	+	+	+	+	+	+	+	+

*Fg, infection by F. graminearum; H_2_O, mock; +, amplified; −, not amplified*.

#### Cell wall modification

Several cell wall-related responses were present in both, R and S NILs; indeed, a total of 209 *loci* implicated in phenylpropanoid biosynthesis (201 involved in lignin biosynthesis) were part of MT1, while 267 phenylpropanoid biosynthetic DEGs (265 corresponding to enzymes implicated in lignin biosynthesis) were present in MT4 (Supplementary Data Sheet 1-Sheet 2). Moreover, down-regulation after infection of pectin acetyl esterases occurred (e.g., 4 and 3 *loci* were present in MTs 3 and 19, respectively; Supplementary Data Sheet 1-Sheet 2) together with an over-expression of pectin methyl esterases (3, 4, and 4 in MTs 1, 2, 9, respectively; Supplementary Data Sheet 1-Sheet 2). Moreover, in all the Fg vs. H_2_O comparisons, activation of flavonoids, specifically chalcones, flavonols, dihydroflavonols, and anthocyanins biosynthetic pathways were recorded (see for example MT4; Supplementary Data Sheet 1-Sheet 2).

Two *loci* implicated in cell wall metabolism but not modulated by infection were differentially expressed between the two NILs. The first is represented by the Traes_3al_194ff20fa gene, encoding for cinnamyl-alcohol dehydrogenase, that was more transcribed in both R tissues (log_2_FCs ~ 2 in all the R vs. S comparisons), with respect to S, in both conditions (MT63; Table 1 and Supplementary Data Sheet 1-Sheets 1, 2). The second gene was Traes_2dl_0dffd3ce2, more expressed (log_2_FCs ~ 2) in R spikelets, with respect to the S one, in both conditions (MT94; Table 1 and Supplementary Data Sheet 1-Sheet 1), and corresponded to a putative alpha-L-fucosidase implicated in xyloglucan degradation, removing xylose from xyloglucan 1 precursor (Supplementary Data Sheet 1-Sheet 2).

The Traes_1bl_f026049bc *locus*, corresponding to an O-methyl transferase ZRP4 (Supplementary Data Sheet 1-Sheet 2), was induced by infection in the R genotype only (log_2_FCs = 3.64 and 5.95 for spikelet and rachis, respectively) and more expressed in R vs. S (log_2_FCs = 3.94) in infected spikelets (MT113; Table 1 and Supplementary Data Sheet 1-Sheet 1).

Several DEGs implicated in cell wall metabolism were repressed by infection in the R genotype and were down-regulated in the R vs. S at least in infected spikelet: Traes_2ds_7bfbc3007 and Traes_2as_f4e575606 (MT132), Traes_2ds_0eb3b8c4e and Traes_2bs_18ed569db (MT208) encoding cellulose synthases, while Traes_5bl_8512c24f7 corresponds to a beta-1,4-glucanase (MT173) (Table 1 and SupplementaryData Sheet 1-Sheets 1, 2). The miRNA-Seq experiment revealed that miR9653a was considerably more expressed in S null 2-2890 than in R 2DL+ 2-2618 (FC values from 116 to 1,019.5) in both infected and mock tissues (MT22; Supplementary Data Sheet 2-Sheet 1). Among the putative miR9653a targets, a gene homolog to the members of the WIR1 family (WIR1-like—CA673319) was detected. Even if this gene was not annotated on the wheat genome released used in this work, and thus it was not possible to find it among the DEGs in the RNA-Seq experiment, it belongs to a gene family well-known for being involved in cell wall reinforcement (Coram et al., 2010).

#### Sugar signaling

Among the DEGs showing differential expression in the two genotypes regardless of treatment, Traes_3as_c082cdcfe was not responsive to infection but more transcribed in the R NIL, with respect to the S one (MT63; log_2_FCs from 1.04 to 2.13; Table 1 and Supplementary Data Sheet 1-Sheet 1), and encoded for a putative plastidic glucose transporter implicated in response to trehalose *stimulus* (Supplementary Data Sheet 1-Sheet 2). The same MT63 was also detected for genes encoding respectively for a SNL TF (Traes_3as_acac429eb), a MYB domain-containing TF (Traes_3as_6626d4a69) and a WD40 repeat protein (Traes_3as_a3ce60e4f; Supplementary Data Sheet 1-Sheets 1, 2). These are all gene functions involved in responses to trehalose stimulus, suggesting that the threhalose signaling pathway might regulate FHB response in the R 2DL+ 2-2618. Furthermore, Traes_3dl_8c6d663c5, repressed by infection in R tissues and S rachis and less expressed (log_2_FC = −1.04) in R infected spikelets compared to the S one (MT119; Table 1 and Supplementary Data Sheet 1-Sheet 1), corresponded to a Protein Phosphatase 2C (PP2C) homolog to the *A. thaliana* ABI1 (Supplementary Data Sheet 1-Sheet 2), which negatively regulate the energy sensor SnRK1 (Rodrigues et al., 2013).

An opposite regulation by infection in the two genotypes was recorded for Traes_6as_ac24f31a6 which was repressed by infection in R spikelets and induced by infection in S spikelets, while was more expressed (log_2_FC = 3.05) in R vs. S in mock treated spikelets (MT377; Table 1 and Supplementary Data Sheet 1-Sheet 1). This gene encodes for a β-fructofuranosidase 1- invertase 1- precursor, implicated in sucrose catabolism (Supplementary Data Sheet 1-Sheet 2).

#### Hormone metabolism and signaling

A general activation of hormone metabolism and signaling was detected after infection in our experiment as a higher number of DEGs implicated in these pathways was present in MTs up-regulated after infection, compared to those down-regulated (Supplementary Data Sheet 1-Sheets 2, 3). The activation of salicylic acid (SA), jasmonic acid (JA), ethylene (ET), and ABA biosynthesis and signal transduction pathways was recorded in all the Fg vs. H_2_O comparisons. For example, out of 135 MT1 DEGs involved in hormone metabolism, 102 were implicated in ABA metabolism, 63 in JA metabolism, 6 in SA metabolism, and 4 in ET metabolism; of the 63 MT4 hormone metabolism-related DEGs, 18 were implicated in SA metabolism, 6 in JA metabolism, and 19 in ET metabolism; among the 55 MT7 DEGs involved in hormone metabolism, 8, 6, and 11 represented genes related to ET, JA, and SA metabolic pathways, respectively (Supplementary Data Sheet 1-Sheets 2, 3). Moreover, even genes involved in auxin metabolism were induced by infection in both genotypes, at higher level in S null 2-2890, as, for example, 31 MT1 DEGs were classified as acting in auxin metabolism and signaling (Supplementary Data Sheet 1-Sheets 1, 2). Repression of auxin response pathway enhances *A. thaliana* susceptibility to the necrotrophic *fungi P. cucumerina* and *B. cinerea* (Llorente et al., 2008). Further experiments are needed to better understand the contribution of this hormone to FHB response.

The amplified FHB response in the S null 2-2890 NIL was further demonstrated by some *loci* involved in JA metabolism and signaling belonging to MT106 and less expressed in all the R vs. S comparisons: Traes_6as_70b2f2ae4 encoded for the cytochrome P450 CYP74, involved in the JA biosynthetic pathway; while Traes_1ds_75af80583 and Traes_1bs_527ed00b9 represented two C2H2 zinc finger TFs (Table 1 and Supplementary Data Sheet 1-Sheets 1, 2), affecting JA- and ABA-mediated resistance to pathogens (Deb et al., 2016).

#### Detoxification

A class of enzymes implicated in *F. graminearum* response is represented by the UDP-glycosyltrasferases which participate in DON detoxification (Kugler et al., 2013; Schweiger et al., 2013; Li et al., 2015). Even in our case UDP-glycosyltrasferases might be implicated in basal response to FHB as several *loci* corresponding to this class of enzymes were induced by infection in both NILs, at higher level in S null 2-2890. For example, 72 UDP-glycosyltrasferases encoding genes belonged to MT1, 20 to MT4 and 28 to MT7 (Supplementary Data Sheet 1-Sheet 2). Mycotoxin detoxification is also related to the ABC transporter family (Kosaka et al., 2015a; Walter et al., 2015) which was found up-regulated after infection in both genotypes: 35 *loci* corresponding to ABC transporters belonged to MT1, 42 to MT4 and 50 to MT7 (Supplementary Data Sheet 1-Sheet 2).

The ABC transporter encoding *locus* Traes_3as_e18f065a3 was considered likely associated with the 2DL FHB resistance as it was more expressed in R vs. S in both tissues and treatments (log_2_FCs ranging from 1.86 to 2.49; MT63; Supplementary Data Sheet 1-Sheets 1, 2).

#### Defense response

As expected, numerous genes implicated in biotic stress response were up-regulated by infection in both NILs and tissues. Of these, a number of genes coding for PR proteins were present in MTs 1 (19 DEGs) and 4 (24 DEGs; Supplementary Data Sheet 1-Sheet 2). Moreover, several *loci* involved in signaling processes triggering plant defenses were found induced by infection. Among them, several genes encoded G proteins (30, 22, and 15 DEGs in MTs 1, 2, and 4, respectively), MAPKs (8, 9, 10, and 6 *loci* in MTs 1, 2, 4, and 7, respectively), lectins (25, 30, and 17 DEGs in MTs 1, 4, and 7, respectively)—a class of proteins recently demonstrated to be associated to the wheat FHB resistance gene *Fhb1* (Rawat et al., 2016), LRR receptor kinases (52, 56, and 41 DEGs in MTs 1, 4, and 7, respectively) or cell wall receptor kinases (29, 19, and 11 DEGs in MTs 1, 4, and 7, respectively) and different genes were implicated in Ca^2+^ signaling (Supplementary Data Sheet 1-Sheet 2).

A number of genes implicated in the signal transduction triggering plant defense responses were present in MTs associated with 2DL FHB resistance (Supplementary Data Sheet 1-Sheets 2, 3). Traes_2dl_7788247ee encodes a LRR transmembrane protein kinase (Supplementary Data Sheet 1-Sheet 2) belonging to a class of membrane receptors implicated in pathogens recognition (Monaghan and Zipfel, 2012). This DEG, displayed in the MT100, showed induction by infection in R tissues and higher expression (log_2_FCs = 1.18 and 1.81 for infected spikelet and rachis, respectively) in R 2DL+ 2-2618 compared to S null 2-2890 (Table 1 and Supplementary Data Sheet 1-Sheet 1). Other receptors displaying MT compatible with the involvement in the 2DL FHB resistance were represented by the MT113 Traes_3as_bc76a2e7e, a LRR kinase, Traes_3ds_072b70fd7, homolog to wheat LRK10-like kinase, and Traes_3ds_5870f8f48, homolog to thaumatin-like kinase, all induced by infection in both R tissues with a higher transcription in R vs. S comparison in infected spikelets (Table 1 and Supplementary Data Sheet 1-Sheets 1, 2). Additionally, the RLK receptor kinase encoding gene Traes_1as_581d331e0 was activated by infection in R spikelets only, thus resulting in higher (log_2_FC = 1.17) expression in R infected spikelets, with respect to the S (MT116; Table 1 and Supplementary Data Sheet 1-Sheets 1, 2). The same MT116 was associated with other *loci* encoding proteins implicated in plant disease resistance: Traes_7bl_22d90b6c6, corresponding to a NB-ARC domains-containing disease resistance protein, and a CC-NB-LRR protein encoded by the gene Traes_7al_278f88e19 (Supplementary Data Sheet 1-Sheets 1, 2).

Moreover, lower mRNA levels (log_2_FCs from −1.21 to −2) were detected in the R 2DL+ 2-2618 NIL, in comparison to the S null 2-2890, for Traes_2dl_7b0056729 (MT103), a gene encoding a serinc domain-containing protein implicated in sphingolipids biosynthesis (Table 1 and Supplementary Data Sheet 1-Sheets 1, 2).

In addition, Traes_7ds_6333b91f8 (MT288) and Traes_7as_95f4e4729 (MT370), both repressed by infection in R spikelets and in the R 2DL+ 2-2618 NIL in comparison to S null 2-2890, encoded for a peptidase S41 (Supplementary Data Sheet 1-Sheets 1, 2). This class of proteases is homologous to the proteases down-regulated in tobacco plants R to the tobacco mosaic virus during the infection (Jada et al., 2014), therefore, the inhibition of this gene after FHB infection in R 2DL+ 2-2618 might be associated with the 2DL QTL resistance.

The miRNA-Seq experiment revealed that two members of the miR9863 family, miR9863a, and miR9863b, were down-regulated by infection in S null 2-2890 rachis and spikelets and, to a less extent, in R spikelets, thus resulting in higher expression in R 2DL+ 2-2618 vs. S null 2-2890 infected tissues (MT5; Table 2 and Supplementary Data Sheet 2-Sheet 1).

#### Redox homeostasis

Several genes participating in the maintaining of cellular redox homeostasis were modulated by infection in both genotypes. As examples, 15 redox homeostasis DEGs belonged to MT1, 15 to MT4, 12 to MT7 and 20 to MT10 (Supplementary Data Sheet 1-Sheet 3) and might participate in FHB basal response.

Nevertheless, several genes involved in redox homeostasis-related processes were differentially regulated in the R and S NILs. Traes_2dl_03caa3b80, homolog to the *Arabidopsis* P-Type ATPase 2 (PAA2), showed a peculiar MT as being the only gene induced by infection in R spikelets (log_2_FC = 1.39) and more expressed in R vs. S in both tissues and conditions (log_2_FCs ranging from 5.57 to 8.29; MT280; Table 1 and Supplementary Data Sheet 1-Sheet 1). Moreover, the Cu-miRNAs miR398, which targets two Cu/Zn superoxide dismutases (CSDs), the cytosolic CSD1 and CSD2, was down-regulated by infection in S null 2-2890 tissues, in comparison to the R ones (FCs = −77.29 and −55 for spikelet and rachis, respectively; MT2 from miRNA-Seq experiment; Supplementary Data Sheet 2-Sheets 1, 2). Finally, we observed that two genes homolog to *Arabidopsis* CSD1, Traes_2ds_3c3a2a12a and Traes_2bs_6015bc7c6 (MT15), were induced by infection in S null 2-2890 (Supplementary Data Sheet 1-Sheets 1, 2).

#### Regulation of transcription

TFs were among the functional classes more extensively modulated by FHB infection (Figure 4; Supplementary Data Sheet 1-Sheet 3) and an up-regulation in the Fg vs. H_2_O comparisons was detected for several TF families known to be implicated in plant resistance response. As example, 29 and 10 *loci* encoding WRKY TFs (Satapathy et al., 2014) were present in MTs 1 and 7, respectively; 27 MT1 DEGs correspond to C2H2 zinc fingers (Kielbowicz-Matuk, 2012); 11 MT1 DEGs encode AP2s (Gutterson and Reuber, 2004); 20 MT1, 15 MT2, 10 MT4, and 9 MT7 DEGs are MYBs (Zhang et al., 2015); 29 MT1 and 12 MT4 correspond to NACs (Nuruzzaman et al., 2013; Supplementary Data Sheet 1-Sheet 2).

Among the genes differentially expressed between the two NILs, Traes_3as_6626d4a69 (a MYB domain-containing TF; MT63, Table 1 and Supplementary Data Sheet 1-Sheets 1, 2) and Traes_4bs_2ea81743e1 (a Chitin-inducible gibberellin-responsive GRAS TF; MT196, Supplementary Table 5 and Supplementary Data Sheet 1-Sheets 1, 2) were both more expressed in R than S infected spikelets (log_2_ FCs = 2.57 and 1 for the two loci, respectively).

miR164, which targets NAC TFs, was repressed by infection in both genotypes and tissues but at higher levels in S null 2-2890 (FC of about−12 in the S NIL and of −2.79 and −8.25 in the R genotype; MT6; Table 2 and Supplementary Data Sheet 2-Sheet 1). Furthermore, it was more expressed in R 2DL+ 2-2618 infected rachis samples compared to the S ones (FC = 5.36; Table 2 and Supplementary Data Sheet 2-Sheet 1).

miR319b and miR319d were identified as repressed after infection in both genotypes and tissues, at higher levels for S null 2-2890 (FCs = −24.17 and −17.12 for S spikelet and rachis, respectively and FCs = −5.25 and −2.41 for R spikelet and rachis, respectively; MT25), and more expressed in R vs. S in spikelets in both conditions and in infected rachis (FCs from 2.01 to 9.12; Table 2 and Supplementary Data Sheet 2-Sheet 1). These miRNAs belong to a family whose putative targets are represented by the wheat MYB3 TFs homologous to *A. thaliana AtMYB59*, which is induced in response to JA and ET and inhibits cell growth (De Paola et al., 2016).

### Analysis of *F. graminearum* miRNA-like RNAs

Fungi produce several ncRNAs which share many similarities with plant and animal miRNAs and are named miRNA-like RNAs (milRNAs) because some of them are produced by similar but not identical mechanisms as those for conventional miRNAs (Lee et al., 2010).

The 18–30 nt long reads from R 2DL+ 2-2618 and S null 2-2890 infected samples were mapped on the *F. graminearum* genome and then analyzed to detect the presence of milRNAs previously annotated in four fungal species: *F. graminearum* (Chen et al., 2015), *Fusarium oxysporum* (Chen et al., 2014), *Trichoderma ressei* (Kang et al., 2013), and *Sclerotinia sclerotiorum* (Zhou et al., 2012). Similarities were found only for milRNAs from the genus *Fusarium*, leading to the identification of 15 different groups (Supplementary Table 5). Most of the fungal milRNAs were present only in infected samples, although in some cases the corresponding reads were also detected in control samples even if just for a few of the isomiRs. This is probably due to an overlap between host and pathogen ncRNAs as mentioned before.

Interestingly, fox_miRNA_7, identified in infected samples but present at low levels also in mock samples, showed, as a putative target, the WIR1A encoding mRNA (CA698068). WIR1A belongs to same gene family as the putative target of miR9653a (the so called WIR1-like), which is consistently more expressed in S null 2-2890 than in R 2DL+ 2-2618. Taken together these results represent a further support to the possible relevant implication of the WIR gene family in wheat FHB resistance governed by the 2DL QTL. The role of fungal smallRNAs (sRNAs) as effectors to suppress host immunity is not new and was recently reported by Weiberg et al. (2013) for *Botrytis cinerea*. The authors demonstrated that sRNAs from *B. cinerea* can interfere with plant resistance by targeting MAPK1-2, the oxidative stress-related gene *PRXIIF*, coding for a peroxiredoxin, and a cell wall-associated kinase (WAK).

For fox-milRNA-2c-d-e, present only in infected samples, less reads were detected in R 2DL+ 2-2618 line, particularly in spikelets, compared to S null 2-2890, according to the greater fungal biomass expected in the S genotype. A rice Jacalin-like lectin domain containing protein encoding gene (TC379239) was detected as a putative target of this class of milRNA by PsRNATarget. The jacalin-related lectins participate in biotic and abiotic stress response in wheat (Song et al., 2014) and a lectin encoding gene was recently found to be associated with the *Fhb1* wheat QTL (Rawat et al., 2016), further supporting the presence of a cross-talk between the plant and the pathogen.

### Validation by quantitative real time PCR (RT-qPCR)

RNAs extracted from the same experimental setting as the RNA-Seq analysis and from another pair of NILs (2-2712/2-2674), differing for the presence of the 2DL QTL, were utilized for RT-qPCR validation.

**Figure 6:**
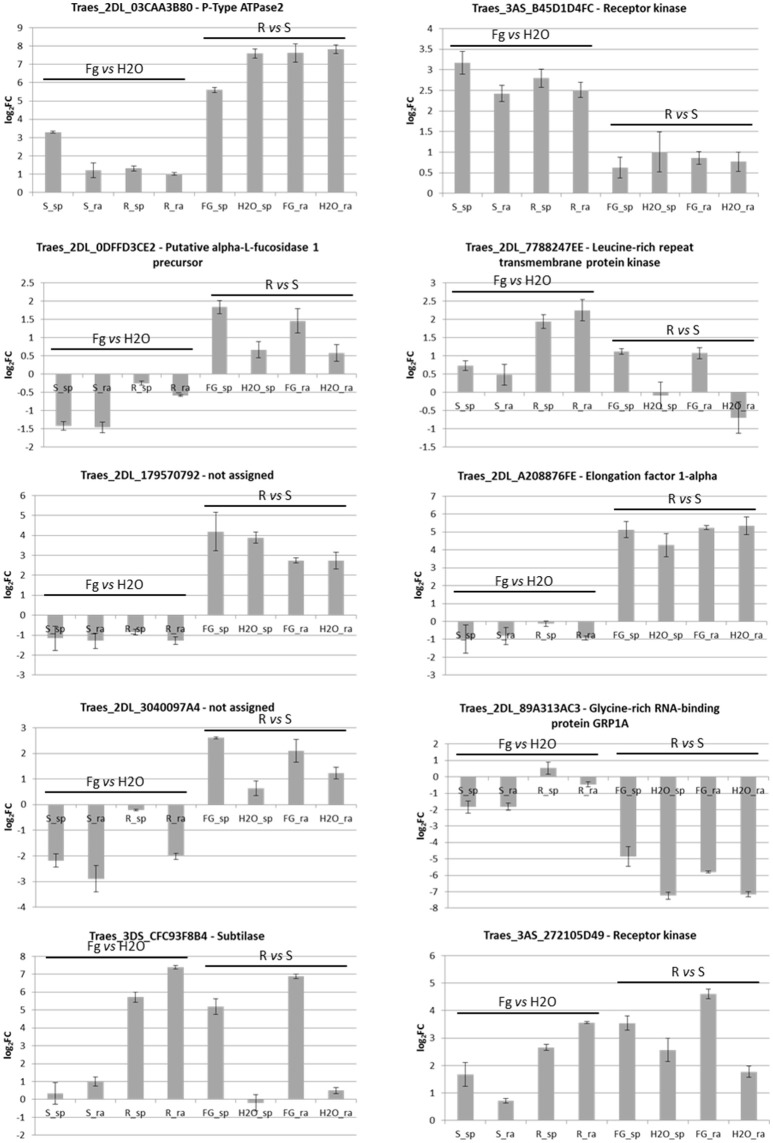
Bar plots representing RT-qPCR results for 10 selected DEGs identified in the RNA-Seq experiment and performed on RNAs from the pair of NILs R 2DL+ 2-2618/S null 2-2890. Each bar corresponds to the log_2_FC detected for the comparison indicated and expressed as average of three biological replicates. Standard deviations are indicated. sp, spikelet; rach, rachis; Fg, *F. graminearum* infected sample; H_2_O, mock control sample; R, resistant 2DL+ 2-2618; S, susceptible null 2-2890.

**Figure 7:**
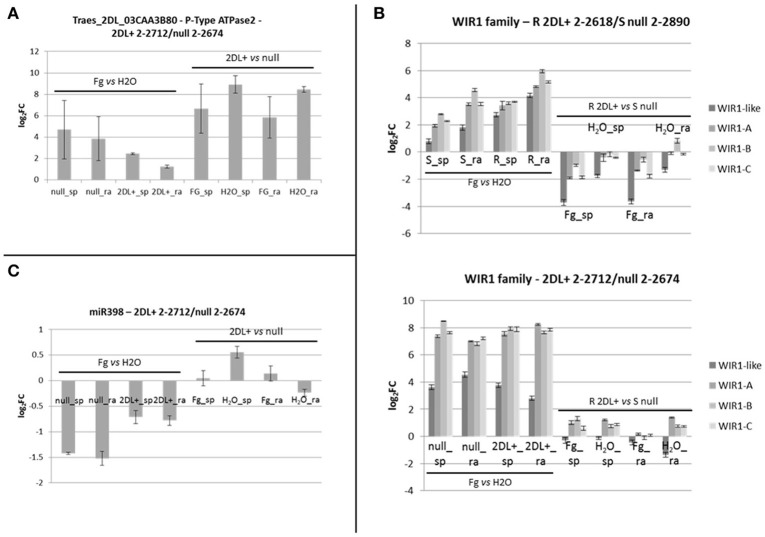
**(A)** Bar plots representing RT-qPCR results for Traes_2dl_03caa3b80 and performed on the pair of NILs 2DL+ 2-2712/null 2-2674. Each bar corresponds to the log_2_FC detected for the comparison indicated and expressed as average of three biological replicates. Standard deviations are indicated. **(B)** Bar plots representing RT-qPCR results for the members of the WIR1 gene family performed on the two pairs of NILs R 2DL+ 2-2618/S null 2-2890 and 2DL+ 2-2712/null 2-2674. Each bar corresponds to the log_2_FC detected for the comparison indicated and expressed as average of three biological replicates. Standard deviations are indicated. Each color corresponds to a specific gene as indicated in the color legend. **(C)** Bar plot representing RT-qPCR results for miR398 performed on the pair of NILs 2DL+ 2-2712/null 2-2674. Each bar corresponds to the log_2_FC detected for the comparison indicated and expressed as average of three biological replicates. Standard deviations are indicated. sp, spikelet; rach, rachis; Fg, *F. graminearum* infected sample; H_2_O, mock control sample; R, resistant 2DL+ 2-2618; S, susceptible null 2-2890.

From the RNA-Seq data analysis, 10 *loci* were selected for general validation in NILs 2-2618 and 2-2890: Traes_2dl_03caa3b80—P-Type ATPase (MT280), Traes_2dl_0dffd3ce2—alpha-L-fucosidase 1 precursor (MT94), Traes_2dl_a208876fe—elongation factor 1-alpha (MT94), Traes_2dl_7788247ee—LRR transmembrane protein kinase (MT100), Traes_2dl_179570792—function unknown (MT63), Traes_2dl_89a313ac3—glycine-rich RNA-binding protein GRP1A (MT139), Traes_2dl_3040097a4—function unknown (MT202), Traes_3as_b45d1d4fc—receptor kinase LRK10 (MT166), Traes_3ds_cfc93f8b4—subtilase (MT300), Traes_3as_272105d49—receptor kinase LRK10 (MT364; Supplementary Data Sheet 1-Sheet 1). A general agreement, in terms of up- and down-regulation in the eight pair-wise comparisons, between RNA-Seq and RT-pPCR results was found, particularly for the differential expression between genotypes (Figure 6). However, some differences were highlighted, probably due to the different sensibility of the two methods. For example, Traes_2dl_03caa3b80 was induced by infection only in the R spikelets in the RNA-Seq experiment (Supplementary Data Sheet 1-Sheet 1), while by RT-qPCR higher expression was detected also in infected R rachis in comparison to the mock ones, even if at a lower level with respect to the spikelets. The differential expression among 2DL+ and null genotypes for Traes_2dl_03caa3b80 was also detected for the additional NIL pair 2-2712/2-2674 (Figure 7), suggesting that this *locus* might be related to the presence of the 2DL FHB resistance QTL.

To validate miRNA-Seq data, eight miRNAs were selected for RT-qPCRs performed on NILs 2-2618/2-2890. Due to problems in amplifying mature miR9653a, RT-qPCRs were performed on its precursor (pre-miR9653a). This miRNA was evaluated also in the additional NIL pair 2-2712/2-2674. No amplification was obtained in samples from R 2DL+ 2-2618 (mock and infected spikelets and rachis), possibly because of the low expression of pre-miR9653a in this genotype (Table 3). On the other hand, amplification was detected for all the S null 2-2890-related samples (Table 3) with a down-regulation in infected samples (both spikelets and rachis) vs. mock condition (Table 4). Unexpectedly, no amplification was detected in all samples of the NIL pair 2-2712 (2DL+) and 2-2674 (null) (Table 3). Differences in expression patterns between different pairs of NILs segregating for the same QTL have been recently highlighted by Long et al. (2015) who suggested the presence of additional elements contributing to resistance, other than the identified 2DL QTL.

**Table 4:** Comparison between NGS and RT-qPCR results for the pair of NILs R 2DL+ 2-2618/S null 2-2890.

	**2-2890 (null) — Fg vs. H**_**2**_**O**				**2-2618 (2DL**+**) — Fg vs. H**_**2**_**O**				**H**_**2**_**O — 2-2618 (2DL**+**) vs. 2-2890 (null)**				**Fg — 2-2618 (2DL**+**) vs. 2-2890 (null)**			
	**Spikelet**		**Rachis**		**Spikelet**		**Rachis**		**Spikelet**		**Rachis**		**Spikelet**		**Rachis**	
	**NGS**	**qPCR**	**NGS**	**qPCR**	**NGS**	**qPCR**	**NGS**	**qPCR**	**NGS**	**qPCR**	**NGS**	**qPCR**	**NGS**	**qPCR**	**NGS**	**qPCR**
miR9774	2.14	3.86	nd	1.88	−2.89	−2.27	nd	−1.72	3.72	3.57	nd	2.33	nd	−1.83	nd	1.89
pre-miR9653a	−4.23	−2.63	−2.42	−2.69	nd	nd	nd	nd	−1,019.5	nd	−255.27	nd	−482	nd	−116	nd
miR9666a	−7.86	−2.32	−8.42	−2.86	nd	1.06	nd	1.35	−17.30	−4.96	−71	−5.43	−3.67	−2.45	−29.5	−3.18
miR164	−11.86	−3.13	−12.43	−4.17	−2.79	−2.22	nd	−1.02	nd	−1.22	nd	−1.65	nd	−1.18	5.36	2.44
miR167c	−14.85	−3.7	−8.12	−2.56	−3.58	−2.28	nd	−1.49	−2.45	−1.79	nd	−1.27	2.45	2.63	nd	1.33
miR168	−9.05	−5	−4.54	−2.27	−3.52	−2.04	nd	−1.37	nd	−1.06	nd	−1.16	3.38	2.56	nd	−1.4
miR9653b	nd	1.22	nd	1.43	nd	−1.47	nd	−1.04	2.54	2	nd	−1.25	nd	−1.09	nd	1.19
miR398	−77.29	−4.88	−35	−5.03	nd	−1.87	nd	−1.15	nd	1.3	nd	1.65	nd	−1.43	nd	−1.24
fox-milRNA-2c-d-e	nd	nd	66.67	nd	nd	nd	192.94	nd	nd	nd	1	nd	−2.13	−6.9	−2.86	−2
fox-milRNA7	274.68	nd	35.44	nd	191.17	nd	329.74	nd	1.5	nd	−9.09	nd	1.05	1.11	1.01	1.28
Fg-milRNA-1	342.33	12,011.8	4.59	314.94	4.87	nd	2.35	83.48	45.67	nd	1.37	nd	−7.69	−3.33	−1.43	−1.85

To assess the transcription of the *WIR1* genes in response to FHB infection, RT-qPCRs for WIR1-like, the putative target of miR9653a, and for all the members belonging to the WIR1 family present in the NCBI database (WIR1-A—HQ337015, a putative target of fox_miRNA_7, WIR1-B—HQ337016, and WIR1-C—HQ337017; Table 3 and Figure 7) were conducted for the two pairs of NILs. NILs 2-2618 (2DL+) and 2-2890 (null) showed induction of WIR1-like in FHB inoculated samples, and a general higher level of transcription for all the members of the gene family was observed in R 2DL+ 2-2618 compared to S null 2-2890 in both tissues and conditions, particularly during infection (Figure 7), supporting the putative role for *WIR1*-genes in the response to FHB. Moreover, the WIR1-like expression profile negatively correlated with miR9653a expression, highlighting a functional relationship between WIR1-like and miR9653a. The same general response was observed also when the expression of the same gene was assayed in the additional NIL pair 2-2712 (2DL+)/2-2674 (null), with the exception of WIR1-like, which was up-regulated during infection in both tissues of both lines, with higher modulation and expression in the null genotype, with respect to the 2DL+ one (Figure 7). All the results underline that miR9653a and its putative target WIR1-like could play a role in FHB response. The expression trend identified in the NGS experiment was confirmed by RT-qPCRs also for miR398 in the pair 2-2618/2-2890 (Table 4), but no significant transcriptional variation was found performing the analysis for the other pair of NILs (Figure 7).

For a further general validation of the NGS data, a sample of randomly selected miRNAs (miR9666a—MT22, miR9774—MT33, miR164—MT6, miR167c—MT17, miR168—MT8, and miR9653b—MT61) was tested by RT-qPCRs in 2DL+ 2-2618/null 2-2890 giving comparable results in term of up- and down-regulation with however different FCs (Table 4), thus confirming miRNA-Seq results.

## Discussion

### General transcriptional responses to FHB infection in the resistant and susceptible NILs

**Figure 8:**
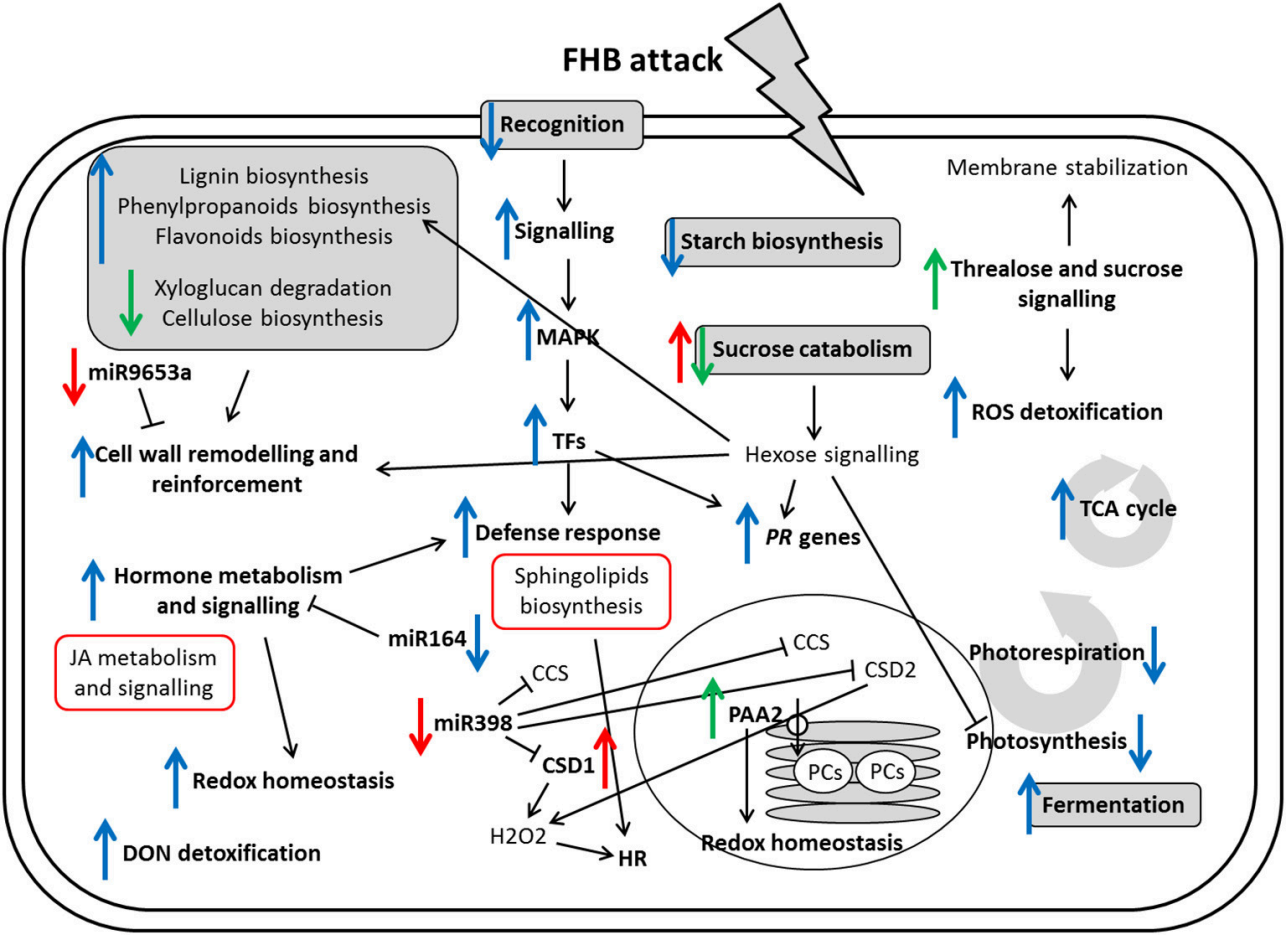
Summary of the cascades of biochemical events during the crosstalk between the two NILs and FHB. Blue arrows indicate a modulation implicated in basal defense response. Red arrows indicate modulation after infection in the susceptible null 2-2890 NIL only. Green arrows indicate modulation after infection in the resistant 2DL+ 2-2618 NIL only and thus a likely involvement in the 2DL specific resistance. Red boxes indicate higher expression in S null 2-2890 with respect to R 2DL+ 2-2618. CCS, copper chaperone for superoxide dismutases; CSD, Cu/Zn superoxide dismutase; HR, hypersensitive response; MAPK, mitogen-activated protein kinase; PAA2, P-Type ATPase 2; PCs, plastocyanins; TCA, tricarboxylic acid; TFs, transcription factors.

Figure 8 represents a summary of all the pathways induced or repressed by infection in the two genotypes.

Down-regulation of the primary and energy metabolism in response to FHB was oserved, as many genes implicated in these processes were modulated by infection in both NILs. Down-regulation of photosynthesis and primary metabolism in general has been reported in different plant-pathogen interactions (Denoux et al., 2008; Bilgin et al., 2010; Windram et al., 2012), including wheat-*F. graminearum*, for which an elevated demand of carbohydrates and energy equivalent during FHB response is request (Kugler et al., 2013). Thus, the breakdown of photosynthesis and primary metabolism might replenish energy equivalent from carbohydrates to alleviate the energy costs associated with the up-regulation of other metabolic pathways, leading to the shift from source to sink which may enhance the activation of defense responses (Bolton, 2009).

A general up-regulation of enzymes implicated in lignin and phenylpropanoid biosynthesis with essential roles in plant disease resistance (Miedes et al., 2015) as well as of chalcones, flavonols, dihydroflavonols, and anthocyanins biosynthetic pathways was detected for both genotypes and tissues after infection. Therefore, in our experimental system cell wall remodeling and reinforcement might play central roles in basal response to FHB, opposing the action of the cell wall degrading enzymes produced by the fungus during the infection process (e.g., pectinases, cellulases, and xylanases; Bellincampi et al., 2014). These pathways were previously found associated to resistant responses to FHB. Indeed, Lionetti et al. (2015) analyzed cell wall composition of FHB infected spikes at anthesis of a R bread wheat and a S durum wheat and observed that the R cell wall was enriched of S-type lignin, and methylesterified pectin, which reduces the effect of fungal polygalacturonases (Volpi et al., 2011; Bethke et al., 2014). In addition, hemicellulose of the R genotype was enriched in arabinose, galactose, and glucose, instead of xylose (Lionetti et al., 2015). Moreover, cell wall reinforcement was also associated with the wheat *Fhb1* QTL-specific resistance, which is correlated with an accumulation of hydroxycinnamic acid amides, phenolic glucosides, and flavonoids in cell walls of infected spikelets and rachis (Gunnaiah et al., 2012).

Altough accumulation of salicylic acid (SA), jasmonic acid (JA), abscissic acid (ABA), and indole acetic acid during *F. graminearum* infection in wheat was recently demonstrated by Qi et al. (2016), with few exceptions represented by JA (below discussed), the activation of hormons biosynthesis and signal transduction pathways was recorded as general response to FHB infection in all the samples analyzed. Similarly, genes related to UDP-glycosyltrasferases, which participate in DON detoxification (Kugler et al., 2013; Schweiger et al., 2013; Li et al., 2015), were induced in both R and S NILs.

Several genes involved in redox homeostasis and transcriptional regulation were identified as commonly modulated in both R and S NILs. However, several DEGs showing differential modulation were also identified (discussed below), indicating a putative role in FHB resistance governed by the 2DL resistance QTL.

### Transcriptional responses associated to the 2DL QTL for FHB resistance

Several genes involved in defense processes were identified as differentially regulated among the two NILs, both in terms of expression in the R NIL only, regardless of the FHB infection, and/or specifically modulated (induced or repressed) in the R NIL.

#### Cell wall metabolism

Two loci were more transcribed in the R NIL, Traes_3al_194ff20fa gene, encoding for cinnamyl-alcohol dehydrogenase, an enzyme involved in the phenylpropanoid metabolism and lignin biosynthesis and implicated in disease resistance in wheat (Mitchell et al., 1999) and Traes_2dl_0dffd3ce2, corresponding to a putative alpha-L-fucosidase implicated in xyloglucan degradation, removing xylose from xyloglucan 1 precursor thus making cell wall more resistant to fungal hydrolytic enzymes (Bellincampi et al., 2014). A further contribution in increasing of cell wall reinforcement in the R 2DL+ 2-2618 NIL might be attributed to the Traes_1bl_f026049bc locus, corresponding to an O-methyl transferase; this gene, induced by infection in the R genotype only and more expressed in R vs. S in infected spikelets, was implicated in wheat resistance to stripe rust (Liu et al., 2006) and in cell wall reinforcement through an involvement in suberin biosynthesis (Held et al., 1993). Moreover, several DEGs encoding cellulose synthases and a beta-1,4-glucanase were repressed by infection in the R genotype. Since *F. graminearum* utilizes cellulases and hemicellulases to degrade the principal components of primary plant cell wall (Carapito et al., 2013), the inhibition of these genes in the R genotype after the pathogen infection might contribute to the remodeling of cell wall during pathogen attack, while the higher expression observed in S infected spikelets might contribute to the higher invasion of the fungus in S tissues.

The miRNA-Seq experiment identified miR9653a as more expressed in S than in R NILs. A putative target was identified in a member of the WIR1 family, a gene family well-known for being involved in cell wall reinforcement, increasing adhesion of cellular membrane to cell wall during pathogen attack (Coram et al., 2010). This result is in agreement with the work of Long et al. (2015) where a member of the WIR1 family, WIR1A, was more expressed in the R 2DL+ 2-2618 spikelets than in the S null 2-2890 spikelets during *F. graminearum* infection. Although WIR1A has not been functionally characterized, it is thought to play a role in basal and non-host resistance and two WIR1A variants were mapped within the interval of a wheat genomic locus on chromosome 7BS that confers FHB resistance (Diethelm et al., 2012). Thus, the high level of miR9653a identified in the S null 2-2890 and the higher expression of WIR1A (and other members of the WIR1 family) in the R NILS might contribute to the differential response to FHB recorded among the two NILs.

#### Sugar signaling

Several DEGs showing higher expression in R vs. S NILs regardless of FHB infection encoded for gene functions involved in responses to trehalose stimulus. These included Traes_3as_c082cdcfe (encoding a putative plastidic glucose transporter), Traes_3as_acac429eb (encoding a SNL TF), Traes_3as_6626d4a69 (encoding a MYB domain-containing TF), and Traes_3as_a3ce60e4f (encoding for a WD40 repeat protein). Trehalose represents a ROS (reactive oxygen species) quencher in wheat during infection and confers partial protection to wheat challenged by powdery mildew (Renard-Merlier et al., 2007). Moreover, trehalose-6-P is a regulator of the sucrose pathway and both sucrose and trehalose trigger membrane stabilization. During biotic and abiotic stresses, sucrose activates the biosynthesis of fructans in wheat and anthocyanins in *A. thaliana* through the activation of MYB TFs that interact with WD40 repeat proteins, thus enhancing ROS detoxification (Teng et al., 2005; Xue et al., 2011; Van den Ende and El-Esawe, 2014). Furthermore, Traes_3dl_8c6d663c5 (encoding a Protein Phosphatase 2C (PP2C), homolog to the *A. thaliana* ABI1), was repressed by infection in R tissues and less expressed in R infected spikelets compared to the S NIL. ABA and energy signaling pathways interact through PP2Cs inhibition and the *Arabidopsis* ABI1 represents a SnRK1 phosphatase that inactivates the energy sensor SnRK1 (Rodrigues et al., 2013). It was demonstrated that stress-derived energy deficiency activates the Sucrose non-fermenting-1-related proteins (SnRKs), a class of energy sensors and regulators induced by starvation and stresses and promoting up-regulation of catabolic processes, together with inhibition of primary metabolism, to maintain cellular energy homeostasis (Van den Ende and El-Esawe, 2014). The lower transcription of Traes_3dl_8c6d663c5 in R spikelets, with respect to S ones, migth contribute in maintaining SnRK activity, supporting the implication of sugar signaling in the 2DL QTL FHB resistance, while the unresponsiveness and the higher expression detected in the S infected spikelets may contribute to the S null 2-2890 susceptibility.

Repression and induction by infection were respectively observed in R and S spikelets for Traes_6as_ac24f31a6 which encodes for gene functions related to sucrose catabolism. The accumulation of hexoses promotes the induction of PR genes, the down-regulation of photosynthesis, the triggering of phenylpropanoids and anthocyanins biosynthesis and the deposition of callose in cell wall (Tauzin and Giardina, 2015). Although a role for the activation of Traes_6as_ac24f31a6 in S null 2-2890 needs to be investigated, the MT377 detected for this gene might underline two different strategies likely displayed by the two NILs: activation of sucrose signaling, with inhibition of its catabolism, leading to membrane stabilization for R 2DL+ 2-2618, and activation of defense response through hexose signaling for S null 2-2890, that however is not able to stop fungal growth.

#### JA metabolism and signaling

Several loci involved in JA metabolism and signaling were more expressed in the S NIL, in comparison to the R one. These included: (i) Traes_6as_70b2f2ae4, encoding for the cytochrome P450 CYP74, that functions as an allene oxide synthase, catalyzing the dehydration of the hydroperoxide to an unstable allene oxide in the JA biosynthetic pathway; (ii) Traes_1ds_75af80583 and Traes_1bs_527ed00b9, encoding two C2H2 zinc finger TFs which activate DREB expression and promote the resistance against plant pathogens mediated by JA and ABA (Deb et al., 2016). Makandar et al. (2010) found *Arabidopsis* mutants in the JA pathway hyperresistant to *F. graminearum* and demonstrated that the JA pathway contributes to *F. graminearum* susceptibility by attenuating the activation of SA signaling. Thus, the higher expression of the JA related genes in the S null 2-2890, with respect to its R NIL, might contribute to its susceptibility.

#### Receptors and signal transduction

Several genes involved in signal transduction were induced by FHB infection in the R NIL only or showed higher transcription level in the R NIL in comparison to the S one. These included Traes_2dl_7788247ee, encoding a LRR transmembrane protein kinase belonging to membrane receptors implicated in pathogens recognition (Monaghan and Zipfel, 2012), Traes_3as_bc76a2e7e, a LRR kinase, Traes_3ds_072b70fd7, homolog to wheat LRK10-like kinase, and Traes_3ds_5870f8f48, homolog to thaumatin-like kinase. Furthermore, these last two proteins were also classified as SNC4 (suppressor of npr1-1, constitutive 4), an atypical receptor-like kinase with two predicted extracellular glycerophosphoryl diester phosphodiesterase domains, involved in lipid degradation and conferring resistance to *Hyaloperonospora arabidopsidis* in *Arabidopsis* (Bi et al., 2010). Finally, also the RLK receptor kinase encoding gene Traes_1as_581d331e0 was induced by infection in R spikelets only.

Lower mRNA levels were recorded in the R NIL, with respect to the S one, for Traes_2dl_7b0056729 (MT103), a gene encoding a serinc-domain containing protein, involved in the biosynthesis of sphingolipids, a class of plant lipids with a role in plant-pathogen interactions and in programmed cell death (PCD) as being second messengers in disease response (Sperling and Heinz, 2003). This observation is in agreement with the hypothesis that hemibiotrophic and necrotrophic pathogens promote PCD leading to the production of PCD-inducing factors, such as sphingolipids, in order to facilitate their penetration and spreading inside plant cells (Magnin-Robert et al., 2015). Thus, the higher expression observed in S null 2-2890 might participate to enhance the disease susceptibility observed for this genotype increasing cell death activation and FHB penetration in its tissues.

The miRNA experiment identified miR9863a and miR9863b as up-regulated in the R vs. S comparisons. The miR9863 family plays a key role in dampening resistance to powdery mildew in barley, targeting a group of MLA immune receptors leading to HR (Liu et al., 2014), suggesting that these two members might participate even to control the FHB response in the R NIL.

#### Regulation of the redox status

The Traes_2dl_03caa3b80 gene, encoding a P-Type ATPase 2 (PAA2) was induced by infection in R spikelets and more expressed in R vs. S NILs. PAA2 is a thylakoid located Cu transporter required for the accumulation of Cu in plastocyanins. Since it is implicated in the maintenance of Cu homeostasis in chloroplasts (Tapken et al., 2012), the higher expression detected in R 2DL+ 2-2618, with respect to the S null 2-2890, might result in a higher activity and in a more efficient maintenance of chloroplast Cu and redox homeostasis during FHB infection in the R 2DL+ 2-2618 NIL with respect to S null 2-2890.

Moreover, we observed that miR398 was down-regulated by infection in S null 2-2890 tissues. miR398 targets two Cu/Zn superoxide dismutases (CSDs), the cytosolic CSD1 and CSD2, localized in the stroma, and the copper chaperone for superoxide dismutase (CCS) which lets CSDs to receive their Cu cofactor (Abdel-Ghany and Pilon, 2008). In barley-powdery mildew interaction, an accumulation of chloroplast Cu/Zn SOD1 (HvSOD1) was a consequence of the activity of the *Mla* and *Rar1* genes which negatively regulate also miR398 that, in turn, targets HvSOD1. Moreover, the silencing of HvSOD1 impeded *Mla*-triggered H_2_O_2_ and hypersensitive response (HR) at barley-*B. graminis* interaction sites, suggesting that HvSOD1-generated H_2_O_2_ contributes to ROS accumulation and enhances *Bgh* response in barley (Xu et al., 2014). Furthermore, induction after infection in the S NIL was recorded for two genes (Traes_2ds_3c3a2a12a and Traes_2bs_6015bc7c6) homolog to *Arabidopsis* CSD1. For several necrotroph pathogens, like *F. graminearum*, it was demonstrated that HR promotes virulence (Coll et al., 2011). In the R NIL, higher levels of miR398 (leading to degradation of transcripts encoding for Cu/Zn superoxide dismutases) together with a more efficient maintenance of redox homeostasis through induction of the PAA2 gene might reduce HR. Conversely, in the S NIL, induction of CSD1 and reduced degradation of transcripts for Cu/Zn superoxide dismutases (promoted by the down-regulation of mi398) might lead to opposite responses, possibly driving higher level of susceptibility to the infection of the FHB necrotrophic pathogen.

#### Transcriptional regulation

Traes_3as_6626d4a69, encoding a MYB domain-containing protein and Traes_4bs_2ea81743e1, encoding a Chitin-inducible gibberellin-responsive GRAS TF, were both more expressed in R than S infected spikelets. GRAS TFs are involved in nodulation during symbiosis interaction (Gobbato et al., 2012) and were found to respond to chitin, an elicitor of plant defenses, in *A. thaliana* (Libault et al., 2007). The higher expression of such genes in the R 2DL+ 2-2618 NIL, with respect to the S null 2-2890, might suggest an implication in the 2DL FHB resistance.

Among the miRNAs differentially regulated, we observed that miR164 was more repressed by infection the S NIL and more expressed in the infected rachis of the R NIL, with respect to the S one. miRNA164 is implicated in the response to abiotic stresses, like drought and salinity (Golldack et al., 2011) and targets NAC TFs, which are implicated in abiotic stress response and are regulators of plant disease resistance (Nuruzzaman et al., 2013). For example, the barley *HvNAC6* gene enhances basal resistance to powdery mildew by affecting ABA accumulation (Chen et al., 2013). According to literature, predicted putative targets of the miR164 family were identified among DEGs differentially regulated among R and S NILs (Traes_7dl_91cfe828f and Traes_7al_628a69311). Lower levels of Traes_7dl_91cfe828f and Traes_7al_628a69311 transcripts were respectively detected in infected spikelets (MT25) and both tissues (MT22) of R 2DL+ 2-2618 in comparison to the S null 2-2890 (Table 1; Supplementary Data Sheet 1-Sheet 1; Supplementary Data Sheet 2-Sheet 2). This behavior might be related to the negative effect of miR164. This miRNA was also identified as implicated in the regulation of auxin signaling, since it was demonstrated that TFs belonging to the TCP class activate miR164 expression and inhibit auxin signaling and transport (Koyama et al., 2010). Down-regulation after infection of TCP TFs was observed, for S null 2-2890, in the RNA-Seq analysis, as 4 MT3, 2 MT8, and 4 MT19 TCP loci were repressed by infection in S spikelets (Table 1 and Supplementary Data Sheet 1-Sheets 1, 2). Thus, the down-regulation of these TFs might result in the repression of miR164, confirming a fine regulation of cellular processes implicated in FHB response, and might correlates with the differential expression detected in auxin metabolism-related genes observed in the RNA-Seq experiment.

## Conclusions

A comprehensive transcriptomic analysis (mRNA and miRNA) of spikelets and rachis from two bread wheat NILs, differing for the presence of the 2DL FHB resistance QTL, inoculated with *F. graminearum* at 3 dpi and mock, was performed to gain new insight in the molecular response associated with FHB tolerance. A general common defense response to infection was observed in both genotypes even though the S null 2-2890 mounted an amplified reaction, in terms of number of modulated genes and intensity of modulation, with respect to its R 2DL+ NIL 2-2618. The FHB basal disease response implicates an alteration of cell metabolism, that goes through the inhibition of photosynthesis and primary metabolism in favor of the activation of fermentation and secondary metabolism, the activation of cell wall reinforcement and remodeling, the induction of hormone metabolism and signaling and the triggering of ROS scavenging (Figure 8). The basal disease response processes involving miRNAome involved the targeting of hormone signaling, including auxin signal cascade, superoxide dismutase activities and genes implicated in pathogen resistance responses (Figure 8). Despite the fact that the 2DL FHB resistance QTL has not been precisely located yet, genes associated with the 2DL QTL-specific resistance, on the base of their induction after infection in the R 2DL+ 2-2618 or their differential expression between the two NILs were identified: e.g., LRR transmembrane kinase, P-type ATPase 2, required for the functionality of PCs for photosynthesis electron transport and Cu and redox homeostasis in chloroplasts, and alpha-fucosidase (Figure 8). Moreover different defense response strategies associated with sugar signaling were likely displayed by the two NILs: activation of membrane stabilization through sucrose signaling for R 2DL+ 2-2618, thus suggesting sucrose implication in the 2DL QTL resistance, and activation of basal disease response through hexose signaling for S null 2-2890 (Figure 8). Two genes associated with the null 2-2890 susceptibility were discovered: a serinc-domain containing protein encoding gene, involved in the synthesis of phosphatidylserine and sphingolipids, and the miR9653a corresponding gene. miR9653a was much more expressed in S null 2-2890, with respect to R 2DL+ 2-2618, and putatively targets a gene belonging to a gene family implicated in plant disease response. This work provides new perspectives to better understand FHB resistance/susceptibility in wheat. Future researches will be addressed to deeply validate and characterize the putative candidate resistance genes and the putative targets of the miRNAs here identified and their functions, thus clarifying the regulatory networks implicated in the defense response related to the 2DL FHB resistance QTL in wheat.

## Author contributions

CB performed library preparation, RT-qPCRs, RNA-Seq, and smallRNA analyses and statistical analyses. PB performed bioinformatic analysis of the RNA-Seq experiment. PF contributed to the bioinformatic analysis of the smallRNA-Seq experiment. XH conducted RT-qPCRs for mRNAs. MB performed plant infection, sampling, phenotyping and RNA extractions. MM contributed to RNA-Seq data analysis. ZY contributed to the writing. TO, LC, and GV conceived the experiments. The manuscript was drafted by CB and reviewed by all other authors.

### Conflict of interest statement

The authors declare that the research was conducted in the absence of any commercial or financial relationships that could be construed as a potential conflict of interest.

## Abbreviations

2DL+: presence of the 2DL FHB resistance QTL
ABA: abscissic acid
bHLH: basic helix loop helix
CCS: copper chaperone for superoxide dismutases
CSD: Cu/Zn superoxide dismutase
Cv: cultivar
DEG: differentially express gene
DGE: differential gene expression
DON: deoxynivalenol
Dpi: days post-inoculation
ET: ethylene
FC: fold change
FDR: false discvovery rate
Fg: *Fusarium graminearum*
FHB: fusarium head blight
IDT: integrated DNA technologies
GO: gene onthology
HR: hypersensitive response
HvSOD1: barley superoxide dismutase 1
JA: jasmonic acid
LRR: leucine rich repeat
milRNA: miRNA-like RNA
miRNA: microRNA
mRNA: messenger RNA
MT: modulation type
ncRNA: non-coding RNA
NGS: next generation sequencing
NIL: near isogenic line
PAA2: P-Type ATPase 2
PCA: principal component analysis
PCD: programmed cell death
PP2C: protein phosphatase 2C
PR: pathogenesis related
PTF: pore-forming toxin-like
RT-qPCR: quantitative real time PCR
QTL: quantitative trait *locus*
R: resistant
RIL: recombinant inbred line
ROS: reactive oxygen species
RPKM: reads per kilobase per million
S: susceptible
SA: salicylic acid
SNC4: suppressor of npr1-1, constitutive 4
SNL: SIN3-Like
SnRKs: sucrose non-fermenting-1-related protein
sRNA: smallRNA
TF: transcription factor
WAK: cell wall-associated kinase.
