# Supplementary material for: Comparative Transcriptome Profiles of Near-Isogenic Hexaploid Wheat Lines Differing for Effective Alleles at the 2DL FHB Resistance QTL
Source: Front Plant Sci. 2018 Jan 30;9:37. doi: 10.3389/fpls.2018.00037 (PMC5797473; doi:10.3389/fpls.2018.00037)
Supplement: Supplementary file 1 [file Table1.DOCX]

**Supplementary Table 1.** List of primers utilized in the RT-qPCR analyses. The corresponding *loci*, primer names and sequences are indicated. SL = stem loop primer, FW = forward primer, REV = reverse primer.

| ***Locus*** | **Primer name** | **Primer Sequence** |
| --- | --- | --- |
| Traes_2dl_03caa3b80 | Traes_2dl_03caa3b80_FW | CCACATGCAACCAGACAGA |
|  | Traes_2dl_03caa3b80_REV | ACCGAACCTAAGACCGATAAC |
| Traes_2dl_0dffd3ce2 | Traes_2dl_0dffd3ce2_FW | CTGGTTTGCGCTTATCCATC |
|  | Traes_2dl_0dffd3ce2_REV | CACGACGGTCTTGTTGAAAG |
| Traes_2dl_a208876fe | Traes_2dl_a208876fe_FW | CCCACATAGCTGTGAAGTTTG |
|  | Traes_2dl_a208876fe_REV | GAGGAGGGTAGTTGGTGAAG |
| Traes_2dl_7788247ee | Traes_2dl_7788247ee_FW | TAACGGCTCACTTCCAGATG |
|  | Traes_2dl_7788247ee_REV | GTATCTGCCCGGTGAAACTA |
| Traes_2dl_179570792 | Traes_2dl_179570792_FW | GGAATGGCTTGTGGATCTTG |
|  | Traes_2dl_179570792_REV | GCCTTGCGGAATAATGTACG |
| Traes_2dl_89a313ac3 | Traes_2dl_89a313ac3_FW | GAACCTGCCGAATGTCTATC |
|  | Traes_2dl_89a313ac3_REV | GAGCATCAGTATTCCACCAC |
| Traes_2dl_3040097a4 | Traes_2dl_3040097a4_FW | ATGGTGCCATGGAGATTCA |
|  | Traes_2dl_3040097a4_REV | CCGGGACACTCTTTGTTCTA |
| Traes_3as_b45d1d4fc | Traes_3as_b45d1d4fc_FW | GACCTTGAGTTGGATTGTTCTC |
|  | Traes_3as_b45d1d4fc_REV | GCAACTACTCTCCCATCCTT |
| Traes_3ds_cfc93f8b4 | Traes_3ds_cfc93f8b4_FW | CCATTCGACTACGGCGGT |
|  | Traes_3ds_cfc93f8b4_REV | GGTGCACCCGAAGAATTTA |
| Traes_3as_272105d49 | Traes_3as_272105d49_FW | ATGGTTTCTCCTTTCCGAGAC |
|  | Traes_3as_272105d49_REV | TAGGGATCCATAGGACGCTATC |
| AOx | AOx_FW | GACTTGTCATGGTAGATGCCTG |
|  | AOx_REV | CAGGACGAGCATAACCATTCTC |
| w-GAPDH | w-GAPDH_FW | AACTGTTCATGCCATCACTGCCAC |
|  | w-GAPDH_REV | AGGACATACCAGTGAGCTTGCCAT |
| hn-RNP-Q | hn-RNP-Q_FW | TCACCTTCGCCAAGCTCAGAACTA |
|  | hn-RNP-Q_REV | AGTTGAACTTGCCCGAAACATGCC |
| Tae-miR9653b | miR9653b_SL | GTCGTATCCAGTGCAGGGTCCGTGGTATTCGCACTGGATACGACAGCCTC |
|  | miR9653b_FW | TTATTCCATGGCCAAGGTCTCTT |
| Tae-miR164 | miR164_SL | GTCGTATCCAGTGCAGGGTCCGAGGTATTCGCACTGGATACGACGTGCTC |
|  | miR164_FW | CGGCGGTTGACAGAAGAGAGT |
| Tae-miR167c | miR167_SL | GTCGTATCCAGTGCAGGTCCGAGGTATTCGCACTGGATACGACTCAGAT |
|  | miR167_FW | CAGGCTGAAGCTGCCAGCATG |
| Tae-miR168 | miR168_SL | GTCGTATCCAGAGCTGGGTCCGAGGTATTCGCTCTGGATACGACGTCCCG |
|  | miR168_FW | TCGTTCGCTTGGTGCAGAT |
|  | miR168_REV | GAGCTGGGTCCGAGGT |
| Tae-miR9666a | miR9666_SL | GTCGTATCCAGAGCTGGGTCCGAGGTATTCGCACTGGATACGACTGTTCAA |
|  | miR9666_FW | GGGCGTACTGTGGGCACTTAT |
| Tae-miR398 | miR398_SL | GTCGTATCCAGTGCAGGGTCCGTGGTATTCGCACTGGATACGACCGGGGG |
|  | miR398_FW | TCGCGTGTGTTCTCAGGTCG |
| Fg-milRNA-1 | milRNA1_SL | GTCGTATCCAGTGCAGGGTCCGAGGTATTCGCACTGGATACGACGAACCT |
|  | milRNA1_FW | TCGCGTCCGGTATGGTGTAG |
| fox-milRNA-2c-d-e | fox-milRNA2_SL | GTCGTATCCAGTGCAGGGAGGGAGGTATTCGCACTGGATACGACGAACC |
|  | fox-milRNA2_FW | TCTTATTGTGTTCGCACGCGTA |
|  | fox-milRNA2_REV | GTGCAGGGAGGGAGGT |
| fox-milRNA7 | fox-milRNA7_SL | GTCGTATCCAGTGCAGGGTCCGAGGTATTCGCACTGGATACGACGAACCT |
|  | fox-milRNA7_FW | GTGCGCCGTAGTATAGTGGTCAG |
| pre_Tae-miR9653a | pre_Tae-miR9653a_FW | CATCGCTTTGGTTCCCAGG |
|  | pre_Tae-miR9653a_REV | TCACGCGAACCTCACGAGA |
| Ta-snoR10 | Tae-SnoR10_FW | GGATGAAACCTTCAAACAATCTTA |
|  | Tae-SnoR10_REV | TCATTCACAGGAAAATAAGCACTG |
| WIR1-like - CA673319 | CA673319_FW | GCCGAGGTCTCAAAGCCT |
|  | CA673319_REV | TGCTGATGATTCGATGCTGC |
| WIR1A | WIR1A_FW | TCGAATCATCACCATGGCGT |
|  | WIR1A_REV | GACGACGAAGAAGAGAGCGA |
| WIR1B | WIR1B_FW | TCATCAACAGCTCCGTCTGT |
|  | WIR1B_REV | ACGTTAGGGTCGAGAGCAC |
| WIR1C | WIR1C_FW | ATCGCTCTCTTCTTCGTCGT |
|  | WIR1C_REV | CAGACGGAGCTGTTCATGATG |
| WubiG | WubiG_FW | TTTGCCGGTTGATTGAAGTG |
|  | WubiG_REV | TCCAAGTACAGGTGACAGCTGACT |
| REV uni | | GTGCAGGGTCCGAGGT |
| REV 3628 | | GTGCAGGGTCCGTGGT |
