# Supplementary material for: Comparative Transcriptome Profiles of Near-Isogenic Hexaploid Wheat Lines Differing for Effective Alleles at the 2DL FHB Resistance QTL
Source: Front Plant Sci. 2018 Jan 30;9:37. doi: 10.3389/fpls.2018.00037 (PMC5797473; doi:10.3389/fpls.2018.00037)
Supplement: Supplementary file 2 [file Table2.DOCX]

**Supplementary Table 2.** Summary of the RNA-Seq and miRNA-Seq experiments. The numbers of raw reads generated by RNA-Seq and miRNA-Seq experiments in each of the 24 samples are reported. The amounts of RNA-Seq reads mapped on the wheat genome considering multiple or single mapping positions, of miRNA-Seq reads obtained after adapter trimming and size selection (18-30nt) and the amount of reads mapped on the *F. graminearum* genome (Fg) are indicated. S = susceptible null genotype 2-2890; R = resistant 2DL+ genotype 2-2816; sp = spikelet; ra = rachis; Fg = *F. graminearum* infected sample; H_2_O = mock sample; 1 = first biological replicate; 2 = second biological replicate; 3 = third biological replicate.

| **Sample** | **RNA-Seq** | | | | **miRNA-Seq** | | |
| --- | --- | --- | --- | --- | --- | --- | --- |
|  | **Total reads** | **Total mapping position** | **Single mapping position** | **Fg** | **Total reads** | **Adapter trimmer 18-30nt** | **Fg** |
| S_sp_Fg_1 | 24931328 | 43929134 | 11749244 (47.13%) | 5100950 (20.46%) | 6534826 | 2070683 (31.69%) | 569808 (27.52%) |
| S_sp_Fg_2 | 15484153 | 26664929 | 9904663 (63.97%) | 1653708 (10.68%) | 3391702 | 989732 (29.18%) | 263925 (26.67%) |
| S_sp_Fg_3 | 16934673 | 27583178 | 9843007 (58.12%) | 2413191 (14.25%) | 5457706 | 1635000 (29.96%) | 403789 (24.7%) |
| S_sp_H2O_1 | 14512293 | \| 32412555 \| \| --- \| | 11862844 (81.74%) | 8707 (0.06%) | 5705633 | 3935923 (68.98%) | 123549 (3.14%) |
| S_sp_H2O_2 | 19886380 | 44118871 | 16129488 (81.11%) | 7954 (0.04%) | 4787236 | 3185579 (66.54%) | 110539 (3.47%) |
| S_sp_H2O_3 | 18939725 | 40959962 | 15619241 (82.47%) | 5681 (0.03%) | 5297540 | 3559101 (67.18%) | 118159 (3.32%) |
| S_ra_Fg_1 | 23689591 | \| 39207589 \| \| --- \| | 12592486 (53.16%) | 4214378 (17.79%) | 5396976 | 1892550 (35.07%) | 480607 (25.39%) |
| S_ra_Fg_2 | 19167218 | 33994920 | 13437328 (70.11%) | 1230535 (6.42%) | 3811120 | 1461953 (38.36%) | 221708 (15.17%) |
| S_ra_Fg_3 | 9230167 | 15845516 | 6043327 (65.47%) | 839022 (9.09%) | 3857147 | 1410744 (36.57%) | 262394 (18.6%) |
| S_ra_H2O_1 | 15290022 | \| 32729764 \| \| --- \| | 12451982 (81.44%) | 7645 (0.05%) | 3893550 | 2506145 (64.37%) | 99255 (3.96%) |
| S_ra_H2O_2 | 18454732 | 39665751 | 14981115 (81.18%) | 5536 (0.03%) | 4165987 | 2810280 (67.46%) | 118981 (4.23%) |
| S_ra_H2O_3 | 18632207 | 40163345 | 14787994 (79.37%) | 16769 (0.09%) | 4573475 | 2980592 (65.17%) | 131526 (4.41%) |
| R_sp_Fg_1 | 21289752 | \| 42370333 \| \| --- \| | 16161539 (75.91%) | 655724 (3.08%) | 4313954 | 1742184 (40.38%) | 168132 (9.65%) |
| R_sp_Fg_2 | 13462663 | 26697529 | 10344163 (76.84%) | 424074 (3.15%) | 4830157 | 2017949 (41.78%) | 183786 (9.11%) |
| R_sp_Fg_3 | 11079135 | 22054283 | 8412755 (75.93%) | 403281 (3.64%) | 4677975 | 1823262 (38.98%) | 280168 (15.37%) |
| R_sp_H2O_1 | 14657097 | \| 31884018 \| \| --- \| | 12030533 (82.08%) | 4397 (0.03%) | 4344380 | 3051960 (70.25%) | 37036 (1.21%) |
| R_sp_H2O_2 | 14204319 | 31676640 | 11546723 (81.29%) | 5682 (0.04%) | 5357188 | 3640831 (67.96%) | 40881 (1.12%) |
| R_sp_H2O_3 | 15112788 | 33958049 | 12300161 (81.39%) | 7556 (0.05%) | 4958404 | 3285268 (66.26%) | 45886 (1.4%) |
| R_ra_Fg_1 | 33943223 | \| 67764623 \| \| --- \| | 27170339 (80.05%) | 573640 (1.69%) | 4429876 | 2315098 (52.26%) | 166149 (7.18%) |
| R_ra_Fg_2 | 16171610 | 33726405 | 12713310 (78.61%) | 291089 (1.80%) | 6907022 | 3613424 (52.32%) | 304574 (8.43%) |
| R_ra_Fg_3 | 11079135 | 22735048 | 9167969 (82.75%) | 224906 (2.03%) | 5222425 | 2700117 (51.70%) | 203712 (7.54%) |
| R_ra_H2O_1 | 16881094 | \| 34837929 \| \| --- \| | 13437443 (79.60%) | 5064 (0.03%) | 5074718 | 3388716 (66.78%) | 62670 (1.85%) |
| R_ra_H2O_2 | 19306722 | 40626777 | 15476380 (80.16%) | 5792 (0.03%) | 4911785 | 3190379 (64.95%) | 67.993 (2.13%) |
| R_ra_H2O_3 | 26953141 | 60097478 | 22334000 (82.86%) | 10781 (0.04%) | 5576545 | 3800480 (68.15%) | 92400 (2.43%) |
