# Supplementary material for: Comparative Transcriptome Profiles of Near-Isogenic Hexaploid Wheat Lines Differing for Effective Alleles at the 2DL FHB Resistance QTL
Source: Front Plant Sci. 2018 Jan 30;9:37. doi: 10.3389/fpls.2018.00037 (PMC5797473; doi:10.3389/fpls.2018.00037)
Supplement: Supplementary file 3 [file Table3.DOCX]

**Supplementary Table 3.** Reports of variance analyses conducted on the percentages of RNA-Seq

reads mapped on the *F. graminearum* genome for each sample (**A**) the percentages of miRNA-Seq

reads ranging from 18 to 30nt in each sample (**B**) and the percentages of miRNA-Seq reads mapped

on the *F. graminearum* genome for each sample (**C**). S = susceptible null genotype 2-2890; R =

resistant 2DL+ genotype 2-2816; sp = spikelet; ra = rachis; Fg = *F. graminearum* infected sample;

H_2_O = mock control sample.

**A**

| **Variables** | **Mean Square** | **F-ratio** | **p-value** |
| --- | --- | --- | --- |
| Genotype R/S | 167.376 | 22.329 | 0.000 |
| Tissue ra/sp | 11.234 | 1.499 | 0.239 |
| Treatment Fg/H2O | 364.728 | 48.657 | 0.000 |
| Genotype*Tissue | 2.458 | 0.328 | 0.575 |
| Genotype*Treatment | 166.532 | 22.217 | 0.000 |
| Tissue*Treatment | 11.289 | 1.506 | 0.237 |
| Genotype*Tissue*Treatment | 2.535 | 0.338 | 0.569 |
| Error | 7.496 |  |  |
| R^2^ | 0.926 |  |  |

**B**

| **Variables** | **Mean Square** | **F-ratio** | **p-value** |
| --- | --- | --- | --- |
| Genotype R/S | 274.997 | 127.91 | 0.000 |
| Tissue ra/sp | 80.74 | 37.555 | 0.000 |
| Treatment Fg/H2O | 4,422.735 | 2,057.154 | 0.000 |
| Genotype*Tissue | 12.155 | 5.654 | 0.030 |
| Genotype*Treatment | 215.64 | 100.301 | 0.000 |
| Tissue*Treatment | 173.882 | 80.878 | 0.000 |
| Genotype*Tissue*Treatment | 9.201 | 4.28 | 0.055 |
| Error | 2.15 |  |  |
| R^2^ | 0.993 |  |  |

**C**

| **Variables** | **Mean Square** | **F-ratio** | **p-value** |
| --- | --- | --- | --- |
| Genotype R/S | 361.616 | 69.249 | 0.000 |
| Tissue ra/sp | 26.797 | 5.132 | 0.038 |
| Treatment Fg/H2O | 1,102.428 | 211.114 | 0.000 |
| Genotype*Tissue | 3.197 | 0.612 | 0.445 |
| Genotype*Treatment | 194.826 | 37.309 | 0.000 |
| Tissue*Treatment | 54.18 | 10.375 | 0.005 |
| Genotype*Tissue*Treatment | 3.183 | 0.61 | 0.446 |
| Error | 5.222 |  |  |
| R^2^ | 0.954 |  |  |
