# Supplementary material for: Comparative Transcriptome Profiles of Near-Isogenic Hexaploid Wheat Lines Differing for Effective Alleles at the 2DL FHB Resistance QTL
Source: Front Plant Sci. 2018 Jan 30;9:37. doi: 10.3389/fpls.2018.00037 (PMC5797473; doi:10.3389/fpls.2018.00037)
Supplement: Supplementary file 5 [file Table5.DOCX]

**Supplementary Table 5.** Report of the reads annotated as milRNAs. For each sample the numbers of reads mapping at the level of each milRNA are reported. Yellow color highlights single reads which are present at least one time in all the biological replicates of a sample, while green colour refers to total number of reads for each milRNA and indicates those samples for which all the biological replicates have at least 1 read. Reads total numbers for each class and for each sample are also indicated. S = susceptible null genotype 2-2890; R = resistant 2DL+ genotype 2-2816; sp =spikelet; ra = rachis; Fg = *F. graminearum* infected sample; H_2_O = mock control sample.

|  |  |  | S_sp_Fg | | | S_sp_H2O | | | S_ra_Fg | | | S_ra_H2O | | | R_sp_Fg | | | R_sp_H2O | | | R_ra_Fg | | | R_ra_H2O | | |
| --- | --- | --- | --- | --- | --- | --- | --- | --- | --- | --- | --- | --- | --- | --- | --- | --- | --- | --- | --- | --- | --- | --- | --- | --- | --- | --- |
| Sequence | Length | Name | S_sp_Fg _1 | S_sp_Fg_2 | S_sp_Fg_3 | S_sp_H2O_1 | S_sp_H2O_2 | S_sp_H2O_3 | S_ra_Fg_1 | S_ra_Fg_2 | S_ra_Fg_3 | S_ra_H2O_1 | S_ra_H2O_2 | S_ra_H2O_3 | R_sp_Fg_1 | R_sp_Fg_2 | R_sp_Fg_3 | R_sp_H2O_1 | R_sp_H2O_2 | R_sp_H2O_3 | R_ra_Fg _1 | R_ra_Fg _2 | R_ra_Fg _3 | R_ra_H2O_1 | R_ra_H2O_2 | R_ra_H2O_3 |
| CCGGTATGGTGTAGTGGC | 18 | Fg-milRNA-1 | 14 | 9 | 20 | 0 | 0 | 0 | 43 | 28 | 17 | 1 | 2 | 2 | 7 | 5 | 8 | 0 | 1 | 0 | 21 | 20 | 14 | 0 | 0 | 5 |
| CCGGTATGGTGTAGTGGCT | 19 | Fg-milRNA-1 | 10 | 3 | 13 | 0 | 0 | 0 | 9 | 1 | 2 | 1 | 0 | 0 | 3 | 2 | 7 | 0 | 0 | 0 | 2 | 8 | 1 | 0 | 0 | 0 |
| CCGGTATGGTGTAGTGGCTA | 20 | Fg-milRNA-1 | 26 | 9 | 10 | 0 | 0 | 0 | 10 | 3 | 6 | 0 | 0 | 0 | 5 | 3 | 15 | 0 | 0 | 0 | 2 | 3 | 1 | 0 | 0 | 0 |
| CGGTATGGTGTAGTGGCT | 18 | Fg-milRNA-1 | 2 | 3 | 2 | 0 | 0 | 0 | 1 | 1 | 2 | 0 | 0 | 0 | 0 | 1 | 3 | 0 | 0 | 0 | 0 | 2 | 2 | 0 | 0 | 0 |
| CGGTATGGTGTAGTGGCTA | 19 | Fg-milRNA-1 | 5 | 4 | 1 | 0 | 0 | 0 | 3 | 0 | 0 | 0 | 0 | 0 | 1 | 0 | 1 | 0 | 0 | 0 | 0 | 4 | 2 | 0 | 0 | 0 |
| GGTATGGTGTAGTGGCTA | 18 | Fg-milRNA-1 | 3 | 1 | 1 | 0 | 0 | 0 | 1 | 0 | 0 | 0 | 0 | 0 | 0 | 0 | 2 | 0 | 0 | 0 | 0 | 2 | 1 | 0 | 0 | 0 |
| TCCGGTATGGTGTAGTGG | 18 | Fg-milRNA-1 | 6 | 5 | 11 | 0 | 0 | 0 | 16 | 7 | 14 | 1 | 0 | 0 | 3 | 1 | 3 | 1 | 2 | 1 | 8 | 7 | 4 | 0 | 2 | 2 |
| TCCGGTATGGTGTAGTGGC | 19 | Fg-milRNA-1 | 248 | 170 | 171 | 2 | 0 | 1 | 613 | 310 | 297 | 192 | 66 | 68 | 131 | 149 | 205 | 38 | 58 | 35 | 302 | 372 | 197 | 133 | 84 | 239 |
| TCCGGTATGGTGTAGTGGCT | 20 | Fg-milRNA-1 | 62 | 21 | 33 | 0 | 0 | 0 | 35 | 12 | 22 | 2 | 0 | 0 | 10 | 9 | 18 | 0 | 0 | 1 | 14 | 28 | 17 | 1 | 0 | 0 |
| TCCGGTATGGTGTAGTGGCTA | 21 | Fg-milRNA-1 | 87 | 40 | 37 | 0 | 0 | 0 | 56 | 23 | 28 | 4 | 1 | 0 | 21 | 19 | 35 | 0 | 0 | 0 | 8 | 33 | 20 | 0 | 0 | 0 |
|  |  | Total | 463 | 265 | 299 | 2 | 0 | 1 | 787 | 385 | 388 | 201 | 69 | 70 | 181 | 189 | 297 | 39 | 61 | 37 | 357 | 479 | 259 | 134 | 86 | 246 |
| AGCATTGTTGACAGGGCCTT | 20 | Fg-milRNA-12 | 0 | 0 | 0 | 0 | 0 | 0 | 0 | 0 | 1 | 0 | 0 | 0 | 0 | 0 | 0 | 0 | 0 | 0 | 0 | 0 | 0 | 0 | 0 | 0 |
| CAGCATTGTTGACAGGGC | 18 | Fg-milRNA-12 | 0 | 1 | 0 | 0 | 0 | 0 | 0 | 0 | 0 | 0 | 0 | 0 | 0 | 0 | 0 | 0 | 0 | 0 | 0 | 0 | 0 | 0 | 0 | 0 |
| CAGCATTGTTGACAGGGCC | 19 | Fg-milRNA-12 | 0 | 0 | 0 | 0 | 0 | 0 | 0 | 0 | 0 | 0 | 0 | 0 | 0 | 0 | 1 | 0 | 0 | 0 | 0 | 0 | 0 | 0 | 0 | 0 |
| CAGCATTGTTGACAGGGCCT | 20 | Fg-milRNA-12 | 0 | 0 | 0 | 0 | 0 | 0 | 0 | 0 | 1 | 0 | 0 | 0 | 0 | 0 | 0 | 0 | 0 | 0 | 0 | 0 | 0 | 0 | 0 | 0 |
| CAGCATTGTTGACAGGGCCTT | 21 | Fg-milRNA-12 | 0 | 0 | 0 | 0 | 0 | 0 | 0 | 0 | 0 | 0 | 0 | 0 | 0 | 1 | 0 | 0 | 0 | 0 | 1 | 0 | 0 | 0 | 0 | 0 |
| GCAGCATTGTTGACAGGGCC | 20 | Fg-milRNA-12 | 0 | 0 | 1 | 0 | 0 | 0 | 0 | 0 | 0 | 0 | 0 | 0 | 0 | 0 | 0 | 0 | 0 | 0 | 0 | 0 | 0 | 0 | 0 | 0 |
| GGCAGCATTGTTGACAGGGC | 20 | Fg-milRNA-12 | 1 | 0 | 1 | 0 | 0 | 0 | 0 | 0 | 0 | 0 | 0 | 0 | 0 | 0 | 0 | 0 | 0 | 0 | 0 | 0 | 0 | 0 | 0 | 0 |
| GGCAGCATTGTTGACAGGGCCT | 22 | Fg-milRNA-12 | 0 | 0 | 1 | 0 | 0 | 0 | 0 | 1 | 0 | 0 | 0 | 0 | 1 | 1 | 0 | 0 | 0 | 0 | 0 | 0 | 0 | 0 | 0 | 0 |
|  |  | Total | 1 | 1 | 3 | 0 | 0 | 0 | 0 | 1 | 2 | 0 | 0 | 0 | 1 | 2 | 1 | 0 | 0 | 0 | 1 | 0 | 0 | 0 | 0 | 0 |
| GACAACGTGGCCGAGTGG | 18 | Fg-milRNA-17 | 0 | 1 | 0 | 1 | 0 | 0 | 0 | 1 | 1 | 0 | 0 | 0 | 2 | 0 | 0 | 0 | 0 | 0 | 0 | 1 | 0 | 0 | 0 | 0 |
|  |  |  | 0 | 1 | 0 | 1 | 0 | 0 | 0 | 1 | 1 | 0 | 0 | 0 | 2 | 0 | 0 | 0 | 0 | 0 | 0 | 1 | 0 | 0 | 0 | 0 |
| AATGTTGACCTCGGATCA | 18 | Fg-milRNA-2 | 0 | 0 | 0 | 0 | 0 | 0 | 0 | 0 | 0 | 0 | 0 | 0 | 0 | 0 | 0 | 0 | 0 | 0 | 0 | 0 | 1 | 0 | 0 | 0 |
| AATGTTGACCTCGGATCAG | 19 | Fg-milRNA-2 | 1 | 0 | 0 | 0 | 0 | 0 | 0 | 0 | 0 | 0 | 0 | 0 | 0 | 0 | 0 | 0 | 0 | 0 | 0 | 0 | 0 | 0 | 0 | 0 |
| ATGTTGACCTCGGATCAGG | 19 | Fg-milRNA-2 | 1 | 0 | 0 | 0 | 0 | 0 | 0 | 0 | 0 | 0 | 0 | 0 | 0 | 0 | 0 | 0 | 0 | 0 | 0 | 0 | 0 | 0 | 0 | 0 |
| GAATGTTGACCTCGGATC | 18 | Fg-milRNA-2 | 5 | 1 | 1 | 0 | 0 | 0 | 2 | 4 | 2 | 0 | 0 | 0 | 3 | 2 | 4 | 0 | 0 | 0 | 2 | 4 | 3 | 0 | 0 | 0 |
| GAATGTTGACCTCGGATCA | 19 | Fg-milRNA-2 | 5 | 1 | 0 | 0 | 0 | 0 | 3 | 2 | 0 | 0 | 1 | 0 | 0 | 0 | 0 | 0 | 0 | 0 | 2 | 2 | 3 | 0 | 0 | 0 |
| GAATGTTGACCTCGGATCAG | 20 | Fg-milRNA-2 | 0 | 0 | 0 | 0 | 0 | 0 | 1 | 2 | 0 | 0 | 0 | 0 | 0 | 2 | 2 | 0 | 0 | 0 | 0 | 1 | 0 | 0 | 0 | 0 |
| GAATGTTGACCTCGGATCAGG | 21 | Fg-milRNA-2 | 0 | 0 | 1 | 0 | 0 | 0 | 0 | 0 | 0 | 0 | 0 | 0 | 0 | 0 | 0 | 0 | 0 | 0 | 0 | 0 | 1 | 0 | 0 | 0 |
| TGTTGACCTCGGATCAGG | 18 | Fg-milRNA-2 | 1 | 0 | 0 | 0 | 0 | 0 | 0 | 0 | 0 | 0 | 0 | 0 | 0 | 0 | 0 | 0 | 0 | 0 | 0 | 0 | 0 | 0 | 0 | 0 |
|  |  | Total | 13 | 2 | 2 | 0 | 0 | 0 | 6 | 8 | 2 | 0 | 1 | 0 | 3 | 4 | 6 | 0 | 0 | 0 | 4 | 7 | 8 | 0 | 0 | 0 |
| GGTACTGTGGTCTAGTTG | 18 | Fg-milRNA-3 | 1 | 0 | 1 | 0 | 0 | 0 | 1 | 1 | 0 | 0 | 0 | 0 | 2 | 0 | 0 | 0 | 0 | 0 | 0 | 1 | 0 | 0 | 0 | 0 |
| GGTACTGTGGTCTAGTTGG | 19 | Fg-milRNA-3 | 0 | 0 | 0 | 0 | 0 | 0 | 1 | 1 | 0 | 0 | 0 | 0 | 0 | 1 | 0 | 0 | 0 | 0 | 0 | 0 | 0 | 0 | 0 | 0 |
| GTACTGTGGTCTAGTTGG | 18 | Fg-milRNA-3 | 0 | 0 | 0 | 0 | 0 | 0 | 0 | 0 | 1 | 0 | 0 | 0 | 0 | 0 | 0 | 0 | 0 | 0 | 0 | 0 | 0 | 0 | 0 | 0 |
|  |  | Total | 1 | 0 | 1 | 0 | 0 | 0 | 2 | 2 | 1 | 0 | 0 | 0 | 2 | 1 | 0 |  |  |  |  | 0 | 0 | 0 | 0 | 0 |
| ACATACGACCATACCTAC | 18 | Fg-milRNA-31 | 1 | 0 | 1 | 0 | 0 | 0 | 2 | 0 | 0 | 0 | 0 | 0 | 1 | 2 | 0 | 0 | 0 | 0 | 0 | 0 | 1 | 0 | 0 | 0 |
| ACATACGACCATACCTACC | 19 | Fg-milRNA-31 | 9 | 4 | 2 | 0 | 0 | 0 | 8 | 5 | 2 | 0 | 1 | 0 | 6 | 2 | 3 | 0 | 0 | 0 | 0 | 0 | 1 | 0 | 0 | 0 |
| ACATACGACCATACCTACCA | 20 | Fg-milRNA-31 | 7 | 3 | 1 | 0 | 0 | 0 | 2 | 1 | 0 | 0 | 0 | 0 | 2 | 1 | 0 | 0 | 0 | 0 | 2 | 1 | 1 | 0 | 0 | 0 |
| ACATACGACCATACCTACCAG | 21 | Fg-milRNA-31 | 3 | 2 | 6 | 0 | 0 | 0 | 4 | 1 | 3 | 0 | 0 | 0 | 2 | 2 | 3 | 0 | 0 | 0 | 3 | 1 | 0 | 0 | 0 | 0 |
| ACATACGACCATACCTACCAGA | 22 | Fg-milRNA-31 | 10 | 11 | 16 | 1 | 0 | 0 | 11 | 6 | 4 | 0 | 0 | 0 | 6 | 3 | 6 | 0 | 0 | 0 | 5 | 8 | 2 | 0 | 0 | 0 |
| ACGACCATACCTACCAGA | 18 | Fg-milRNA-31 | 0 | 0 | 1 | 0 | 0 | 0 | 2 | 0 | 0 | 0 | 0 | 0 | 1 | 0 | 0 | 0 | 0 | 0 | 0 | 0 | 1 | 0 | 0 | 0 |
| ATACGACCATACCTACCA | 18 | Fg-milRNA-31 | 1 | 0 | 0 | 0 | 0 | 0 | 0 | 0 | 0 | 0 | 0 | 0 | 0 | 0 | 1 | 0 | 0 | 0 | 0 | 0 | 1 | 0 | 0 | 0 |
| ATACGACCATACCTACCAG | 19 | Fg-milRNA-31 | 1 | 0 | 0 | 0 | 0 | 0 | 1 | 0 | 0 | 0 | 0 | 0 | 0 | 0 | 0 | 0 | 0 | 0 | 0 | 1 | 0 | 0 | 0 | 0 |
| ATACGACCATACCTACCAGA | 20 | Fg-milRNA-31 | 1 | 0 | 2 | 0 | 0 | 0 | 1 | 0 | 0 | 0 | 0 | 0 | 0 | 0 | 1 | 0 | 0 | 0 | 0 | 2 | 2 | 0 | 0 | 0 |
| CATACGACCATACCTACC | 18 | Fg-milRNA-31 | 5 | 5 | 4 | 0 | 0 | 0 | 2 | 3 | 1 | 0 | 0 | 0 | 1 | 0 | 2 | 0 | 0 | 0 | 1 | 1 | 1 | 0 | 0 | 0 |
| CATACGACCATACCTACCA | 19 | Fg-milRNA-31 | 4 | 3 | 1 | 0 | 0 | 0 | 2 | 0 | 0 | 0 | 0 | 0 | 1 | 1 | 1 | 0 | 0 | 0 | 1 | 0 | 0 | 0 | 0 | 0 |
| CATACGACCATACCTACCAG | 20 | Fg-milRNA-31 | 1 | 1 | 1 | 0 | 0 | 0 | 3 | 1 | 0 | 0 | 0 | 0 | 2 | 1 | 1 | 0 | 0 | 0 | 0 | 1 | 2 | 0 | 0 | 0 |
| CATACGACCATACCTACCAGA | 21 | Fg-milRNA-31 | 1 | 1 | 3 | 0 | 0 | 0 | 8 | 1 | 2 | 0 | 0 | 0 | 1 | 1 | 7 | 0 | 0 | 0 | 1 | 2 | 1 | 0 | 0 | 0 |
| TACGACCATACCTACCAG | 18 | Fg-milRNA-31 | 1 | 0 | 1 | 0 | 0 | 0 | 0 | 0 | 1 | 0 | 0 | 0 | 0 | 0 | 0 | 0 | 0 | 0 | 1 | 0 | 0 | 0 | 0 | 0 |
| TACGACCATACCTACCAGA | 19 | Fg-milRNA-31 | 0 | 2 | 1 | 0 | 0 | 0 | 2 | 0 | 1 | 0 | 0 | 0 | 2 | 0 | 3 | 0 | 0 | 0 | 0 | 2 | 0 | 0 | 0 | 0 |
|  |  | Total | 45 | 32 | 40 | 1 | 0 | 0 | 48 | 18 | 14 | 0 | 1 | 0 | 25 | 13 | 28 | 0 | 0 | 0 | 14 | 19 | 13 | 0 | 0 | 0 |
| CGGGTTTAGCTCAGTTGGG | 19 | Fg-milRNA-34 | 0 | 0 | 0 | 0 | 0 | 0 | 0 | 0 | 0 | 0 | 0 | 0 | 0 | 0 | 2 | 0 | 0 | 0 | 0 | 0 | 0 | 0 | 0 | 0 |
| CGGGTTTAGCTCAGTTGGGA | 20 | Fg-milRNA-34 | 5 | 1 | 4 | 0 | 0 | 0 | 0 | 0 | 1 | 0 | 0 | 0 | 0 | 1 | 7 | 0 | 0 | 0 | 0 | 2 | 0 | 0 | 0 | 0 |
| CGGGTTTAGCTCAGTTGGGAG | 21 | Fg-milRNA-34 | 1 | 0 | 0 | 0 | 0 | 0 | 1 | 0 | 0 | 0 | 0 | 0 | 0 | 1 | 1 | 0 | 0 | 0 | 0 | 0 | 0 | 0 | 0 | 0 |
| CGGGTTTAGCTCAGTTGGGAGA | 22 | Fg-milRNA-34 | 3 | 0 | 1 | 0 | 0 | 0 | 2 | 0 | 0 | 0 | 0 | 0 | 1 | 0 | 1 | 0 | 0 | 0 | 0 | 1 | 1 | 0 | 0 | 0 |
| GCGGGTTTAGCTCAGTTG | 18 | Fg-milRNA-34 | 0 | 2 | 0 | 0 | 0 | 0 | 1 | 0 | 0 | 0 | 0 | 0 | 0 | 0 | 0 | 0 | 0 | 0 | 0 | 0 | 0 | 0 | 0 | 0 |
| GCGGGTTTAGCTCAGTTGG | 19 | Fg-milRNA-34 | 0 | 1 | 0 | 0 | 0 | 0 | 1 | 0 | 0 | 0 | 0 | 0 | 0 | 0 | 0 | 0 | 0 | 0 | 0 | 1 | 0 | 0 | 0 | 0 |
| GCGGGTTTAGCTCAGTTGGG | 20 | Fg-milRNA-34 | 1 | 3 | 4 | 0 | 1 | 1 | 3 | 2 | 6 | 0 | 0 | 0 | 2 | 0 | 0 | 0 | 0 | 0 | 3 | 0 | 0 | 0 | 0 | 0 |
| GCGGGTTTAGCTCAGTTGGGA | 21 | Fg-milRNA-34 | 10 | 4 | 2 | 0 | 0 | 0 | 1 | 1 | 4 | 0 | 0 | 0 | 0 | 0 | 5 | 1 | 0 | 0 | 1 | 2 | 0 | 0 | 0 | 0 |
| GCGGGTTTAGCTCAGTTGGGAG | 22 | Fg-milRNA-34 | 13 | 3 | 6 | 0 | 1 | 0 | 13 | 9 | 10 | 1 | 0 | 0 | 4 | 2 | 6 | 0 | 0 | 0 | 3 | 4 | 2 | 0 | 0 | 0 |
| GCGGGTTTAGCTCAGTTGGGAGA | 23 | Fg-milRNA-34 | 10 | 3 | 2 | 0 | 0 | 0 | 8 | 8 | 1 | 0 | 0 | 0 | 4 | 3 | 4 | 0 | 0 | 0 | 6 | 6 | 4 | 0 | 0 | 0 |
| GGGTTTAGCTCAGTTGGG | 18 | Fg-milRNA-34 | 0 | 0 | 0 | 0 | 0 | 0 | 0 | 0 | 0 | 0 | 0 | 0 | 0 | 0 | 0 | 0 | 0 | 0 | 0 | 1 | 0 | 0 | 0 | 0 |
| GGGTTTAGCTCAGTTGGGA | 19 | Fg-milRNA-34 | 0 | 0 | 0 | 0 | 0 | 0 | 0 | 0 | 0 | 0 | 0 | 0 | 0 | 0 | 1 | 0 | 0 | 0 | 0 | 0 | 0 | 0 | 0 | 0 |
| GGGTTTAGCTCAGTTGGGAG | 20 | Fg-milRNA-34 | 0 | 0 | 0 | 0 | 0 | 0 | 0 | 0 | 1 | 0 | 0 | 0 | 0 | 0 | 0 | 0 | 0 | 0 | 0 | 0 | 0 | 0 | 0 | 0 |
|  |  | Total | 43 | 17 | 19 | 0 | 2 | 1 | 30 | 20 | 23 | 1 | 0 | 0 | 11 | 7 | 27 | 1 | 0 | 0 | 13 | 17 | 7 | 0 | 0 | 0 |
| GGTGAGATGGCCGAGTTG | 18 | Fg-milRNA-35 | 8 | 1 | 5 | 0 | 0 | 0 | 7 | 2 | 4 | 0 | 0 | 0 | 0 | 5 | 2 | 0 | 0 | 0 | 2 | 2 | 2 | 0 | 0 | 0 |
| GGTGAGATGGCCGAGTTGG | 19 | Fg-milRNA-35 | 8 | 0 | 2 | 0 | 0 | 0 | 2 | 2 | 4 | 0 | 0 | 0 | 1 | 1 | 4 | 0 | 0 | 0 | 0 | 5 | 0 | 0 | 0 | 0 |
| GGTGAGATGGCCGAGTTGGT | 20 | Fg-milRNA-35 | 0 | 0 | 0 | 0 | 0 | 0 | 0 | 0 | 0 | 0 | 0 | 0 | 0 | 0 | 0 | 0 | 0 | 0 | 1 | 0 | 0 | 0 | 0 | 0 |
| GTGAGATGGCCGAGTTGG | 18 | Fg-milRNA-35 | 1 | 2 | 0 | 0 | 0 | 0 | 0 | 0 | 0 | 0 | 0 | 0 | 0 | 1 | 0 | 0 | 0 | 0 | 0 | 0 | 0 | 0 | 0 | 0 |
|  |  | Total | 17 | 3 | 7 | 0 | 0 | 0 | 9 | 4 | 8 | 0 | 0 | 0 | 1 | 7 | 6 | 0 | 0 | 0 | 3 | 7 | 2 | 0 | 0 | 0 |
| CTGGATCATTGTAGACCAGGC | 21 | Fg-milRNA-41 | 0 | 0 | 0 | 0 | 0 | 0 | 0 | 1 | 0 | 0 | 0 | 0 | 0 | 0 | 0 | 0 | 0 | 0 | 0 | 0 | 0 | 0 | 0 | 0 |
|  |  | Total | 0 | 0 | 0 | 0 | 0 | 0 | 0 | 1 | 0 | 0 | 0 | 0 | 0 | 0 | 0 | 0 | 0 | 0 | 0 | 0 | 0 | 0 | 0 | 0 |
| GGCAACGTGACGGAGTGG | 18 | Fg-milRNA-49 | 0 | 0 | 1 | 0 | 0 | 0 | 0 | 0 | 0 | 0 | 0 | 0 | 0 | 0 | 0 | 0 | 0 | 0 | 0 | 0 | 0 | 0 | 0 | 0 |
| GGCAACGTGACGGAGTGGT | 19 | Fg-milRNA-49 | 1 | 0 | 0 | 0 | 0 | 0 | 0 | 0 | 0 | 0 | 0 | 0 | 0 | 0 | 0 | 0 | 0 | 0 | 0 | 0 | 0 | 0 | 0 | 0 |
| GGCAACGTGACGGAGTGGTTA | 21 | Fg-milRNA-49 | 0 | 0 | 0 | 0 | 0 | 0 | 0 | 0 | 0 | 0 | 0 | 0 | 0 | 0 | 1 | 0 | 0 | 0 | 1 | 0 | 0 | 0 | 0 | 0 |
|  |  | Total | 1 | 0 | 1 | 0 | 0 | 0 | 0 | 0 | 0 | 0 | 0 | 0 | 0 | 0 | 1 | 0 | 0 | 0 | 1 | 0 | 0 | 0 | 0 | 0 |
| TTATATATTTTTTGACTGTCA | 21 | Fg-milRNA-6 | 0 | 0 | 0 | 0 | 0 | 0 | 0 | 0 | 0 | 0 | 0 | 0 | 0 | 0 | 1 | 0 | 0 | 0 | 0 | 0 | 0 | 0 | 0 | 0 |
|  |  | Total | 0 | 0 | 0 | 0 | 0 | 0 | 0 | 0 | 0 | 0 | 0 | 0 | 0 | 0 | 1 | 0 | 0 | 0 | 0 | 0 | 0 | 0 | 0 | 0 |
| TAAACTGAGAAGATTAGGGC | 20 | Fg-milRNA-7 | 0 | 0 | 0 | 0 | 0 | 0 | 0 | 0 | 0 | 0 | 0 | 0 | 0 | 1 | 0 | 0 | 0 | 0 | 0 | 0 | 1 | 1 | 0 | 0 |
| TAAACTGAGAAGATTAGGGCT | 21 | Fg-milRNA-7 | 0 | 0 | 0 | 0 | 0 | 0 | 0 | 1 | 0 | 0 | 0 | 0 | 2 | 2 | 1 | 0 | 0 | 0 | 0 | 2 | 1 | 0 | 0 | 0 |
|  |  | Total | 0 | 0 | 0 | 0 | 0 | 0 | 0 | 1 | 0 | 0 | 0 | 0 | 2 | 3 | 1 | 0 | 0 | 0 | 0 | 2 | 2 | 1 | 0 | 0 |
| AACGTGGCCGAGTGGTTAAG | 20 | fox_milRNA_2a//fox_milRNA_2b | 0 | 0 | 0 | 0 | 0 | 0 | 0 | 0 | 0 | 0 | 0 | 0 | 0 | 0 | 0 | 0 | 0 | 0 | 1 | 0 | 0 | 0 | 0 | 0 |
| AACGTGGCCGAGTGGTTAAGG | 21 | fox_milRNA_2a//fox_milRNA_2b | 1 | 0 | 0 | 0 | 0 | 0 | 0 | 0 | 0 | 0 | 0 | 0 | 0 | 0 | 0 | 0 | 0 | 0 | 0 | 0 | 0 | 0 | 0 | 0 |
| AACGTGGCCGAGTGGTTAAGGC | 22 | fox_milRNA_2a//fox_milRNA_2b | 0 | 0 | 1 | 0 | 0 | 0 | 0 | 1 | 0 | 0 | 0 | 0 | 0 | 0 | 0 | 0 | 0 | 0 | 0 | 0 | 0 | 0 | 0 | 0 |
| ACAACGTGGCCGAGTGGTTAAG | 22 | fox_milRNA_2a//fox_milRNA_2b | 0 | 0 | 0 | 0 | 0 | 0 | 0 | 0 | 0 | 0 | 0 | 0 | 1 | 0 | 0 | 0 | 0 | 0 | 0 | 0 | 0 | 0 | 0 | 0 |
| ACAACGTGGCCGAGTGGTTAAGG | 23 | fox_milRNA_2a//fox_milRNA_2b | 0 | 0 | 1 | 0 | 0 | 0 | 0 | 0 | 1 | 0 | 0 | 0 | 1 | 0 | 0 | 0 | 0 | 0 | 0 | 0 | 0 | 0 | 0 | 0 |
| ACAACGTGGCCGAGTGGTTAAGGC | 24 | fox_milRNA_2a//fox_milRNA_2b | 0 | 0 | 0 | 0 | 0 | 0 | 0 | 0 | 0 | 0 | 0 | 0 | 0 | 0 | 0 | 0 | 0 | 0 | 1 | 0 | 0 | 0 | 0 | 0 |
| ACGTGGCCGAGTGGTTAAG | 19 | fox_milRNA_2a//fox_milRNA_2b | 1 | 0 | 0 | 0 | 0 | 0 | 0 | 0 | 0 | 0 | 0 | 0 | 0 | 0 | 0 | 0 | 0 | 0 | 0 | 0 | 0 | 0 | 0 | 0 |
| ACGTGGCCGAGTGGTTAAGGC | 21 | fox_milRNA_2a//fox_milRNA_2b | 0 | 0 | 0 | 0 | 0 | 0 | 1 | 0 | 0 | 0 | 0 | 0 | 0 | 0 | 0 | 0 | 0 | 0 | 0 | 0 | 0 | 0 | 0 | 0 |
| CGTGGCCGAGTGGTTAAG | 18 | fox_milRNA_2a//fox_milRNA_2b | 1 | 0 | 0 | 0 | 0 | 0 | 0 | 0 | 0 | 0 | 0 | 0 | 0 | 0 | 0 | 0 | 0 | 0 | 0 | 0 | 0 | 0 | 0 | 0 |
| GACAACGTGGCCGAGTGGTTA | 21 | fox_milRNA_2a//fox_milRNA_2b | 0 | 0 | 0 | 0 | 0 | 0 | 0 | 1 | 0 | 0 | 0 | 0 | 0 | 0 | 0 | 0 | 0 | 0 | 0 | 0 | 0 | 0 | 0 | 0 |
| GACAACGTGGCCGAGTGGTTAA | 22 | fox_milRNA_2a//fox_milRNA_2b | 2 | 0 | 0 | 0 | 0 | 0 | 2 | 0 | 0 | 0 | 0 | 0 | 0 | 0 | 0 | 0 | 0 | 0 | 0 | 0 | 0 | 0 | 0 | 0 |
| GACAACGTGGCCGAGTGGTTAAG | 23 | fox_milRNA_2a//fox_milRNA_2b | 4 | 0 | 3 | 0 | 0 | 0 | 7 | 0 | 2 | 0 | 0 | 0 | 0 | 1 | 1 | 0 | 0 | 0 | 0 | 0 | 1 | 0 | 0 | 0 |
| GACAACGTGGCCGAGTGGTTAAGG | 24 | fox_milRNA_2a//fox_milRNA_2b | 11 | 1 | 3 | 0 | 0 | 0 | 2 | 0 | 3 | 0 | 0 | 0 | 1 | 4 | 2 | 0 | 0 | 0 | 1 | 4 | 1 | 0 | 0 | 0 |
| GACAACGTGGCCGAGTGGTTAAGGC | 25 | fox_milRNA_2a//fox_milRNA_2b | 1 | 1 | 1 | 0 | 0 | 0 | 1 | 3 | 0 | 0 | 0 | 0 | 0 | 0 | 0 | 0 | 0 | 0 | 1 | 3 | 0 | 0 | 0 | 0 |
| GTGGCCGAGTGGTTAAGG | 18 | fox_milRNA_2a//fox_milRNA_2b | 0 | 0 | 1 | 0 | 0 | 0 | 0 | 0 | 0 | 0 | 0 | 0 | 0 | 0 | 0 | 0 | 0 | 0 | 0 | 0 | 0 | 0 | 0 | 0 |
| TGGCCGAGTGGTTAAGGC | 18 | fox_milRNA_2a//fox_milRNA_2b | 0 | 0 | 0 | 0 | 0 | 0 | 1 | 0 | 0 | 0 | 0 | 0 | 0 | 0 | 0 | 1 | 0 | 0 | 0 | 0 | 0 | 0 | 0 | 0 |
|  |  | Total | 21 | 2 | 10 | 0 | 0 | 0 | 14 | 5 | 6 | 0 | 0 | 0 | 3 | 5 | 3 | 1 | 0 | 0 | 4 | 7 | 2 | 0 | 0 | 0 |
| ATTGTGTTCGCACGCGTAG | 19 | fox_milRNA_2c//fox_milRNA_2d//fox_milRNA_2e | 1 | 0 | 0 | 0 | 0 | 0 | 0 | 0 | 0 | 0 | 0 | 0 | 0 | 0 | 1 | 0 | 0 | 0 | 0 | 0 | 0 | 0 | 0 | 0 |
| ATTGTGTTCGCACGCGTAGG | 20 | fox_milRNA_2c//fox_milRNA_2d//fox_milRNA_2e | 0 | 0 | 0 | 0 | 0 | 0 | 1 | 0 | 0 | 0 | 0 | 0 | 0 | 0 | 0 | 0 | 0 | 0 | 0 | 0 | 0 | 0 | 0 | 0 |
| ATTGTGTTCGCACGCGTAGGT | 21 | fox_milRNA_2c//fox_milRNA_2d//fox_milRNA_2e | 0 | 1 | 0 | 0 | 0 | 0 | 0 | 0 | 0 | 0 | 0 | 0 | 0 | 0 | 0 | 0 | 0 | 0 | 0 | 0 | 0 | 0 | 0 | 0 |
| ATTGTGTTCGCACGCGTAGGTT | 22 | fox_milRNA_2c//fox_milRNA_2d//fox_milRNA_2e | 2 | 0 | 1 | 0 | 0 | 0 | 1 | 0 | 2 | 0 | 0 | 0 | 1 | 0 | 0 | 0 | 0 | 0 | 0 | 0 | 0 | 0 | 0 | 0 |
| ATTGTGTTCGCACGCGTAGGTTC | 23 | fox_milRNA_2c//fox_milRNA_2d//fox_milRNA_2e | 6 | 4 | 5 | 0 | 0 | 0 | 5 | 3 | 1 | 0 | 0 | 0 | 0 | 1 | 3 | 0 | 0 | 0 | 3 | 1 | 1 | 0 | 0 | 0 |
| ATTGTGTTCGCACGCGTAGGTTCG | 24 | fox_milRNA_2c//fox_milRNA_2d//fox_milRNA_2e | 1 | 1 | 2 | 0 | 0 | 0 | 0 | 1 | 1 | 0 | 0 | 0 | 2 | 0 | 1 | 0 | 0 | 0 | 1 | 0 | 0 | 0 | 0 | 0 |
| CATTGTGTTCGCACGCGT | 18 | fox_milRNA_2c//fox_milRNA_2d//fox_milRNA_2e | 0 | 0 | 1 | 0 | 0 | 0 | 0 | 0 | 0 | 0 | 0 | 0 | 0 | 0 | 0 | 0 | 0 | 0 | 0 | 0 | 0 | 0 | 0 | 0 |
| CATTGTGTTCGCACGCGTA | 19 | fox_milRNA_2c//fox_milRNA_2d//fox_milRNA_2e | 4 | 0 | 0 | 0 | 0 | 0 | 0 | 1 | 0 | 0 | 0 | 0 | 0 | 0 | 0 | 0 | 0 | 0 | 0 | 0 | 0 | 0 | 0 | 0 |
| CATTGTGTTCGCACGCGTAGGT | 22 | fox_milRNA_2c//fox_milRNA_2d//fox_milRNA_2e | 2 | 0 | 0 | 0 | 0 | 0 | 1 | 0 | 1 | 0 | 0 | 0 | 0 | 0 | 0 | 0 | 0 | 0 | 0 | 0 | 0 | 0 | 0 | 0 |
| CATTGTGTTCGCACGCGTAGGTT | 23 | fox_milRNA_2c//fox_milRNA_2d//fox_milRNA_2e | 2 | 0 | 0 | 0 | 0 | 0 | 2 | 0 | 0 | 0 | 0 | 0 | 1 | 0 | 1 | 0 | 0 | 0 | 0 | 1 | 1 | 0 | 0 | 0 |
| CATTGTGTTCGCACGCGTAGGTTC | 24 | fox_milRNA_2c//fox_milRNA_2d//fox_milRNA_2e | 10 | 5 | 7 | 0 | 0 | 0 | 10 | 1 | 0 | 0 | 0 | 0 | 1 | 4 | 8 | 0 | 0 | 0 | 1 | 2 | 0 | 0 | 0 | 0 |
| CATTGTGTTCGCACGCGTAGGTTCG | 25 | fox_milRNA_2c//fox_milRNA_2d//fox_milRNA_2e | 1 | 3 | 6 | 0 | 0 | 0 | 4 | 1 | 1 | 0 | 0 | 0 | 1 | 2 | 2 | 0 | 0 | 0 | 3 | 2 | 1 | 0 | 0 | 0 |
| GCATTGTGTTCGCACGCG | 18 | fox_milRNA_2c//fox_milRNA_2d//fox_milRNA_2e | 0 | 0 | 0 | 0 | 0 | 0 | 1 | 1 | 0 | 0 | 0 | 0 | 0 | 0 | 0 | 0 | 0 | 0 | 0 | 0 | 0 | 0 | 0 | 0 |
| GCATTGTGTTCGCACGCGTAGGTTC | 25 | fox_milRNA_2c//fox_milRNA_2d//fox_milRNA_2e | 2 | 0 | 0 | 0 | 0 | 0 | 1 | 0 | 1 | 0 | 0 | 0 | 0 | 0 | 0 | 0 | 0 | 0 | 0 | 0 | 1 | 0 | 0 | 0 |
| GCATTGTGTTCGCACGCGTAGGTTCG | 26 | fox_milRNA_2c//fox_milRNA_2d//fox_milRNA_2e | 0 | 0 | 0 | 0 | 0 | 0 | 0 | 0 | 0 | 0 | 0 | 0 | 0 | 0 | 0 | 0 | 0 | 0 | 0 | 1 | 0 | 0 | 0 | 0 |
| GGCATTGTGTTCGCACGC | 18 | fox_milRNA_2c//fox_milRNA_2d//fox_milRNA_2e | 1 | 2 | 2 | 0 | 0 | 0 | 1 | 0 | 0 | 0 | 0 | 0 | 0 | 0 | 1 | 0 | 0 | 0 | 0 | 0 | 0 | 0 | 0 | 0 |
| GGCATTGTGTTCGCACGCG | 19 | fox_milRNA_2c//fox_milRNA_2d//fox_milRNA_2e | 14 | 7 | 8 | 0 | 0 | 0 | 5 | 1 | 4 | 0 | 0 | 0 | 1 | 4 | 8 | 0 | 0 | 0 | 0 | 2 | 0 | 0 | 0 | 0 |
| GGCATTGTGTTCGCACGCGTA | 21 | fox_milRNA_2c//fox_milRNA_2d//fox_milRNA_2e | 0 | 1 | 0 | 0 | 0 | 0 | 1 | 0 | 0 | 0 | 0 | 0 | 0 | 0 | 0 | 0 | 0 | 0 | 1 | 0 | 0 | 0 | 0 | 0 |
| GGCATTGTGTTCGCACGCGTAG | 22 | fox_milRNA_2c//fox_milRNA_2d//fox_milRNA_2e | 0 | 0 | 0 | 0 | 0 | 0 | 0 | 1 | 0 | 0 | 0 | 0 | 0 | 0 | 0 | 0 | 0 | 0 | 0 | 0 | 0 | 0 | 0 | 0 |
| GGCATTGTGTTCGCACGCGTAGGT | 24 | fox_milRNA_2c//fox_milRNA_2d//fox_milRNA_2e | 0 | 0 | 0 | 0 | 0 | 0 | 0 | 1 | 0 | 0 | 0 | 0 | 0 | 1 | 1 | 0 | 0 | 0 | 0 | 0 | 0 | 0 | 0 | 0 |
| GGCATTGTGTTCGCACGCGTAGGTT | 25 | fox_milRNA_2c//fox_milRNA_2d//fox_milRNA_2e | 5 | 0 | 2 | 0 | 0 | 0 | 0 | 1 | 0 | 0 | 0 | 0 | 1 | 1 | 0 | 0 | 0 | 0 | 0 | 0 | 0 | 0 | 0 | 0 |
| GGCATTGTGTTCGCACGCGTAGGTTC | 26 | fox_milRNA_2c//fox_milRNA_2d//fox_milRNA_2e | 51 | 21 | 33 | 0 | 0 | 0 | 26 | 3 | 4 | 0 | 0 | 0 | 10 | 7 | 35 | 0 | 0 | 0 | 1 | 5 | 0 | 0 | 0 | 0 |
| GGCATTGTGTTCGCACGCGTAGGTTCG | 27 | fox_milRNA_2c//fox_milRNA_2d//fox_milRNA_2e | 1 | 0 | 0 | 0 | 0 | 0 | 2 | 0 | 0 | 0 | 0 | 0 | 0 | 0 | 0 | 0 | 0 | 0 | 0 | 0 | 0 | 0 | 0 | 0 |
| GTGTTCGCACGCGTAGGTTC | 20 | fox_milRNA_2c//fox_milRNA_2d//fox_milRNA_2e | 2 | 0 | 0 | 0 | 0 | 0 | 1 | 0 | 1 | 0 | 0 | 0 | 2 | 0 | 0 | 0 | 0 | 0 | 0 | 0 | 1 | 0 | 0 | 0 |
| GTGTTCGCACGCGTAGGTTCG | 21 | fox_milRNA_2c//fox_milRNA_2d//fox_milRNA_2e | 3 | 0 | 0 | 0 | 0 | 0 | 0 | 1 | 0 | 0 | 0 | 0 | 0 | 0 | 0 | 0 | 0 | 0 | 0 | 0 | 0 | 0 | 0 | 0 |
| GTTCGCACGCGTAGGTTC | 18 | fox_milRNA_2c//fox_milRNA_2d//fox_milRNA_2e | 5 | 3 | 5 | 0 | 0 | 0 | 6 | 1 | 0 | 1 | 0 | 0 | 1 | 1 | 0 | 0 | 0 | 0 | 0 | 2 | 1 | 0 | 0 | 0 |
| GTTCGCACGCGTAGGTTCG | 19 | fox_milRNA_2c//fox_milRNA_2d//fox_milRNA_2e | 6 | 1 | 3 | 0 | 0 | 0 | 2 | 1 | 2 | 0 | 0 | 0 | 0 | 4 | 1 | 0 | 0 | 0 | 2 | 1 | 0 | 0 | 0 | 0 |
| TGTGTTCGCACGCGTAGGT | 19 | fox_milRNA_2c//fox_milRNA_2d//fox_milRNA_2e | 0 | 0 | 1 | 0 | 0 | 0 | 1 | 0 | 0 | 0 | 0 | 0 | 0 | 0 | 0 | 0 | 0 | 0 | 0 | 1 | 0 | 0 | 0 | 0 |
| TGTGTTCGCACGCGTAGGTT | 20 | fox_milRNA_2c//fox_milRNA_2d//fox_milRNA_2e | 1 | 0 | 1 | 0 | 0 | 0 | 0 | 0 | 0 | 0 | 0 | 0 | 0 | 0 | 0 | 0 | 0 | 0 | 0 | 0 | 0 | 0 | 0 | 0 |
| TGTGTTCGCACGCGTAGGTTC | 21 | fox_milRNA_2c//fox_milRNA_2d//fox_milRNA_2e | 14 | 11 | 14 | 0 | 0 | 0 | 14 | 2 | 2 | 0 | 0 | 0 | 3 | 10 | 13 | 0 | 0 | 0 | 2 | 3 | 3 | 0 | 0 | 0 |
| TGTGTTCGCACGCGTAGGTTCG | 22 | fox_milRNA_2c//fox_milRNA_2d//fox_milRNA_2e | 6 | 2 | 0 | 0 | 0 | 0 | 3 | 0 | 1 | 0 | 0 | 0 | 0 | 0 | 2 | 0 | 0 | 0 | 0 | 0 | 0 | 0 | 0 | 0 |
| TGTTCGCACGCGTAGGTT | 18 | fox_milRNA_2c//fox_milRNA_2d//fox_milRNA_2e | 0 | 1 | 0 | 0 | 0 | 0 | 2 | 0 | 0 | 0 | 0 | 0 | 1 | 0 | 0 | 0 | 0 | 0 | 0 | 0 | 0 | 0 | 0 | 0 |
| TGTTCGCACGCGTAGGTTC | 19 | fox_milRNA_2c//fox_milRNA_2d//fox_milRNA_2e | 18 | 7 | 8 | 0 | 0 | 0 | 9 | 3 | 5 | 0 | 0 | 0 | 6 | 5 | 10 | 0 | 0 | 0 | 3 | 2 | 2 | 0 | 0 | 0 |
| TGTTCGCACGCGTAGGTTCG | 20 | fox_milRNA_2c//fox_milRNA_2d//fox_milRNA_2e | 2 | 0 | 0 | 0 | 0 | 0 | 0 | 0 | 0 | 0 | 0 | 0 | 0 | 0 | 0 | 0 | 0 | 0 | 0 | 0 | 0 | 0 | 0 | 0 |
| TTCGCACGCGTAGGTTCG | 18 | fox_milRNA_2c//fox_milRNA_2d//fox_milRNA_2e | 9 | 10 | 7 | 0 | 0 | 0 | 7 | 1 | 0 | 0 | 0 | 0 | 6 | 2 | 3 | 0 | 0 | 0 | 2 | 2 | 0 | 1 | 0 | 0 |
| TTGTGTTCGCACGCGTAGGT | 20 | fox_milRNA_2c//fox_milRNA_2d//fox_milRNA_2e | 2 | 0 | 0 | 0 | 0 | 0 | 1 | 0 | 0 | 0 | 0 | 0 | 0 | 0 | 0 | 0 | 0 | 0 | 0 | 0 | 0 | 0 | 0 | 0 |
| TTGTGTTCGCACGCGTAGGTT | 21 | fox_milRNA_2c//fox_milRNA_2d//fox_milRNA_2e | 0 | 0 | 0 | 0 | 0 | 0 | 2 | 0 | 2 | 0 | 0 | 0 | 1 | 0 | 0 | 0 | 0 | 0 | 1 | 0 | 0 | 0 | 0 | 0 |
| TTGTGTTCGCACGCGTAGGTTC | 22 | fox_milRNA_2c//fox_milRNA_2d//fox_milRNA_2e | 17 | 9 | 8 | 0 | 0 | 0 | 16 | 1 | 0 | 0 | 0 | 0 | 6 | 3 | 8 | 0 | 0 | 0 | 0 | 1 | 3 | 0 | 0 | 0 |
| TTGTGTTCGCACGCGTAGGTTCG | 23 | fox_milRNA_2c//fox_milRNA_2d//fox_milRNA_2e | 12 | 3 | 7 | 0 | 0 | 0 | 11 | 0 | 2 | 0 | 0 | 0 | 0 | 2 | 5 | 0 | 0 | 0 | 2 | 3 | 1 | 0 | 0 | 0 |
|  |  | Total | 200 | 92 | 121 | 0 | 0 | 0 | 136 | 25 | 30 | 1 | 0 | 0 | 44 | 47 | 103 | 0 | 0 | 0 | 22 | 29 | 15 | 1 | 0 | 0 |
| GTTCCGTGGTCTAGTTGGTTATGGCAT | 27 | fox_milRNA_6 | 0 | 0 | 0 | 0 | 0 | 0 | 0 | 0 | 0 | 0 | 0 | 0 | 1 | 0 | 0 | 0 | 0 | 0 | 0 | 0 | 0 | 0 | 0 | 0 |
| GTTCCGTGGTCTAGTTGGTTATGGCATCT | 29 | fox_milRNA_6 | 0 | 0 | 1 | 0 | 0 | 0 | 0 | 1 | 0 | 0 | 0 | 0 | 0 | 0 | 0 | 0 | 0 | 0 | 0 | 0 | 1 | 0 | 0 | 0 |
| TCCGTGGTCTAGTTGGTTATGGC | 23 | fox_milRNA_6 | 0 | 0 | 0 | 0 | 0 | 0 | 0 | 0 | 1 | 0 | 0 | 0 | 0 | 0 | 0 | 0 | 0 | 0 | 0 | 0 | 0 | 0 | 0 | 0 |
| TCTAGTTGGTTATGGCATCT | 20 | fox_milRNA_6 | 0 | 0 | 0 | 0 | 0 | 0 | 1 | 0 | 0 | 0 | 0 | 0 | 0 | 0 | 0 | 0 | 0 | 0 | 0 | 0 | 0 | 0 | 0 | 0 |
| TTCCGTGGTCTAGTTGGTTATGGCATCT | 28 | fox_milRNA_6 | 0 | 0 | 0 | 0 | 0 | 0 | 2 | 0 | 0 | 0 | 0 | 0 | 1 | 0 | 0 | 0 | 0 | 0 | 1 | 0 | 1 | 0 | 0 | 0 |
|  |  |  | 0 | 0 | 1 | 0 | 0 | 0 | 3 | 1 | 1 | 0 | 0 | 0 | 2 | 0 | 0 | 0 | 0 | 0 | 1 | 0 | 2 | 0 | 0 | 0 |
| AGTATAGTGGTCAGTATG | 18 | fox_milRNA_7 | 1 | 0 | 2 | 0 | 0 | 0 | 3 | 1 | 0 | 0 | 0 | 0 | 0 | 0 | 0 | 0 | 0 | 0 | 0 | 0 | 0 | 0 | 0 | 0 |
| AGTATAGTGGTCAGTATGC | 19 | fox_milRNA_7 | 0 | 0 | 0 | 0 | 0 | 0 | 0 | 0 | 0 | 0 | 0 | 0 | 0 | 0 | 0 | 0 | 0 | 0 | 0 | 1 | 0 | 0 | 0 | 0 |
| AGTATAGTGGTCAGTATGCA | 20 | fox_milRNA_7 | 1 | 0 | 0 | 0 | 0 | 0 | 0 | 0 | 0 | 0 | 0 | 0 | 0 | 0 | 0 | 0 | 0 | 0 | 0 | 0 | 0 | 0 | 0 | 0 |
| CCGTAGTATAGTGGTCAG | 18 | fox_milRNA_7 | 0 | 0 | 0 | 0 | 0 | 0 | 0 | 1 | 1 | 0 | 0 | 0 | 0 | 0 | 0 | 0 | 0 | 0 | 0 | 1 | 0 | 0 | 0 | 0 |
| CCGTAGTATAGTGGTCAGT | 19 | fox_milRNA_7 | 1 | 0 | 2 | 0 | 0 | 0 | 2 | 0 | 0 | 0 | 0 | 0 | 0 | 0 | 1 | 0 | 0 | 0 | 0 | 0 | 0 | 0 | 0 | 0 |
| CCGTAGTATAGTGGTCAGTA | 20 | fox_milRNA_7 | 1 | 0 | 0 | 0 | 0 | 0 | 0 | 2 | 0 | 0 | 0 | 0 | 0 | 0 | 0 | 0 | 0 | 0 | 0 | 1 | 0 | 0 | 0 | 1 |
| CCGTAGTATAGTGGTCAGTAT | 21 | fox_milRNA_7 | 0 | 1 | 1 | 0 | 0 | 0 | 1 | 0 | 0 | 0 | 0 | 0 | 0 | 1 | 0 | 0 | 0 | 0 | 0 | 1 | 0 | 0 | 0 | 0 |
| CCGTAGTATAGTGGTCAGTATG | 22 | fox_milRNA_7 | 22 | 14 | 15 | 0 | 0 | 0 | 25 | 30 | 20 | 0 | 0 | 0 | 13 | 11 | 26 | 0 | 0 | 1 | 30 | 53 | 31 | 0 | 0 | 0 |
| CCGTAGTATAGTGGTCAGTATGC | 23 | fox_milRNA_7 | 0 | 1 | 0 | 0 | 0 | 0 | 1 | 0 | 0 | 0 | 0 | 0 | 0 | 0 | 1 | 0 | 0 | 0 | 1 | 0 | 0 | 0 | 0 | 0 |
| CCGTAGTATAGTGGTCAGTATGCA | 24 | fox_milRNA_7 | 1 | 2 | 5 | 0 | 0 | 0 | 5 | 11 | 7 | 0 | 1 | 0 | 4 | 4 | 3 | 0 | 0 | 0 | 4 | 6 | 7 | 0 | 0 | 0 |
| CGTAGTATAGTGGTCAGT | 18 | fox_milRNA_7 | 1 | 1 | 0 | 0 | 0 | 0 | 1 | 1 | 0 | 0 | 0 | 0 | 0 | 0 | 0 | 0 | 0 | 0 | 0 | 0 | 0 | 0 | 0 | 0 |
| CGTAGTATAGTGGTCAGTA | 19 | fox_milRNA_7 | 1 | 0 | 0 | 0 | 0 | 0 | 1 | 0 | 0 | 0 | 0 | 0 | 0 | 0 | 0 | 0 | 0 | 0 | 0 | 0 | 0 | 0 | 0 | 0 |
| CGTAGTATAGTGGTCAGTAT | 20 | fox_milRNA_7 | 0 | 1 | 0 | 0 | 0 | 0 | 0 | 0 | 0 | 0 | 0 | 0 | 0 | 0 | 0 | 0 | 0 | 0 | 0 | 0 | 0 | 0 | 0 | 0 |
| CGTAGTATAGTGGTCAGTATG | 21 | fox_milRNA_7 | 1 | 0 | 2 | 0 | 0 | 0 | 2 | 1 | 1 | 0 | 0 | 0 | 3 | 1 | 3 | 0 | 0 | 0 | 1 | 0 | 0 | 0 | 0 | 0 |
| CGTAGTATAGTGGTCAGTATGCA | 23 | fox_milRNA_7 | 1 | 0 | 0 | 0 | 0 | 0 | 1 | 0 | 0 | 0 | 0 | 0 | 0 | 0 | 0 | 0 | 0 | 0 | 0 | 0 | 0 | 0 | 0 | 0 |
| CTTCCGTAGTATAGTGGTC | 19 | fox_milRNA_7 | 1 | 0 | 0 | 0 | 0 | 0 | 0 | 0 | 0 | 0 | 0 | 0 | 0 | 0 | 0 | 0 | 0 | 0 | 0 | 0 | 0 | 0 | 0 | 0 |
| CTTCCGTAGTATAGTGGTCA | 20 | fox_milRNA_7 | 4 | 0 | 1 | 0 | 0 | 0 | 0 | 0 | 0 | 0 | 0 | 0 | 1 | 1 | 2 | 0 | 0 | 0 | 0 | 0 | 0 | 0 | 0 | 0 |
| CTTCCGTAGTATAGTGGTCAG | 21 | fox_milRNA_7 | 7 | 1 | 2 | 0 | 0 | 0 | 1 | 1 | 0 | 0 | 0 | 0 | 1 | 1 | 3 | 0 | 0 | 0 | 1 | 2 | 1 | 0 | 0 | 0 |
| CTTCCGTAGTATAGTGGTCAGT | 22 | fox_milRNA_7 | 2 | 1 | 3 | 0 | 0 | 0 | 3 | 0 | 1 | 0 | 0 | 0 | 0 | 1 | 1 | 0 | 0 | 0 | 2 | 1 | 0 | 0 | 0 | 0 |
| CTTCCGTAGTATAGTGGTCAGTA | 23 | fox_milRNA_7 | 0 | 2 | 0 | 0 | 0 | 0 | 3 | 2 | 1 | 0 | 0 | 0 | 0 | 3 | 0 | 0 | 0 | 0 | 1 | 4 | 0 | 0 | 0 | 0 |
| CTTCCGTAGTATAGTGGTCAGTAT | 24 | fox_milRNA_7 | 1 | 1 | 2 | 0 | 0 | 0 | 2 | 1 | 2 | 0 | 0 | 0 | 2 | 0 | 0 | 0 | 0 | 0 | 0 | 2 | 0 | 0 | 0 | 0 |
| CTTCCGTAGTATAGTGGTCAGTATG | 25 | fox_milRNA_7 | 68 | 62 | 50 | 2 | 0 | 0 | 133 | 184 | 94 | 3 | 6 | 2 | 56 | 45 | 81 | 0 | 0 | 1 | 146 | 196 | 174 | 2 | 0 | 0 |
| CTTCCGTAGTATAGTGGTCAGTATGC | 26 | fox_milRNA_7 | 4 | 0 | 2 | 0 | 0 | 0 | 6 | 7 | 6 | 0 | 0 | 0 | 1 | 0 | 0 | 0 | 0 | 0 | 1 | 6 | 1 | 0 | 0 | 0 |
| CTTCCGTAGTATAGTGGTCAGTATGCA | 27 | fox_milRNA_7 | 18 | 13 | 7 | 0 | 0 | 0 | 32 | 10 | 26 | 0 | 1 | 0 | 5 | 2 | 12 | 0 | 0 | 0 | 4 | 19 | 23 | 0 | 0 | 0 |
| GTAGTATAGTGGTCAGTA | 18 | fox_milRNA_7 | 2 | 0 | 0 | 0 | 0 | 0 | 3 | 1 | 0 | 0 | 0 | 0 | 0 | 1 | 1 | 0 | 0 | 0 | 0 | 1 | 0 | 0 | 0 | 0 |
| GTAGTATAGTGGTCAGTAT | 19 | fox_milRNA_7 | 1 | 0 | 0 | 0 | 0 | 0 | 1 | 0 | 1 | 0 | 0 | 0 | 0 | 0 | 0 | 0 | 0 | 0 | 0 | 1 | 0 | 0 | 0 | 0 |
| GTAGTATAGTGGTCAGTATG | 20 | fox_milRNA_7 | 6 | 0 | 2 | 0 | 0 | 0 | 3 | 2 | 1 | 0 | 0 | 0 | 3 | 1 | 3 | 0 | 0 | 0 | 2 | 1 | 1 | 0 | 0 | 0 |
| GTAGTATAGTGGTCAGTATGC | 21 | fox_milRNA_7 | 1 | 0 | 0 | 0 | 0 | 0 | 0 | 0 | 0 | 0 | 0 | 0 | 0 | 0 | 0 | 0 | 0 | 0 | 0 | 1 | 0 | 0 | 0 | 0 |
| GTAGTATAGTGGTCAGTATGCA | 22 | fox_milRNA_7 | 0 | 1 | 0 | 0 | 0 | 0 | 0 | 0 | 1 | 0 | 0 | 0 | 1 | 0 | 0 | 0 | 0 | 0 | 1 | 0 | 1 | 0 | 0 | 0 |
| GTATAGTGGTCAGTATGC | 18 | fox_milRNA_7 | 0 | 0 | 0 | 0 | 0 | 0 | 0 | 1 | 0 | 0 | 0 | 0 | 0 | 0 | 0 | 0 | 0 | 0 | 0 | 0 | 0 | 0 | 0 | 0 |
| GTATAGTGGTCAGTATGCA | 19 | fox_milRNA_7 | 0 | 0 | 0 | 0 | 0 | 0 | 0 | 0 | 0 | 0 | 0 | 0 | 0 | 0 | 0 | 0 | 0 | 0 | 0 | 1 | 0 | 0 | 0 | 0 |
| TAGTATAGTGGTCAGTATG | 19 | fox_milRNA_7 | 1 | 1 | 0 | 0 | 0 | 0 | 0 | 1 | 0 | 0 | 1 | 0 | 1 | 1 | 0 | 0 | 0 | 0 | 0 | 2 | 0 | 0 | 0 | 0 |
| TAGTATAGTGGTCAGTATGC | 20 | fox_milRNA_7 | 0 | 0 | 0 | 0 | 0 | 0 | 1 | 1 | 0 | 0 | 0 | 0 | 0 | 0 | 0 | 0 | 0 | 0 | 0 | 0 | 0 | 0 | 0 | 0 |
| TAGTATAGTGGTCAGTATGCA | 21 | fox_milRNA_7 | 0 | 0 | 0 | 0 | 0 | 0 | 0 | 0 | 1 | 0 | 0 | 0 | 0 | 0 | 0 | 0 | 0 | 0 | 0 | 1 | 0 | 0 | 0 | 0 |
| TATAGTGGTCAGTATGCA | 18 | fox_milRNA_7 | 0 | 0 | 1 | 0 | 0 | 0 | 0 | 0 | 0 | 0 | 0 | 0 | 0 | 0 | 0 | 0 | 0 | 0 | 0 | 0 | 0 | 0 | 0 | 0 |
| TCCGTAGTATAGTGGTCAG | 19 | fox_milRNA_7 | 2 | 0 | 2 | 0 | 0 | 0 | 2 | 6 | 1 | 0 | 0 | 0 | 0 | 1 | 3 | 0 | 0 | 0 | 0 | 0 | 1 | 0 | 0 | 0 |
| TCCGTAGTATAGTGGTCAGT | 20 | fox_milRNA_7 | 1 | 1 | 3 | 0 | 0 | 0 | 2 | 1 | 3 | 0 | 0 | 0 | 1 | 0 | 2 | 0 | 0 | 0 | 3 | 1 | 1 | 0 | 0 | 0 |
| TCCGTAGTATAGTGGTCAGTA | 21 | fox_milRNA_7 | 0 | 0 | 2 | 0 | 0 | 0 | 1 | 4 | 1 | 0 | 0 | 0 | 0 | 1 | 1 | 0 | 0 | 0 | 1 | 1 | 3 | 0 | 0 | 0 |
| TCCGTAGTATAGTGGTCAGTAT | 22 | fox_milRNA_7 | 3 | 0 | 1 | 0 | 0 | 0 | 3 | 2 | 0 | 0 | 0 | 0 | 3 | 0 | 1 | 0 | 0 | 0 | 0 | 0 | 1 | 0 | 0 | 0 |
| TCCGTAGTATAGTGGTCAGTATG | 23 | fox_milRNA_7 | 180 | 167 | 168 | 1 | 0 | 1 | 228 | 276 | 198 | 3 | 15 | 2 | 132 | 164 | 303 | 0 | 0 | 1 | 175 | 273 | 207 | 2 | 0 | 0 |
| TCCGTAGTATAGTGGTCAGTATGC | 24 | fox_milRNA_7 | 3 | 1 | 0 | 0 | 0 | 0 | 6 | 2 | 2 | 0 | 1 | 0 | 2 | 0 | 1 | 0 | 0 | 0 | 3 | 4 | 2 | 0 | 0 | 0 |
| TCCGTAGTATAGTGGTCAGTATGCA | 25 | fox_milRNA_7 | 16 | 8 | 13 | 0 | 0 | 0 | 16 | 13 | 8 | 0 | 2 | 0 | 3 | 10 | 7 | 0 | 0 | 0 | 9 | 9 | 6 | 0 | 0 | 0 |
| TTCCGTAGTATAGTGGTCA | 19 | fox_milRNA_7 | 1 | 0 | 1 | 0 | 0 | 0 | 0 | 0 | 0 | 0 | 0 | 0 | 0 | 0 | 0 | 0 | 0 | 0 | 0 | 0 | 0 | 0 | 0 | 0 |
| TTCCGTAGTATAGTGGTCAG | 20 | fox_milRNA_7 | 4 | 2 | 2 | 0 | 0 | 0 | 0 | 0 | 0 | 0 | 0 | 0 | 0 | 0 | 1 | 0 | 0 | 0 | 4 | 2 | 1 | 0 | 0 | 0 |
| TTCCGTAGTATAGTGGTCAGT | 21 | fox_milRNA_7 | 0 | 0 | 1 | 0 | 0 | 0 | 0 | 0 | 0 | 0 | 0 | 0 | 0 | 0 | 1 | 0 | 0 | 0 | 0 | 0 | 1 | 0 | 0 | 0 |
| TTCCGTAGTATAGTGGTCAGTA | 22 | fox_milRNA_7 | 1 | 0 | 0 | 0 | 0 | 0 | 1 | 1 | 3 | 0 | 0 | 0 | 0 | 1 | 0 | 0 | 0 | 0 | 0 | 1 | 1 | 0 | 0 | 0 |
| TTCCGTAGTATAGTGGTCAGTAT | 23 | fox_milRNA_7 | 1 | 0 | 2 | 0 | 0 | 0 | 2 | 1 | 0 | 0 | 0 | 0 | 0 | 0 | 0 | 0 | 0 | 0 | 2 | 0 | 0 | 0 | 0 | 0 |
| TTCCGTAGTATAGTGGTCAGTATG | 24 | fox_milRNA_7 | 56 | 42 | 44 | 0 | 0 | 0 | 63 | 52 | 53 | 2 | 4 | 0 | 47 | 54 | 96 | 0 | 3 | 0 | 45 | 79 | 62 | 0 | 0 | 0 |
| TTCCGTAGTATAGTGGTCAGTATGC | 25 | fox_milRNA_7 | 0 | 3 | 2 | 0 | 0 | 0 | 5 | 3 | 3 | 0 | 1 | 0 | 1 | 1 | 1 | 0 | 0 | 0 | 2 | 2 | 1 | 0 | 0 | 0 |
| TTCCGTAGTATAGTGGTCAGTATGCA | 26 | fox_milRNA_7 | 5 | 5 | 7 | 0 | 0 | 0 | 7 | 6 | 4 | 0 | 2 | 0 | 3 | 4 | 1 | 0 | 0 | 0 | 2 | 8 | 5 | 0 | 0 | 0 |
|  |  | Total | 420 | 331 | 345 | 3 | 0 | 1 | 566 | 625 | 439 | 8 | 34 | 4 | 283 | 309 | 555 | 0 | 3 | 3 | 440 | 681 | 531 | 4 | 0 | 1 |
| CTTGAGACCCGGGTTCAA | 18 | fox_milRNA_8 | 0 | 1 | 0 | 0 | 0 | 0 | 0 | 0 | 0 | 0 | 0 | 0 | 0 | 0 | 0 | 0 | 0 | 0 | 0 | 0 | 0 | 0 | 0 | 0 |
| CTTGAGACCCGGGTTCAATTC | 21 | fox_milRNA_8 | 0 | 0 | 0 | 0 | 0 | 0 | 1 | 0 | 0 | 0 | 0 | 0 | 0 | 0 | 0 | 0 | 0 | 0 | 0 | 0 | 0 | 0 | 0 | 0 |
| CTTGAGACCCGGGTTCAATTCCC | 23 | fox_milRNA_8 | 0 | 1 | 0 | 0 | 0 | 0 | 0 | 0 | 0 | 0 | 0 | 0 | 0 | 0 | 0 | 0 | 0 | 0 | 0 | 0 | 0 | 0 | 0 | 0 |
| CTTGAGACCCGGGTTCAATTCCCGGC | 26 | fox_milRNA_8 | 0 | 1 | 0 | 0 | 0 | 0 | 0 | 2 | 1 | 0 | 0 | 0 | 0 | 0 | 0 | 0 | 0 | 0 | 0 | 0 | 0 | 0 | 0 | 0 |
| GAGACCCGGGTTCAATTCCC | 20 | fox_milRNA_8 | 0 | 0 | 0 | 0 | 0 | 0 | 1 | 0 | 0 | 1 | 0 | 0 | 0 | 0 | 0 | 0 | 0 | 0 | 0 | 1 | 0 | 0 | 0 | 0 |
| TGAGACCCGGGTTCAATTC | 19 | fox_milRNA_8 | 0 | 1 | 0 | 0 | 0 | 0 | 0 | 0 | 0 | 0 | 0 | 0 | 0 | 0 | 0 | 0 | 0 | 0 | 0 | 0 | 0 | 0 | 0 | 0 |
| TGAGACCCGGGTTCAATTCCC | 21 | fox_milRNA_8 | 0 | 0 | 0 | 0 | 0 | 0 | 1 | 0 | 0 | 0 | 0 | 0 | 0 | 0 | 0 | 0 | 0 | 0 | 1 | 0 | 0 | 0 | 0 | 0 |
| TGAGACCCGGGTTCAATTCCCGGC | 24 | fox_milRNA_8 | 0 | 0 | 0 | 0 | 0 | 0 | 0 | 0 | 2 | 0 | 0 | 0 | 0 | 0 | 0 | 0 | 0 | 0 | 0 | 0 | 1 | 0 | 0 | 0 |
| TTGAGACCCGGGTTCAATTCCC | 22 | fox_milRNA_8 | 0 | 1 | 1 | 0 | 0 | 0 | 1 | 0 | 0 | 0 | 0 | 0 | 1 | 0 | 1 | 0 | 0 | 0 | 0 | 0 | 0 | 0 | 0 | 0 |
| TTGAGACCCGGGTTCAATTCCCGGC | 25 | fox_milRNA_8 | 1 | 0 | 0 | 0 | 0 | 0 | 0 | 1 | 1 | 0 | 0 | 0 | 0 | 0 | 0 | 0 | 0 | 0 | 0 | 0 | 0 | 0 | 0 | 0 |
|  |  | Total | 1 | 5 | 1 | 0 | 0 | 0 | 4 | 3 | 4 | 1 | 0 | 0 | 1 | 0 | 1 | 0 | 0 | 0 | 1 | 1 | 1 | 0 | 0 | 0 |
