# Supplementary figures and images for: Comparative Transcriptome Profiles of Near-Isogenic Hexaploid Wheat Lines Differing for Effective Alleles at the 2DL FHB Resistance QTL
Source: Front Plant Sci. 2018 Jan 30;9:37. doi: 10.3389/fpls.2018.00037 (PMC5797473; doi:10.3389/fpls.2018.00037)

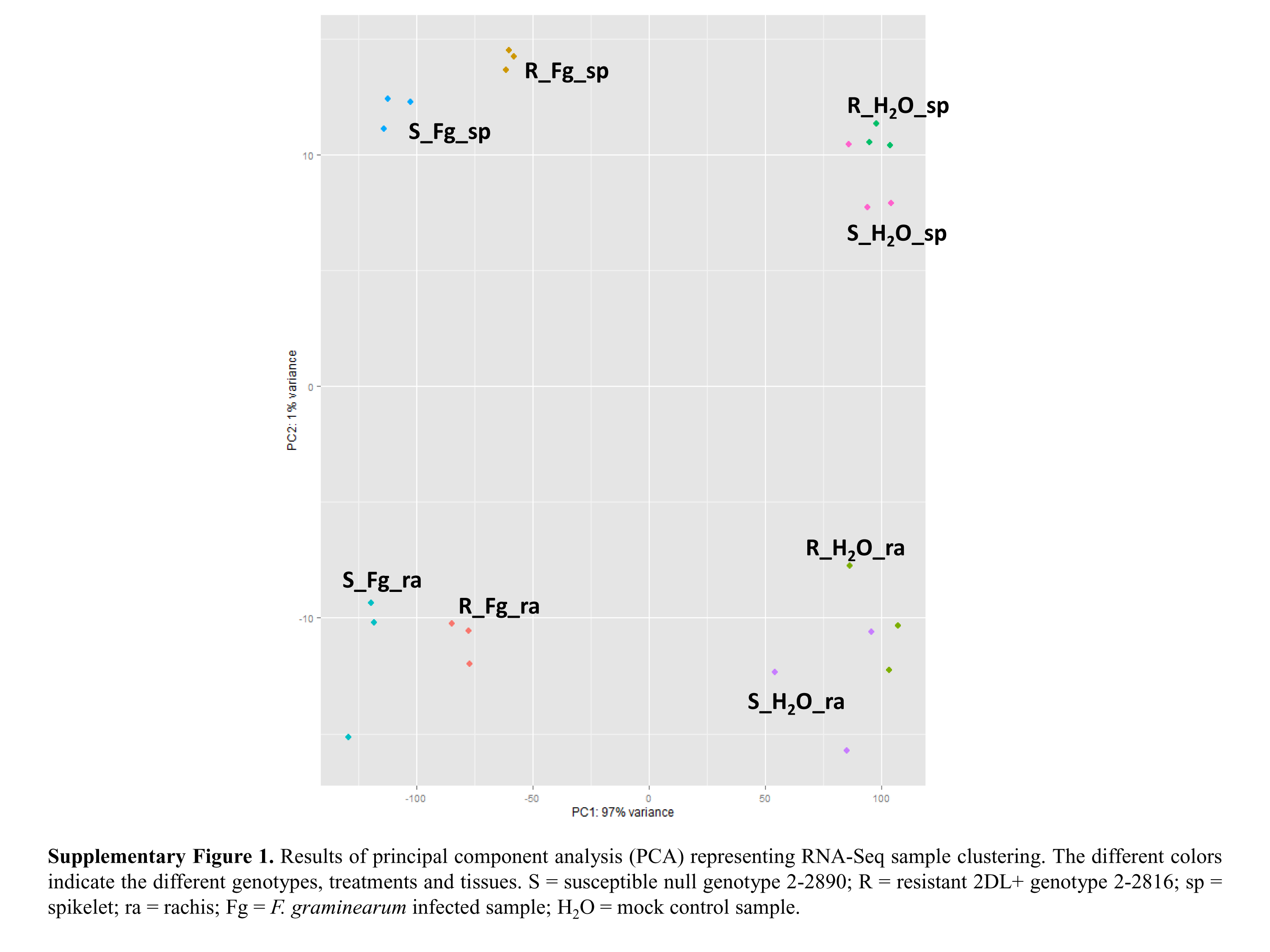

Supplement: Supplementary file 6 [file Image1.TIF]

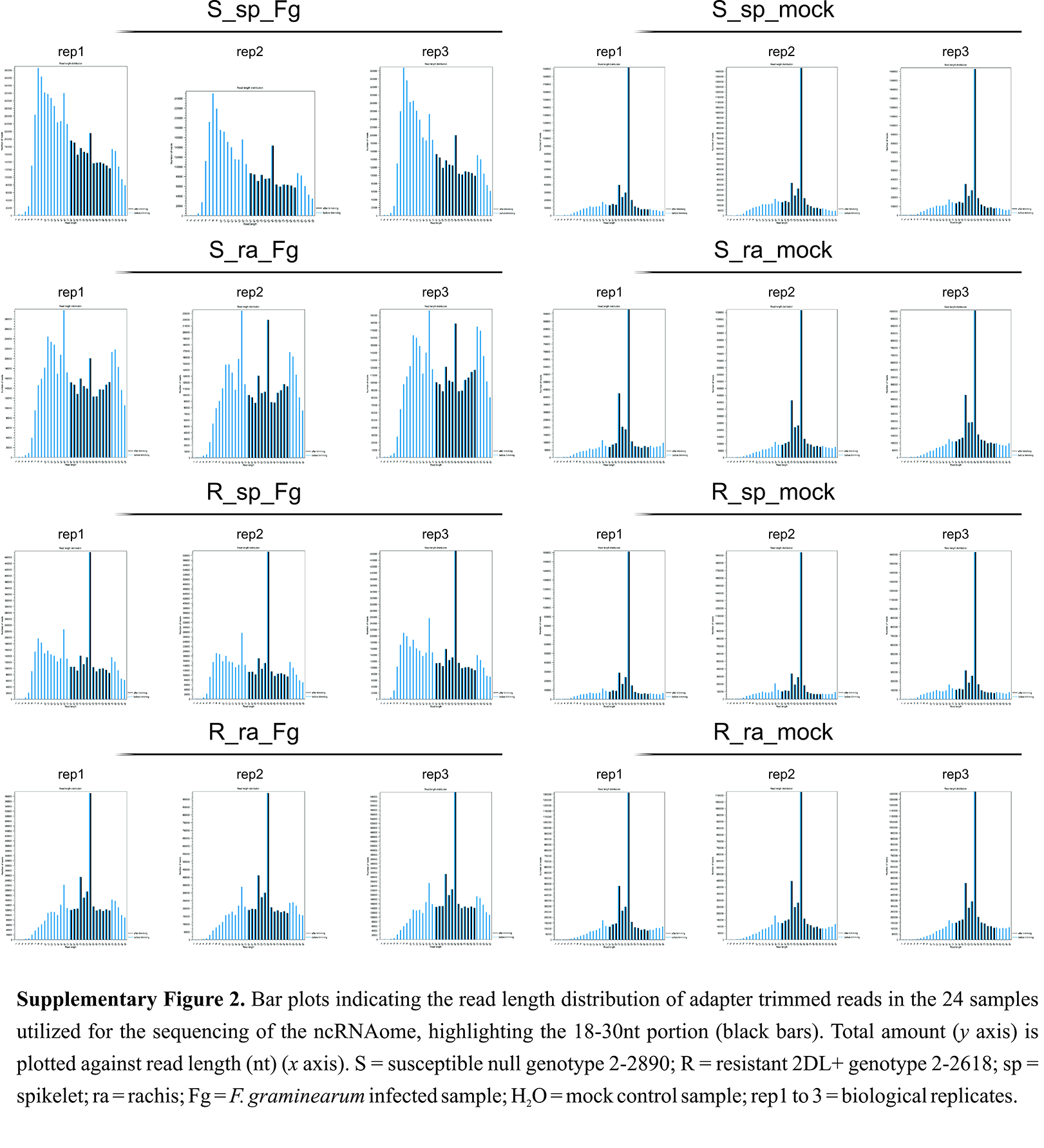

Supplement: Supplementary file 7 [file Image2.TIF]

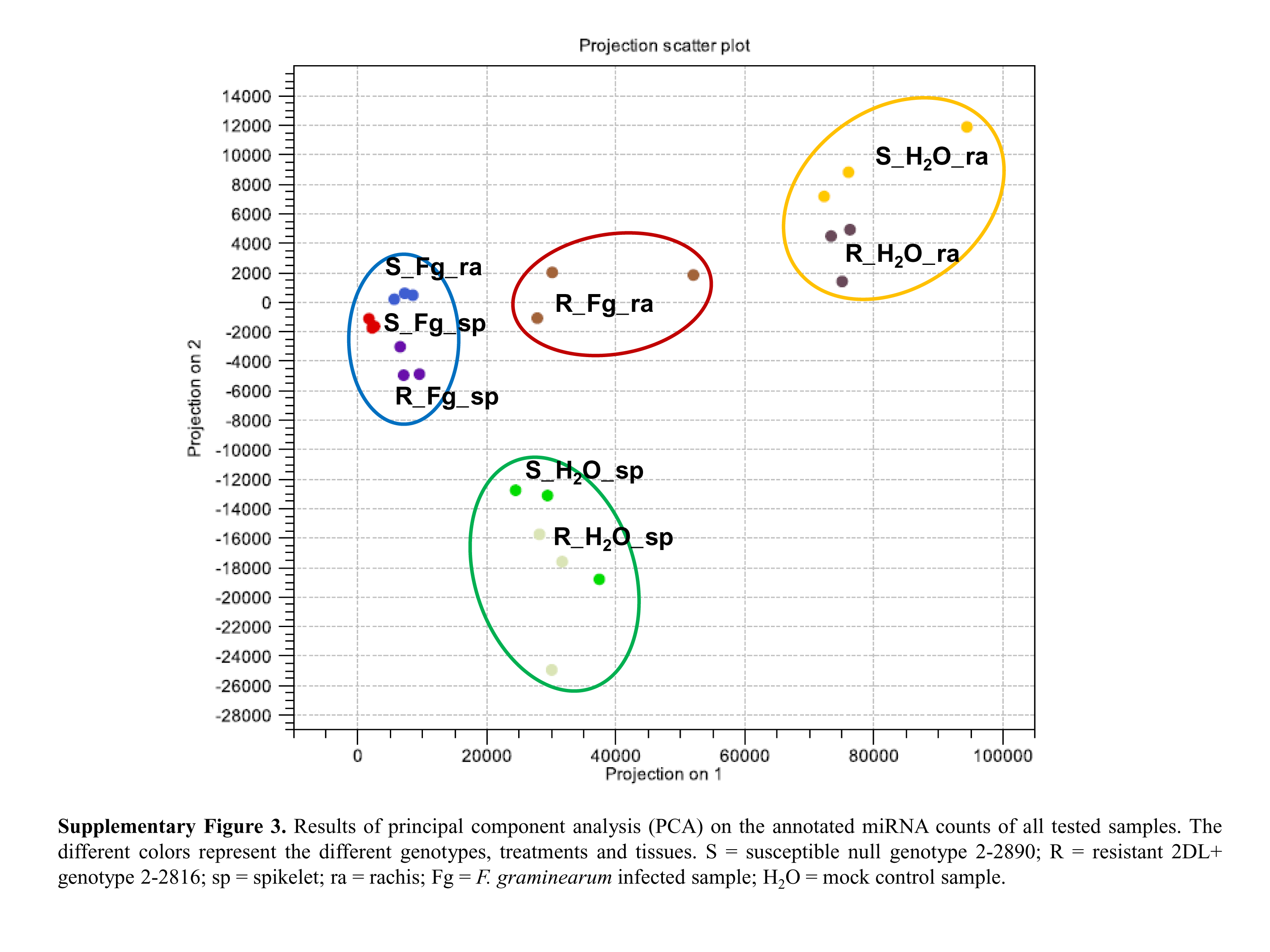

Supplement: Supplementary file 8 [file Image3.TIF]
